# PEGylated Purpurin 18 with Improved Solubility: Potent Compounds for Photodynamic Therapy of Cancer

**DOI:** 10.3390/molecules24244477

**Published:** 2019-12-06

**Authors:** Vladimíra Pavlíčková, Silvie Rimpelová, Michal Jurášek, Kamil Záruba, Jan Fähnrich, Ivana Křížová, Jiří Bejček, Zdeňka Rottnerová, Vojtěch Spiwok, Pavel Drašar, Tomáš Ruml

**Affiliations:** 1 Department of Biochemistry and Microbiology, University of Chemistry and Technology in Prague, Technická 3, 166 28 Prague 6, Czech Republic; vladimira.pavlickova@vscht.cz (V.P.); jiri.bejcek@vscht.cz (J.B.); vojtech.spiwok@vscht.cz (V.S.); 2 Department of Chemistry of Natural Compounds, University of Chemistry and Technology in Prague, Technická 5, 166 28 Prague 6, Czech Republic; michal.jurasek@vscht.cz; 3 Department of Analytical Chemistry, University of Chemistry and Technology in Prague, Technická 5, 166 28 Prague 6, Czech Republic; kamil.zaruba@vscht.cz (K.Z.); jan.fahnrich@vscht.cz (J.F.); 4 Department of Biotechnology, University of Chemistry and Technology in Prague, Technická 5, 166 28 Prague 6, Czech Republic; ivana.krizova@vscht.cz; 5 Central laboratories, University of Chemistry and Technology in Prague, Technická 5, 166 28 Prague 6, Czech Republic; zdenka.rottnerova@vscht.cz

**Keywords:** apoptosis, cancer cells, cytotoxicity, flow cytometry, live-cell fluorescence microscopy, PEGylated purpurin 18, photodynamic therapy, photosensitizer, phototoxicity, singlet oxygen

## Abstract

Purpurin 18 derivatives with a polyethylene glycol (PEG) linker were synthesized as novel photosensitizers (PSs) with the goal of using them in photodynamic therapy (PDT) for cancer. These compounds, derived from a second-generation PS, exhibit absorption at long wavelengths; considerable singlet oxygen generation and, in contrast to purpurin 18, have higher hydrophilicity due to decreased logP. Together, these properties make them potentially ideal PSs. To verify this, we screened the developed compounds for cell uptake, intracellular localization, antitumor activity and induced cell death type. All of the tested compounds were taken up into cancer cells of various origin and localized in organelles known to be important PDT targets, specifically, mitochondria and the endoplasmic reticulum. The incorporation of a zinc ion and PEGylation significantly enhanced the photosensitizing efficacy, decreasing IC_50_ (half maximal inhibitory compound concentration) in HeLa cells by up to 170 times compared with the parental purpurin 18. At effective PDT concentrations, the predominant type of induced cell death was apoptosis. Overall, our results show that the PEGylated derivatives presented have significant potential as novel PSs with substantially augmented phototoxicity for application in the PDT of cervical, prostate, pancreatic and breast cancer.

## 1. Introduction

Chlorins are natural photosensitive chlorophyll derivatives containing twenty π electrons in the aromatic ring. Various modified substructures derived from their basic core have been discovered within the plant kingdom [1,2,3,4]. Owing to their strong absorption between 650–700 nm, wavelengths that penetrate tissue effectively, chlorins have been investigated as photosensitizers (PSs) for use in the photodynamic therapy (PDT) of cancerous and noncancerous diseases [5,6,7,8].

During the PDT treatment of cancer, ubiquitous oxygen in the triplet state turns into highly reactive singlet oxygen [9] that triggers cell death via oxidative damage to proteins, lipids and other cellular content, resulting in apoptosis [7,10], necrosis [11] and/or autophagy [12]. In addition to these direct mechanisms of tumor elimination, PDT leads to microvascular damage [13], which is a significant advantage over traditionally used treatments, such as chemo- and radiotherapy. Moreover, PDT also induces immunogenic cell death by stimulating the immune system response to the tumor [13]. PS-induced phototoxic damage initiates the release of anti-inflammatory mediators that attract neutrophils and other immune cells [14]. Indeed, a PS can even trigger adaptive immunity leading to long-term immune response [15].

Chlorins possess optimal properties for use in PDT but are rather hydrophobic and, thus, aggregate in aqueous media, limiting their application. Consequently, various chemical modifications of chlorin-based PSs have been investigated with the aim of improving their physico-chemical characteristics: core metalation [16]; PEGylation [17,18,19,20]; conjugation with peptides [21,22,23,24], amino acids [1,25,26,27], sugars [28,29,30,31], choline [7,32] and gold nanoparticles [32].

**Scheme 1:**
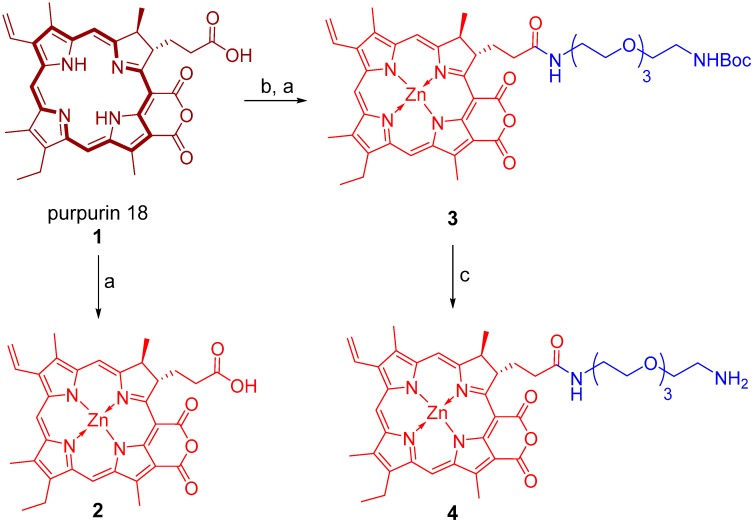
Synthesis of derivatives of PEGylated purpurin 18 (compound **1**). Reagents and conditions: (**a**) Zn(OAc)_2_·2H_2_O, MeOH, CHCl_3_, 50 °C, 13 h; yield of compound **2** was 61%; (**b**) DIC, EDIPA, THF, HOBt, 24 h, RT (22 °C); yield of compound **3** was 41% over two steps; (**c**) TFA, wet DCM, 1 h, RT (22 °C); yield of compound **4** was 56%.

A chlorin worth further derivatization is purpurin 18 (compound **1**, Scheme 1), which comprises a fused anhydride and an aliphatic side chain terminated with a carboxylic group. With its strong absorption at 700 nm and good singlet oxygen quantum yield (0.7) [33], this PS has been previously evaluated as a highly potent inductor of PDT-mediated cell death [6,34,35]. Nevertheless, in its natural form, its hydrophobicity causes aggregation at physiological pH and, thus, preferential localization in compartments undesirable for PDT, such as lipid vesicles and lysosomes. Moreover, under the in vivo conditions of PDT, the anhydride ring moiety is readily hydrolyzed into another PS chlorin, p6 [36], which is less effective than compound **1** [34].

However, despite the drawbacks associated with compound **1**, the natural advantages of purpurins makes it worthwhile to investigate the modification of this chlorin. Therefore, we here synthesize and evaluate PEGylated derivatives of compound **1** as novel PDT agents. We show that the attachment of short PEG_3_ moieties terminated by Boc (**3**) or an amino group (**4**) via an amide bond to the zinc chelate of purpurin 18 (**2**) does not hamper its ability to generate singlet oxygen in cell culture media in vitro; in fact, it actually enhances singlet oxygen generation, and photodynamic efficiency, by a factor of at least two. Furthermore, live-cell imaging showed that the PEGylation of compound **1** improves PS accumulation in the mitochondria and endoplasmic reticulum, the preferred targets for PDT drugs; in the case of compound 4, it also improves PS accumulation in lysosomes. Moreover, compound phototoxicity and dark toxicity were compared in six cancerous cell lines using WST-1 assay. These tests confirmed the increased PDT efficacy of the PEGylated analogues of compound **1**; these analogues also augmented the proportion of apoptotic cells when photoactivated. In addition, we show that these novel compounds have enhanced hydrophilicity (calculated) and are weaker binders of the prevalent transport protein, human serum albumin (HSA), than the parental compound.

## 2. Results and Discussion

### 2.1. Synthesis of Purpurin 18 Derivatives

The single carboxylic moiety of compound **1** was chosen as the site of synthetic modifications to its structure. A purpurin zinc complex (**2**) was prepared as described by Olshevskaya et al. [37]. Purpurin-18-PEG_3_-amine conjugates **3** and **4** were synthesized in three steps (see Scheme 1). The conjugation of Boc-protected PEG_3_-diamine to **1** was performed using carbodiimide chemistry. *N*, *N*-diisopropylcarbodiimide (DIC) with *N*-hydroxybenzotriazole (HOBt) and Hünig’s base (EDIPA) were used as the coupling conditions. PEGylated compound **1** was only filtered through a silica plug and the first dark band collected as crude product. Zinc was inserted into the chlorin core using zinc(II) acetate as a metal donor and product **2** was purified by two-step column chromatography with a yield of 41%. The Boc protecting group was cleaved by an excess of trifluoroacetic acid (TFA) in wet dichloromethane (DCM) to obtain amine **4** with a yield of 56%. The obtained products were lyophilized from aqueous dioxane and stored in a fridge in the dark. The acquired spectra are shown in Supplementary Information (SI, Figures S1–S6-2), Section 1.

### 2.2. Singlet Oxygen Generation

The quantum yield of singlet oxygen production by the PSs was evaluated using absorption spectrometry, with 9,10-anthracenediyl-bis(methylene)dimalonic acid (**AB**) as the probe. The PS-mediated singlet oxygen production was monitored by decreases in the absorbance of **AB** at 381 and 403 nm, which were due to the formation of the corresponding endoperoxide [38,39]. There was a negligible decrease in AB absorption without PS (SI, Figure S6-3). The rate of a decrease in **AB** relative absorbance was considered to be proportional to singlet oxygen production.

The singlet oxygen quantum yield for a tested compound (*ϕ*_x_) was compared with the known quantum yield (*ϕ*_s_) of a standard

**Figure 1:**
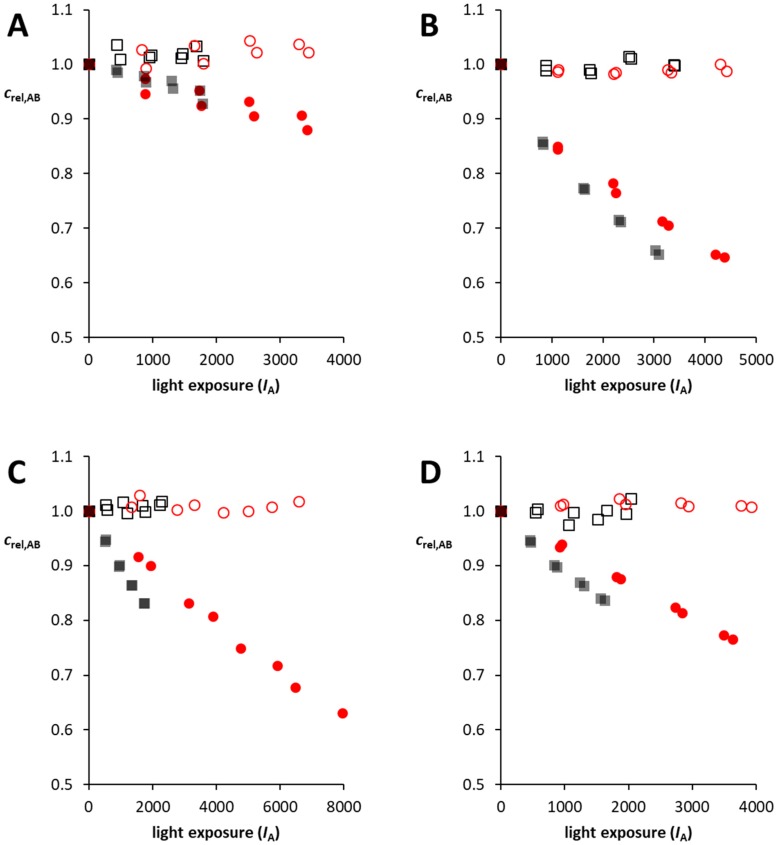
Depletion of 9,10-anthracenediyl-bis(methylene)dimalonic acid (**AB**, 7 × 10^−5^ M) with photosensitizer-generated singlet oxygen in Dulbecco’s Modified Eagle Medium with 10% fetal bovine serum. Photosensitizers: (**A**) compound **1** (7.7 × 10^−6^ M, 1.5 × 10^−5^ M), (**B**) compound **2** (8.0 × 10^−6^ M, 1.6 × 10^−5^ M), (**C**) compound **3** (7.2 × 10^−6^ M, 1.4 × 10^−5^ M), (**D**) compound **4** (7.5 × 10^−6^ M, 1.5 × 10^−5^ M). The experiments were duplicated. 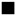 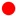
—Solution exposed to light, 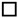 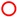
—Solution kept in dark. c_rel,AB_—Relative concentration of **AB** (actual concentration with respect to concentration at experiment start).

*ϕ*_x_ = *ϕ*_s_*γ*_x_/*γ*_s_

where *γ*_x_ and *γ*_s_ are chemical photodynamic efficiencies of the tested and standard compounds, respectively, evaluated from the **AB** absorbance decrease plotted against relative light exposure (*I*_A_) (Figure 1). Using the singlet oxygen quantum yield of Rose Bengal (**RB**) in phosphate buffered saline *ϕ*_s_ = 0.75 [40,41], quantum yields for **RB** and studied compounds **1**–**4** were evaluated in Dulbecco’s Modified Eagle Medium with fetal bovine serum (DMEM+FBS) (Table 1, SI Figure S6-4).

### 2.3. Uptake and Intracellular Localization of the Compounds

**Figure 2:**
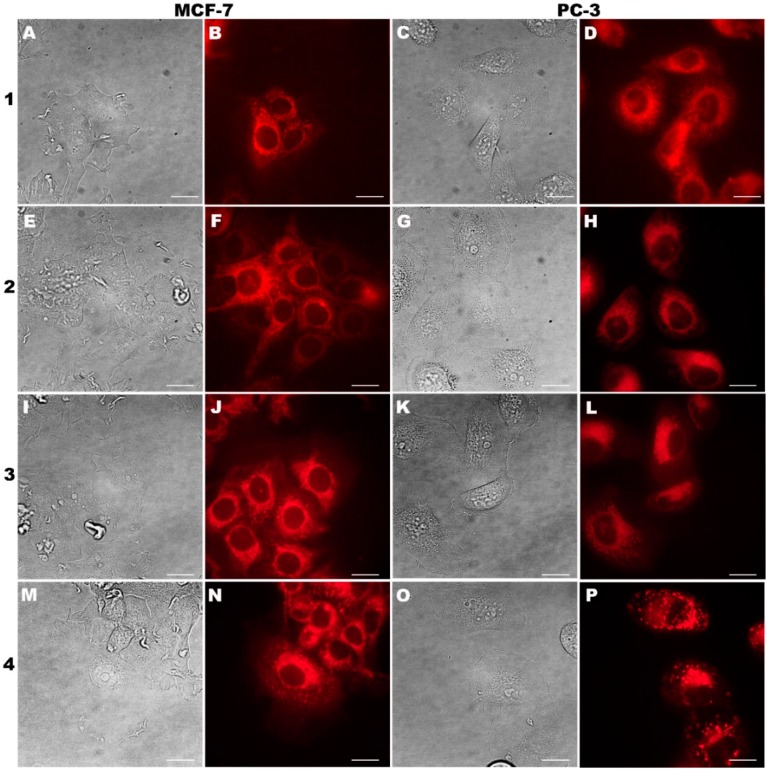
Fluorescence microscopy images of intracellular localization of purpurin 18 (compound **1**) and its derivatives (compounds **2**–**4**) at 0.5 µM concentration in human cancer cell lines of MCF-7 (breast carcinoma) and PC-3 (prostate carcinoma) after 24 h incubation. In the first and third columns, there are bright field images; the second and fourth columns show compound localization. The scale bars represent 20 µm.

The ability of a PS to cross the plasma membrane is the initial prerequisite for good PDT efficacy [42,43]. Therefore, using live-cell fluorescence microscopy, we determined the ability of compound **1** and its derivatives **2**–**4** (0.2 to 2 µM) to accumulate in human cells of various origin after 3, 16 and 24 h. Cell lines derived from breast (MCF-7), prostate (PC-3, LNCaP) and cervical (HeLa) carcinoma, as well as from pancreatic adenocarcinoma (MiaPaCa-2) and immortalized human keratinocytes (HaCaT), were used. Based on the microscopic images (Figure 2, SI, Figure S7), it is clear that the efficacy of the cell uptake of the individual compounds varied. At the same concentration and incubation time, the fluorescence emission intensities (Table 2; SI, Figure S16) of compounds **1** and **2** were weaker than those of PEGylated derivatives **3** and **4** (data in Table 2 for PC-3 cells). Compared with compounds **3** and **4**, the low fluorescence emission intensities of compounds **1** and **2** might be caused by their less efficient penetration through the plasma membrane and/or by faster efflux. In turn, this could be due to their distinct molecule sizes as well as to differences in their lipophilicity, which is one of the key factors for compound penetration through cell membranes. The lipophilicity of compounds may be enhanced at the lower pH of cancer cells. This has been documented by the increased uptake of hematoporphyrin at lower than physiological pH [44,45], though it was not observed for mTHPP, mTHPC and TPPS2a [45]. Similarly, Sharma et al. reported the augmented cell uptake of chlorin p6 at decreased pH for Colo-205 cells, but not for MCF-7 cells [46]. Therefore, apart from being pH dependent, the cell uptake of a PS is also cell line specific. This corresponds with the uptake and intracellular localization of compounds **1**–**4** differing both among the tested compounds and evaluated cell lines.

Sharma et al. [36] reported that the aggregation of compound **1** (6 µM) led to its limited availability. Nevertheless, probably due to the lower concentration used (0.5 µM), we did not observe any aggregation of this compound but, rather, homogenous localization in the intracellular space of the HaCaT, LNCaP and PC-3 cells (Figure 2 and SI, Figure S7) after 3 h. In the MCF-7 cells (Figure 2), compounds **1**–**3** localized in organelles visible as a network-like structure. Compound **4** localized in the HaCaT and PC-3 cells, preferentially in small vesicles with high fluorescence intensity. Regarding the MCF-7 cell line, compound **4** localized in both a network-like structure and in small vesicles with high fluorescence intensity.

### 2.4. Colocalization Study

**Figure 3:**
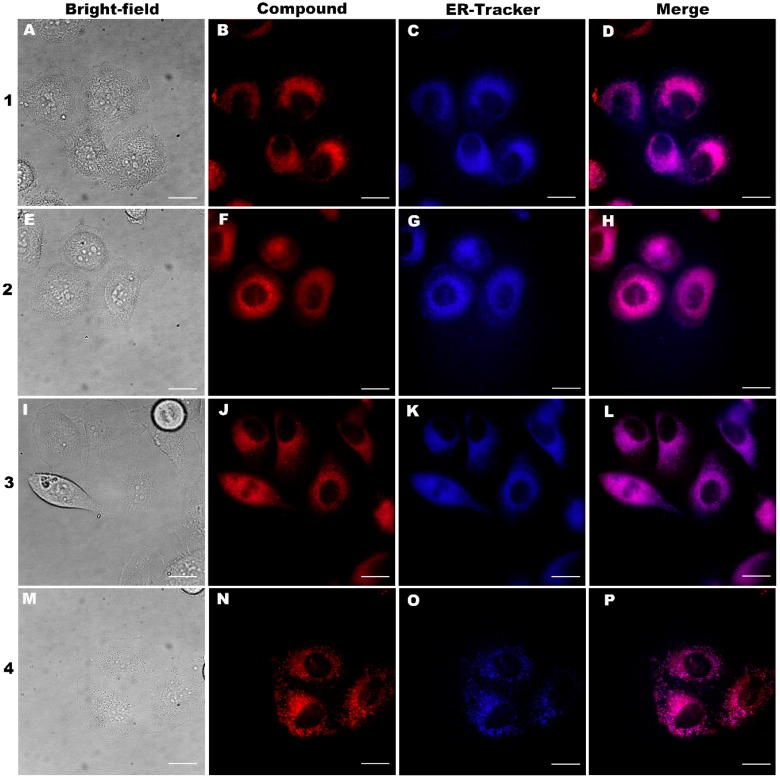
Fluorescence microscopy images of localization of purpurin 18 (compound **1**) and its derivatives (compounds **2**–**4**) in the endoplasmic reticulum of human PC-3 cells derived from prostate carcinoma. Colocalization of compounds **1**–**2** (0.5 µM, 24 h) or compounds **3**–**4** (0.5 µM, 24 h) with ER-Tracker™ Blue-White DPX (70 nM, 30 min). (**A**,**E**,**I**,**M**) Bright-field images; (**B**,**F**,**J**,**N**) localization of the tested compounds; (**C**,**G**,**K**,**O**) ER-Tracker™ Blue-White DPX; (**D**,**H**,**L**,**P**) merged fluorescent images. The scale bars represent 20 µm.

**Figure 4:**
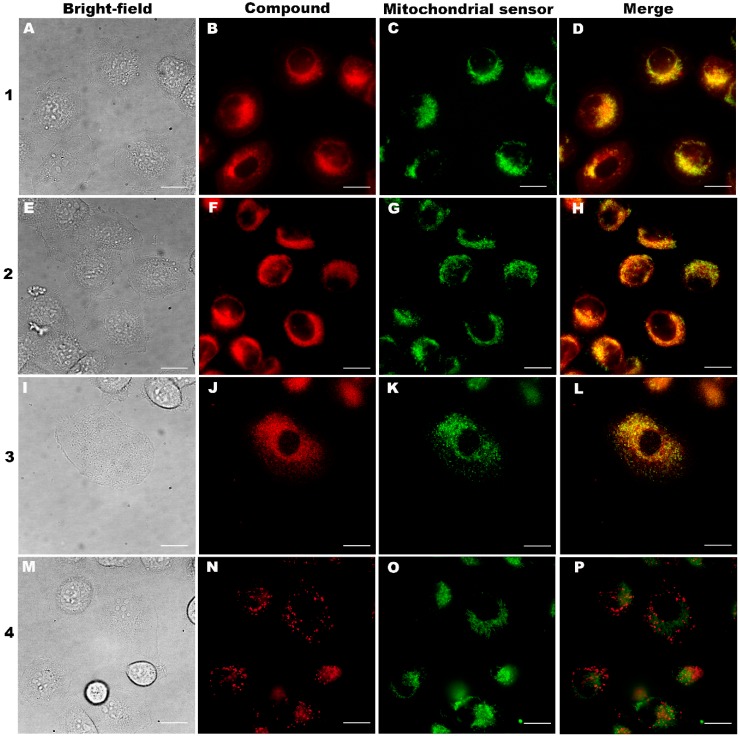
Fluorescence microscopy images of localization of purpurin 18 (compound **1**) and its derivatives (compounds **2**–**4**) in the mitochondria of human PC-3 cells derived from prostate carcinoma. Colocalization of compounds **1**–**2** (0.5 µM, 3 h) or compounds **3**–**4** (0.5 µM, 3 h) with a mitosensor (70 nM, 10 min) based on our patented dimethinium salt [47]. (**A**,**E**,**I**,**M**) Bright-field images; (**B**,**F**,**J**,**N**) localization of the tested compounds; (**C**,**G**,**K**,**O**) mitosensor; (**D**,**H**,**L**,**P**) merge of the fluorescent images. The scale bars represent 20 µm.

**Figure 5:**
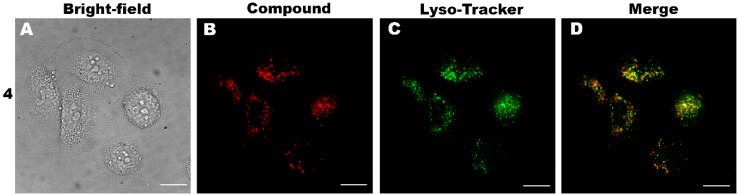
Fluorescence microscopy images of compound **4** localization in lysosomes of human PC-3 cells derived from prostate carcinoma. Colocalization of compound **4** (0.5 µM, 24 h) with LysoTracker Green DND-26 (70 nM, 20 min). (**A**) Bright-field images; (**B**) localization of compound **4**; (**C**) LysoTracker Green DND-26; (**D**) merge of the fluorescent images. The scale bars represent 20 µm.

To determine the exact intracellular localization of the tested compounds, commercial markers of cell organelles were used. Colocalization with the endoplasmic reticulum marker, ER-Tracker Blue-White DPX, was detected for all tested compounds in the PC-3 (Figure 3), MCF-7, LNCaP and HaCaT cells (SI, Figures S8–S10). Moreover, compounds **1**–**3** colocalized with mitochondrial sensors (MitoTracker Green and/or our patented green-emitting dimethinium salt [47]) in the PC-3 (Figure 4), MCF-7, LNCaP and HaCaT cells (SI, Figures S11–S13). Compound **4** also localized in the endoplasmic reticulum, but not in the mitochondria of the PC-3, MCF-7, LNCaP and HaCaT cells. Because another fluorescent signal not originating from the endoplasmic reticulum was surprisingly detected, further colocalization studies were performed. Using fluorescent markers of the Golgi apparatus (CellLight Golgi-GFP) and lysosomes (LysoTracker Green DND-26), lysosomal localization was confirmed, except in the Golgi apparatus (SI, Figure S14) of compound **4** in the HaCaT (SI, Figure S15), PC-3 (Figure 5) and MCF-7 cells.

The results for compound **1** correspond to those reported by other research groups focused on chlorophyll-derived PS photochemistry. The localization of purpurin 18 and its derivative chlorin p6 has been detected in the mitochondria, lysosomes and endoplasmic reticulum [19,42,48,49,50,51,52]. The localization of any PS in such organelles is key to high PDT efficacy.

### 2.5. Photo- and Dark Toxicity of the Compounds In Vitro

PSs **2**–**4** not only exhibited localization in preferable cell organelles (meaning that high PDT efficacy can be expected) but also produced good quantum yields (Table 1) exceeding those of compound **1** (Table 1). Therefore, we investigated their phototoxicity in human cancer cells. LNCaP, PC-3, MCF-7, U-2 OS (osteosarcoma), MIA PaCa-2 and HeLa cells were treated with compounds **1**–**4** (0.5–10 µM) for 24 h followed by light activation (light dose of 4 J·cm^−2^, 13 min) and incubation (a further 24 h). Dark toxicity (without photoactivation) was also evaluated for all compounds. Compound toxicity is expressed as a decrease in cell viability (SI, Figures S17 and 18) and by half maximal inhibitory compound concentration (IC_50_) values (Table 3).

Up to a concentration of 10 µM, compound **1** did not induce any dark toxicity in the MCF-7, PC-3, MIA PaCa-2 and U-2 OS cells. This corresponds to the assumption that compound **1** (and thereby potentially also its derivatives) has low dark toxicity, as reported for a number of human cancer cell lines (HL-60 [8], Colo-205 [36], Hep-G2 [42], A549 [53,54], MCF-7 [55]). Darmostuk et al. [6] determined that the IC_50_ (dark toxicity) of compound **1** exceeded 100 µM in HaCaT and VH10 cells and was 54 µM for NIH 3T3 cells. In our case, compound **1** did not exhibit dark toxicity up to 10 µM (the highest concentration tested), thus fulfilling a basic criterion for use in PDT. Likewise, the novel derivatives **2**–**4** did not display any dark toxicity (up to 10 µM), except in the case of compound **3** in the LNCaP and HeLa cells, whose IC_50_ values were 7.20 and 7.95 µM, respectively.

After light activation, a significant decrease in cell viability was observed, especially for compounds **3** and **4**. Compound **1** exhibited the highest phototoxicity in the prostatic cancer cell lines with IC_50_ values of 0.16 and 0.34 µM for the PC-3 and LNCaP cells, respectively. Regarding U-2 OS, MIA PaCa-2 and MCF-7, the phototoxic effect of compound **1** corresponded to IC_50_ values below 2 µM. Compound **2**, which contained a zinc ion but no PEG_3_ spacers, exhibited higher phototoxicity than compound **1** (IC_50_ = 1.04 µM) in the MIA PaCa-2 cells and slightly increased IC_50_ values for the prostatic cancer cell lines: 0.21 and 0.47 µM for PC-3 and LNCaP, respectively. Interestingly, PEGylated derivatives **3** and **4** of compound **1** manifested extraordinary phototoxicity in the LNCaP cells; IC_50_ values of 0.02 and 0.04 µM for compounds **4** and **3** were 18 and 9 times lower, respectively, than those for parental compound **1**. An even bigger difference in phototoxicity between the parental compound and its PEGylated derivatives was detected in the HeLa cells, for which there was an approximately 170- and 57-fold decrease in the IC_50_ values of compounds **4** and **3**, respectively. In contrast, up to 10 µM, compound **2** did not reach IC_50_ in the HeLa cells. The slightly increased phototoxicity of compounds **3** and **4** was also determined in the MIA PaCa-2 cells and, in the case of compound **4**, in the MCF-7 cells. Overall, the HeLa, LNCaP and MIA PaCa-2 cell lines were most sensitive to the PEGylated derivatives of compound **1**.

These results correspond to the compound localization determined by live-cell fluorescence microscopy, during which the highest fluorescence emission intensities were detected for compounds **3** and **4**. As previously reported [17,19,20,56,57], derivatization by a PEG spacer leads to increased compound hydrophilicity and, thus, to improved aqueous solubility, which in the case of porphyrins leads reduced aggregate formation. Thus, here the incorporation of a PEG_3_ spacer probably improved the bioavailability of the compounds, resulting in the augmented cell uptake of compounds **3** and **4** compared with compound **1** and its zinc derivative. Moreover, the presence of a zinc ion in the structure of compound **1** enhances absorption in the red region of the visible spectra, which facilitates deeper tissue penetration.

Similar to purpurinimides [58], compounds **1**–**4** passively diffused into the cells, but their intracellular localization differed after light treatment, upon which compounds **3** and **4** may have translocated to and/or become better sequestered in sensitive organelles, probably the mitochondria, thereby enhancing PDT efficacy. Another possible explanation for the enhanced efficiency of compounds **3** and **4** is that they induce different mechanisms of action and cell death type than compounds **1** and **2**. We investigate the latter below.

### 2.6. Evaluation of Cell Death

To date, three different mechanisms of PDT action in cancer have been proposed [59]: direct cell damage, vascular shutdown and immune response activation. The photoactivation of a PS results in an acute stress response that leads to changes in calcium ion concentration and lipid metabolism, as well as the production of cytokines and stress response mediators [60]. These responses hamper mitochondrial processes, resulting in reactive oxygen species production and, consequently, in damage to the mitochondrial membrane; such damage induces cytochrome c release into cytosol [7], which, in turn, causes apoptosis. In addition to apoptosis, at excessive PS concentrations, PDT can also lead to cell death via necrosis; sometimes, a combination of both apoptosis and necrosis is involved. What is not yet clear is the role of autophagy, which also can occur under certain PDT conditions. There is considerable disagreement between researchers regarding the effect of autophagy on PDT outcome, with some suggesting it enhances outcome and others that it inhibits efficacy; this debate is comprehensively reviewed in Mroz et al. [60] Generally, the prevalent cell death type is dependent not only on the structure, intracellular localization and concentration of a PS, but also on the light dose applied and on cell origin.

**Figure 6:**
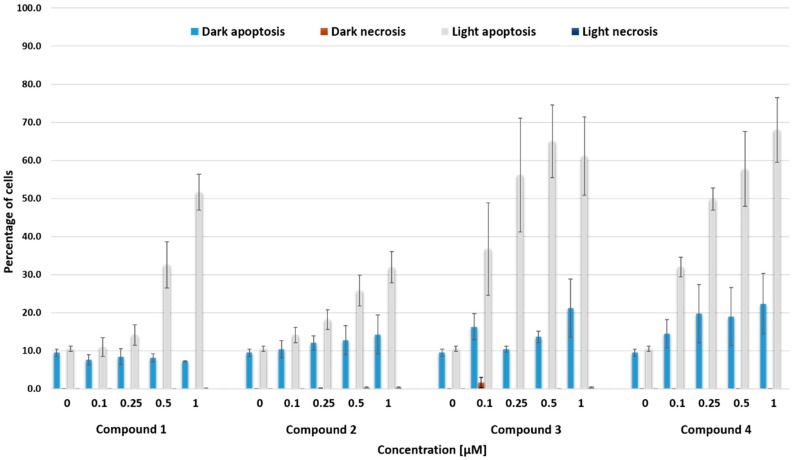
Dose-dependent mechanisms of cell death in MCF-7 cells induced by compounds **1**–**4** after 24-h treatment and light induction (Light) measured by flow cytometry. Control represents untreated cells and cells incubated with the same compounds without illumination (Dark). Total light dose was 4 J·cm^−2^. The data values and the errors are stated in Table S1 in Supplementary information.

To verify the mechanism of cell death induced by the tested PSs, MCF-7 cells were treated with compounds **1**–**4** (0.1–5 µM) for 24 h and photoactivated (light dose of 4 J·cm^−2^). After a further 24 h of incubation, the cells were stained with Annexin V and propidium iodide (PI) and cell death type was determined by flow cytometry. The controls (untreated photoactivated and nonphotoactivated cells) displayed a physiological level of ca. 10% of apoptotic cells, corresponding with [61,62], but exhibited no necrosis (Figure 6, SI Table S1). Similar results were detected for compound **1** at 0.1–1 µM concentration without photoactivation. However, at the highest tested concentration (5 µM, no photoactivation) while the proportion of apoptotic cells remained ca. 10%, necrotic cells accounted for ca. 11% of all cells (Figure 6, SI Table S1). At 5 µM, very similar results were observed for compounds **2**–**4**. At this highest concentration, the mechanism of cell death was probably governed by excessive PS concentration, corresponding to Stefano et al. [8]. At lower concentrations (0.1–1 µM) of compounds **2**–**4**, the proportion of apoptotic cells [ca. 10% to 22% compared with almost no necrotic cells (0% to 0.1%)] increased in direct dependence to concentration.

More interestingly, after photoactivation, the proportion of apoptotic cells among the MCF-7 cells treated with compound **1** rose to 52% as the concentration rose to 1 µM (Figure 6, SI Table S1). Under the same conditions, compound **2** induced apoptosis in 32% of cells. More promisingly, after photoactivation of the PEGylated derivatives of purpurin 18, compounds **3** and **4** (1 µM) triggered apoptosis in 61% and 68% of cells, respectively. Figure 6 and Table S1 in SI make it clear that the level of necrotic cells did not exceed 1.7% (mostly only 0.1%) after the photoactivation of compounds **1**–**4** (1 µM).

Researchers who have tested other PSs have reported similar results. For example, Stefano et al. [8] reported that HL-60 cells treated with a low concentration (0.2 µM) of photoactivated compound **1** (light dose of 1 J·cm^−2^) predominantly underwent apoptosis, while at higher concentrations (>2 µM) necrosis was prevalent. Tsai et al. showed that not only the concentration and the structure of a PS influences cell death type but also the light dose applied; for instance, at a light dose of 8 J·cm^−2^, 5-aminolevulic acid (ALA, 1 mM, 3 h; a precursor of protoporphyrin IX) induced the apoptosis of MCF-7 cells while a doubled light dose induced necrosis [63]. Light dose also affected cell death type in Sharma et al. [36], in which the 5 min light treatment (10 W·m^−2^) of compound **1** in liposomes caused the apoptosis of Colo-205 cells while 40 min treatment caused necrosis. These findings indicate that manipulating the desired type of cell death in PDT involves achieving a careful balance between PS type, PS concentration, light dose and cell line.

In summary, it is probable that both the site of PS localization and the initial location of PDT-related damage determine which cell death pathway is activated. It is possible that autophagy is initially activated to rescue the cells, but that later, when the PDT effect is sufficient and the cells are damaged beyond repair, apoptosis occurs [60]. Following this, at high PS doses, necrosis takes place, as the proteins participating in autophagy and apoptosis are destroyed and cellular integrity is lost.

### 2.7. Molecular Docking of Purpurin 18 Derivatives with Human Serum Albumin

To ensure that a PS is efficiently delivered to a pathological site in the body, it should interact with transport proteins, particularly albumins such as human serum albumin (HSA). HSA, one of the most abundant plasma proteins, is the key endogenous vehicle for the biodistribution of molecules by blood plasma [64]. Thus, we studied the association constants of compounds **1**–**4** by performing molecular docking with HSA.

The docked ligands, purpurin 18 and its three derivatives (Scheme 1), differed in zinc ion coordination (**2**–**4**) and PEG spacers (**3**–**4**). Moreover, the carboxyl group of compound **3** contained poly (ethylene glycol) diamine (PEGDA) with a *tert*-butyloxycarbonyl protecting group on the nitrogen atom; compound **4** was derivatized with PEGDA without a protecting group. The binding pocket for porphyrins in HSA is localized in its 1B domain [65,66]; similarly our ligands were docked to this domain, albeit with a different orientation. Regarding compound **1**, the following were present: hydrophobic interactions for LEU_135_, LEU_139_, ALA_158_, LEU_115_ and PHE_149_; π–π interactions for TYR_161_ and TYR_138_ with pyrrole cycles; a hydrogen bond between the carboxylic group and ARG_186_. Compounds **2**–**4** were stabilized in their HSA binding sites by similar hydrophobic interactions, as well as by the π–π interaction of TYR_161_ and TYR_138_ with pyrrole cycles and by the interaction of the hydroxyl group of TYR_161_ with Zn^2+^. Furthermore, compound **2** formed a hydrogen bond between the carboxyl group and ARG_117_ in HSA. The PEGDA spacer in compounds **3** and **4** was partially exposed to the solvent. The only interaction observed for compound **4**, with no PEGDA protecting group, was that of the terminal nitrogen with ASP_187_.

**Figure 7:**
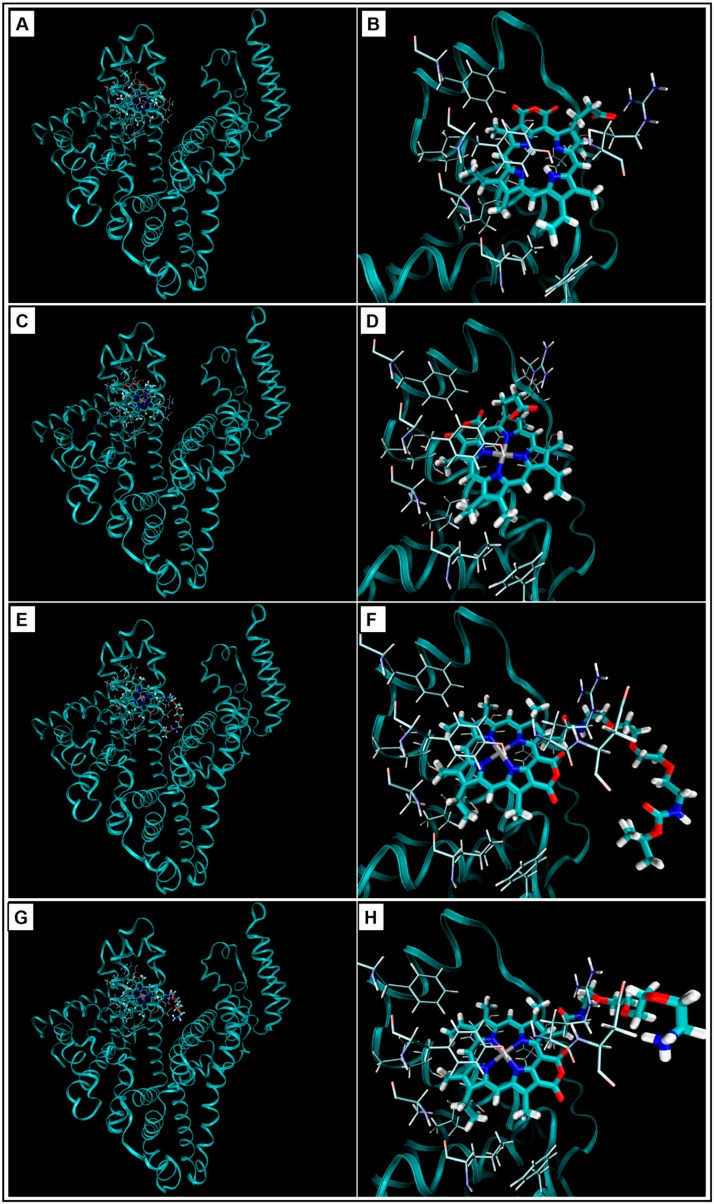
Best positions of docked ligands **1**–**4** shown in licorice representation. Carbon atoms are shown in light blue, hydrogen atoms in white, nitrogen atoms in dark blue, oxygen atoms in red and zinc atoms in grey. Human serum albumin is depicted as a ribbon. The images were captured by VMD software (Theoretical and Computational Biophysics Group, NIH Center for Macromolecular Modeling and Bioinformatics at the Beckman Institute, University of Illinois at Urbana-Champaign, USA, version 1.9.2).

Figure 7 shows the positions of docked compounds **1**–**4** with the lowest binding energies. The ligand-HSA binding energies are summarized in Table 4. The presence of a zinc ion and/or PEGDA spacer affected the binding mode by which the ligands docked to the HSA. Compound **1** docked with the lowest binding energy and ligand **4** with the highest (Table 5). Compound **2** was rotated by ca. 45°, resulting in the polar part of the glutaric acid anhydride being partially docked to the nonpolar part of the HSA binding pocket. This is probably the reason why compound **2** had a higher binding energy to HSA than compound **1**; a similar phenomenon was observed by Akimova et al. [67]. The zinc ion in compounds **2**–**4** interacted with the hydroxyl group of TYR_161_ in HSA; a comparable interaction was described for a complex of HSA with heme and hemin [65,68]. Other authors have also reported the increased binding affinity of HSA to a PS after a metal ion was introduced to the PS [69,70,71,72,73,74]. The PEGDA spacer in compounds **3** and **4** was oriented out of the cavity and led to molecule rotation (Figure 7). In some cases, the scoring function can assign a better score to bigger, but worse, ligands based on the higher number of interactions, but this is not the case of compounds **3** and **4**. In these compounds, despite the porphyrin core being to some extent symmetric, the binding modes with higher binding energies were not as favorable as the binding modes for compounds **1** and **2**.

Since HSA binds a wide range of compounds and, thus, is able to reduce their active concentration in blood plasma, it reduces their bioavailability, thereby leading to decreased activity [75,76,77]. Because the HSA complex with compound **1** exhibited the lowest binding energy, it is less vulnerable to dissociation than compounds **2**–**4**, meaning that a higher free concentration of the novel derivatives and, consequently, higher activity can be expected.

### 2.8. Logarithm of Partition Coefficients

The lipophilicity of xenobiotics affects their binding to blood proteins and plays a key role in molecular discovery [78]. The level of their affinity to these proteins is expressed by the quantitative descriptor of lipophilicity, logP (partition coefficient between *n*-octanol and water): the more lipophilic the compound, the stronger its binding to these proteins [79]. Moreover, compound lipophilicity also has a direct impact on its other pharmacological parameters, such as distribution volume and biological half-life. Because logP is related to the cell uptake level of a compound, logP was calculated for our purpurin 18 derivatives. The data presented in Table 5 show that all compounds had logP of less than 5, which indicates that they should yield a good PDT response [54]. The derivatization of compound **1** with a metal ion and/or a PEG spacer led to lower logP values; the corresponding increase in hydrophilicity is an important factor for the use of such a compound in PDT. Gryshuk et al. described an inverse relationship between the lipophilicity and photosensing efficacy of purpurinimides, compounds structurally related to ours. At the same light doses, the purpurinimide concentrations effective for PDT were 30× lower for hydrophilic derivatives than for hydrophobic ones [58].

The logP results confirm those obtained by our docking study. Probably due to the absence of a PEGDA protecting group, the most hydrophilic compound was **4**, whose complex with HSA also displayed the highest binding energy. Conversely, compound **1** docked to HSA with the lowest binding energy and exhibited the highest logP. The presence of a zinc ion decreased the lipophilicity of compound **2** while correspondingly increasing its binding energy. The PEGDA spacer in compound **3** resulted in it having higher lipophilicity, but a less favorable binding energy, than compound **2**.

Our experimental data showed that compounds **3** and **4** exhibited the highest PDT efficacy, suggesting that the PDT activity of the studied ligands increased as their lipophilicity decreased. However, this is in contrast with Akimova et al. [67], who reported that the increased lipophilicity of structurally related compounds resulted in enhanced PDT. Furthermore, Henderson et al. [80], showing the dependence of PDT activity on lipophilicity for various pyropheophorbides, found that the best PDT outcome was achieved at a logP of between 5.6 and 6.6; at higher and lower values, PDT efficacy decreased. Based on these studies, we assume that the same is valid for our tested compounds; namely, as lipophilicity reduced, logP shifted closer to the optimal.

Indeed, when evaluating the pharmacological properties of compounds, it is necessary to consider the fact that their ability to cross cell membranes and, thus, accumulate in cells is improved with augmented lipophilicity [81]. Although compound **1** theoretically had the highest lipophilicity, it exhibited the lowest binding energy in our docking study with HSA. It seems that its high lipophilicity enhances not only its ability to pass through biological membranes but also its binding affinity to HSA, which, in turn, lowers its active concentration and leads to reduced PDT activity. This corresponds with the results of our in vitro experiments. The docking study also showed that compounds **3** and **4** exhibited higher binding energies than compounds **1** and **2**. From this, we conclude that lower binding energy and higher lipophilicity negatively influenced the active concentrations of the compounds, thereby reducing their PDT efficacy. Other researchers have reached similar conclusions about the significant impact of PS lipophilicity on PDT outcome [80,82,83].

## 3. Materials and Methods

### 3.1. Chemistry

#### 3.1.1. General Methods and Materials

Boc-protected PEG-3 amine was purchased from Iris Biotech GMBH (Marktredwitz, Germany), purpurin 18 (compound **1**) from Frontier Scientific, Inc. (Utah, USA). These chemicals were used as supplied. Other common chemicals were purchased from Sigma-Aldrich (Missouri, USA). NMR spectra were recorded by Agilent 400-MR DDR2 (Varian, Palo Alto, USA) spectrometer (^1^H 400 MHz), solvent CD_3_OD was used as calibrator. Chemical shifts are given in δ (ppm). HRMS spectra were measured by Micro Q-TOF with ESI ionization (Thermo Scientific, Waltham, USA). For thin-layer chromatograms, aluminum TLC sheets for detection in UV light (TLC Silica gel 60 F_254,_ Merck, Darmstadt, Germany) were used. For column chromatography, silica gel (30-60 μm, SiliTech, MP Biomedicals, Eschwege, Germany) was used.

#### 3.1.2. Synthesis of Purpurin Zinc Complex—Compound **2**

{3-[(22S,23S)-17-Ethenyl-12-ethyl-13,18,22,27-tetramethyl-3,5-dioxo-4-oxa-8,24,25,26-tetraazahexacyclo[19.2.1.1^6,9^.1^11,14^.1^16,19^.0^2,7^]heptacosa-1(24),2(7),6(27),9,11(26),12,14,16,18,20-decaen-23-yl-κ^4^*N*^8^,*N*^24^,*N*^25^,*N*^26^]propanoato(2-)}zinc^36^

**Figure 8:**
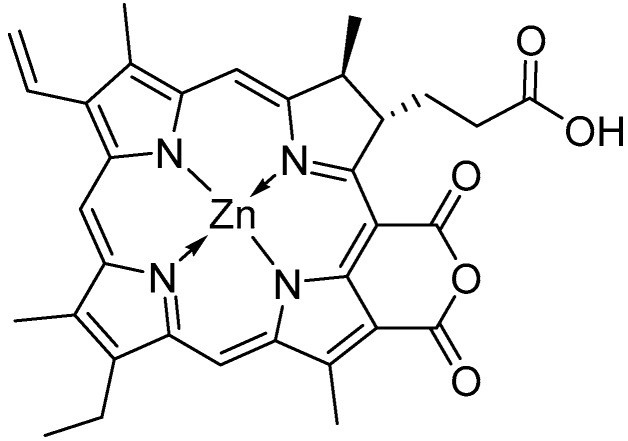
Structure of compound **2**.

To a solution of compound **1** (30 mg, 53 µmol) in chloroform (7 mL), Zn(OAc)_2_·2H_2_O (117 mg, 0.53 mmol; in 3 mL of MeOH) was added. This mixture was stirred for 12 h at 45 °C. The solvents were removed under reduced pressure and the residue was chromatographed (CHCl_3_-MeOH, 40/1). The obtained product was redissolved in a small amount of chloroform and precipitated by the addition of hexanes. Product **2** (20 mg, 32 µmol; Figure 8) was obtained as a dark green solid in 61% yield. After analyses, the product was lyophilized from 1,4-dioxane. R_F_ = 0.7 in DCM-MeOH, 10/1. ^1^H NMR (400 MHz, CD_3_OD) δ: 1.42 (t, *J* = 7.2 Hz, 3 H), 1.77 (d, *J* = 7.0 Hz, 3 H), 1.98–2.08 (m, 1 H), 2.39–2.59 (m, 2 H), 2.72 (dt, *J* = 10.0, 4.9 Hz, 1 H), 2.76–2.82 (m, 2 H), 3.18 (s, 3 H), 4.33 (q, *J* = 7.3 Hz, 1 H), 5.04 (dd, *J* = 9.0, 2.0 Hz, 1 H), 6.01 (dd, *J* = 11.5, 1.0 Hz, 1 H), 6.09 (dd, *J* = 17.8, 1.0 Hz, 1 H), 7.78 (dd, *J* = 17.8, 11.5 Hz, 1 H), 8.38 (s, 1 H), 8.78 (br. s., 1 H), 8.84 (br. s., 1 H); Figure S1 in SI. HRMS-ESI: *monoisotopic mass* 626.15077 Da, found *m/z* 625.14392 [M-H]^−^; Figure S4 in SI.

#### 3.1.3. Synthesis of Purpurin-PEG3-Boc Zinc Complex—Compound **3**

[*Tert*-butyl{15-[(22*S*,23*S*)-17-ethenyl-12-ethyl-13,18,22,27-tetramethyl-3,5-dioxo-4-oxa-8,24,25,26-tetraazahexacyclo[19.2.1.1^6,9^.1^11,14^.1^16,19^.0^2,7^]heptacosa-1(24),2(7),6(27),9,11(26),12,14,16,18,20-decaen-23-yl-κ^4^*N*^8^,*N*^24^,*N*^25^,*N*^26^]-13-oxo-3,6,9-trioxa-12-azapentadecan-1-yl}carbamatato(2-)]zinc

**Figure 9:**
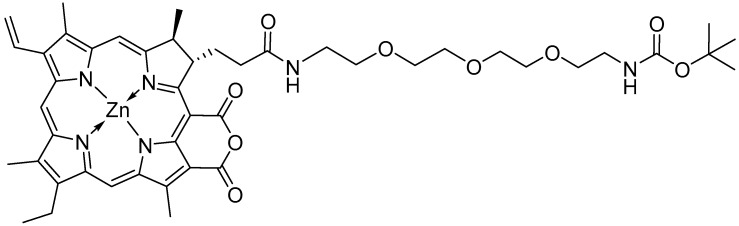
Structure of compound **3**.

To a solution of compound **1** (150 mg, 0.27 mmol) and Boc-PEG_3_-diamine (124 mg, 0.43 mmol) in THF (5 mL), EDIPA (70 mg, 0.54 mmol) and HOBt (37 mg, 0.27 mmol) were added. The mixture was stirred for 5 min, after which DIC (50 mg, 0.4 mmol in 1 mL of THF) was added and the mixture was stirred for 24 h. Then, the solvent was evaporated and the residue was filtered through a short pad of silica (DCM-MeOH, 25/1) to obtain the crude product (250 mg), R_F_ = 0.5 (DCM-MeOH, 20/1). This material was redissolved in chloroform (7 mL) and the solution of Zn(OAc)_2_·2H_2_O (658 mg, 3 mmol) in MeOH (3 mL) was added. The mixture was stirred overnight at 60 °C. Thereafter, the mixture was diluted with chloroform (90 mL) and washed with brine (1 × 100 mL) and water (1 × 100 mL), dried over Na_2_SO_4_, filtered and the solvent was evaporated under reduced pressure. The residue was chromatographed using AcOEt-MeOH, 20/1 as an eluent to obtain compound **3** (99 mg, 0.11 mmol; Figure 9) as a green solid in 41% yield. R_F_ = 0.48 in DCM-MeOH, 10/1. ^1^H NMR (400 MHz, CD_3_OD) δ: 1.25 (t, *J* = 7. 6 Hz, 3 H), 1.37 (s, 8 H), 1.80 (d, *J* = 7.4 Hz, 3 H), 1.94–2.06 (m, 1 H), 2.31–2.43 (m, 2 H), 2.54 (s, 3 H), 2.55–2.62 (m, 1 H), 2.84 (tt, *J* = 14.3, 7.2 Hz, 2 H), 3.09 (q, *J* = 5.5 Hz, 2 H), 3.15 (d, *J* = 2.7 Hz, 5 H), 3.32–3.36 (m, 2 H), 3.40–3.55 (m, 9 H), 4.31 (q, *J* = 7.3 Hz, 1 H), 5.05 (d, *J* = 8.2 Hz, 1 H), 5.96 (dd, *J* = 11.4, 1.6 Hz, 1 H), 6.05 (dd, *J* = 18.0, 1.6 Hz, 1 H), 7.70 (dd, *J* = 17.8, 11.4 Hz, 1 H), 8.01 (t, *J* = 5.3 Hz, N*H*), 8.34 (s, 1 H), 8.44 (s, 1 H), 8.55 (s, 1 H); Figure S2 in SI. HRMS-ESI: *monoisotopic mass* 900.34002 Da, found *m/z* 923.32928 [M+Na]^+^; Figure S5 in SI.

#### 3.1.4. Synthesis of Purpurin-PEG3-Amine Zinc Complex—Compound **4**

[*N*-(2-{2-[2-(2-Aminoethoxy)ethoxy]ethoxy}ethyl)-3-[(22*S*,23*S*)-17-ethenyl-12-ethyl-13,18,22,27-tetramethyl-3,5-dioxo-4-oxa-8,24,25,26-tetraazahexacyclo[19.2.1.1^6,9^.1^11,14^.1^16,19^.0^2,7^]heptacosa-1(24),2(7),6(27),9,11(26),12,14,16,18,20-decaen-23-yl-κ^4^*N*^8^,*N*^24^,*N*^25^,*N*^26^]propanamidato(2-)]zinc

**Figure 10:**
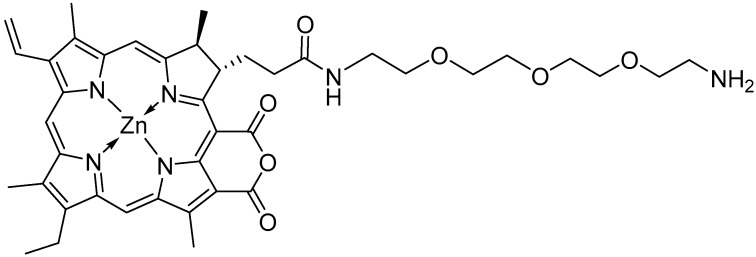
Structure of compound **4**.

Purpurin derivative **3** (99 mg, 0.11 mmol) was dissolved in DCM (5 mL). Five drops of water were added and TFA (1 mL) was added dropwise via a syringe. The mixture was stirred for 1 h, after which it was repetitively evaporated with toluene. The residue was chromatographed (triethylamine-deactivated silica) CHCl_3_-MeOH (30/1→10/1) to obtain product **4** (66 mg, 0.08 mmol; Figure 10) as a dark green solid in 56% yield. R_F_ = 0.2 in DCM-MeOH, 10/1. ^1^H NMR (400 MHz, CD_3_OD) δ: 1.30 (t, *J* = 7.6 Hz, 3 H), 1.76 (d, *J* = 7.4 Hz, 3 H), 1.80–2.09 (m, 4 H), 2.14–2.26 (m, 2 H), 2.28–2.43 (m, 2 H), 2.65 (s, 3 H), 2.70–2.85 (m, 3 H), 2.89–3.03 (m, 3 H), 3.05 (s, 3 H), 3.13 (s, 3 H), 3.15–3.19 (m, 2 H), 3.21–3.25 (m, 2 H), 4.25 (q, *J* = 7.3 Hz, 1 H), 5.07 (dd, *J* = 7, 2.4 Hz, 1 H), 5.95 (dd, *J* = 11.4, 1.2 Hz, 1 H), 6.03 (dd, *J* = 17.8, 1.2 Hz, 1 H), 7.72 (dd, *J* = 17.8, 11.5 Hz, 1 H), 8.31 (s, 1 H), 8.54 (s, 1 H), 8.69 (s, 1 H); Figure S3 in SI. HRMS-ESI: *monoisotopic mass* 800.28759 Da, found *m/z* 801.29590 [M+H]^+^, 823.27637 [M+Na]^+^; Figure S6 in SI.

### 3.2. Indirect Spectrophotometric Measurement of Singlet Oxygen Production

#### 3.2.1. Data Measurement

Singlet oxygen production by compounds **1**–**4** in DMEM + FBS was evaluated using 9,10-anthracenediyl-bis(methylene)dimalonic acid (**AB**, Sigma Aldrich, Saint Loui, MO, USA) as a compound reactive with singlet oxygen. A photosensitizer Rose Bengal (**RB**, 95%, Sigma Aldrich, Saint Loui, MO, USA) was used as a reference. A stock solution of **RB** was prepared in water. Stock solutions of compounds **1**–**4** were prepared by dissolving a solid in DMSO. Working solutions were prepared by diluting the stock solutions with air-saturated solvent which was either phosphate buffered saline (PBS, pH 7.4) or DMEM + FBS. Concentrations of the working solutions of **RB** and compounds **1**–**4** were 1.34 × 10^−5^, 3.19 × 10^−4^, 3.34 × 10^−4^, 2.99 × 10^−4^ and 3.12 × 10^−4^ M, respectively. A stock solution of **AB** was prepared by dissolving solid substance in DMSO (4.87 × 10^−3^ M). Two different amounts of **RB** and compounds **1**–**4** (25 and 50 µL of stock solution) and 15 µL of **AB** stock solution were mixed with 1 mL of solvent in a plastic cuvette (1.000 cm, PMMA, Kartell, Milan, Italy). Two replicates of the same PS concentration were prepared. The first was kept in the dark, the second was illuminated. Absorption spectra of both solutions (illuminated and kept in the dark) were collected against the recorded baseline (Cintra 404, GBC Scientific, 270–800 nm, step 0.2 nm, slit 2 nm) after 0, 10, 20, 30 and 40 min All experiments were done in duplicates.

A 150 W halogen lamp with an edge-pass filter (Panchromar filter (58 mm), VEB Glastechnik Lommatzsch, Lommatzch, Germany) that transmitted light at wavelengths longer than 500 nm was used for illumination of solutions. The fluency rate at the cuvette was 5 mW·cm^−2^. Relative spectral emission intensity of the illuminationg source was measured using spectrofluorometer Fluoromax 2 (Horiba Scientific, Horiba Ltd., Kyoto, Japan). The lamp illuminated a plate made of barium sulfate positioned on front surface accessory of the spectrofluorometer and its spectrum was recorded as reported by Pavlíčková et al. [5]

Emission correction function supplied by manufacturer was applied to the measured spectra. The same correction factor was used to correct spectra of quinine sulfate solution in sulfuric acid and results corresponded well with standard spectra by Velapoldi and Tønnesen [84].

#### 3.2.2. Data Evaluation

Data were evaluated using MS Excel 2010 (Microsoft, Redmond, WA, USA) with a procedure described in detail by Pavlíčková et al. [5] Briefly, from concentrations and a spectrum of a tested compound, its absorbance *A(λ,t)* in solutions was estimated, and then, relative exposure *I*_A_(*t*) for solution illuminated for time *t* was calculated as

$$I_{A}\left(t\right)=\overset{t}{\underset{0}{\int }}\overset{\lambda_{2}}{\underset{\lambda_{1}}{\int }}\frac{I\left(\lambda \right)}{I_{0}}\cdot \left(1-10^{-A\left(\lambda ,\tau \right)}\right)\cdot d\lambda d\tau$$

where $I\left(\lambda \right)/I_{0}$ is source relative emission intensity in photons per wavelength λ and *A*(*λ*,*t*) is absorbance of a tested compound at wavelength *λ* and time *τ* from the beginning of illumination. Wavelength integration limits were *λ*_1_ = 450 nm and *λ*_2_ = 800 nm, and trapezoidal rule for integration was used for absorbance in intervals between spectral measurements. Chemical photodynamical efficiency *γ*_x_ of a photosensitizer was evaluated supposing single exponential dependence of relative **AB** concentration on light exposure

c_rel,AB_ = exp[−γ_x_*I*_A_(*t*)]

Then singlet oxygen quantum yields of photosensitizers were calculated using **RB** in PBS and DMEM+FBS, respectively, as a standard (*ϕ* = 0.75 in PBS, according to Gottfried et al. [40,41]) using equation

*ϕ*_x_ = *ϕ*_s_*γ*_x_/*γ*_s_

where *ϕ*_x_ is estimated quantum yield; *ϕ*_s_ is quantum yield for the standard and *γ*_x_ and *γ*_s_, respectively, are chemical photodynamical efficiencies of an evaluated compound and a standard, respectively.

### 3.3. Biology

#### 3.3.1. Cell Lines and Cultivation Conditions

In our study, we used the following human cell lines: MCF-7 (breast carcinoma), LNCaP (prostate carcinoma, PSMA^+^), PC-3 (prostate carcinoma), U-2 OS (osteosarcoma), MIA PaCa-2 (pancreatic adenocarcinoma), HeLa (cervical carcinoma) and HaCaT (keratinocytes). Unless otherwise specified, the cells were cultured in DMEM medium GlutaMAX (Merck, Kenilworth, NJ, USA) supplemented with 10% FBS (Thermo Fisher Scientific, Waltham, MA, USA). Cells were maintained at exponential phase of growth under standard physiological conditions at 37 °C in humidified atmosphere with 5% CO_2_.

#### 3.3.2. Cell Uptake of Purpurin 18 Derivatives

The number of 1 × 10^5^ cells was seeded on individual 35-mm glass bottom (1.5#) dishes for live-cell imaging (MatTek Corporation, Ashland, MA, USA) and left to adhere for 16 h. Then, the cells were washed with PBS and incubated with compounds **1**–**4** (0.2, 0.5 and 1 µM) dissolved in complete cell cultivation medium without phenol red at 37 °C for 3 and 24 h. After that, the cells were washed twice with PBS and the medium was exchanged for phenol-red free DMEM. Stock solutions of compounds **1**–**4** were prepared in dimethylsulfoxide (DMSO) fresh before the experiments. The final concentration of the vehicle (DMSO) in cell culture medium did not exceed 0.02%.

#### 3.3.3. Determination of Intracellular Localization of Purpurin 18 Derivatives

In order to determine the intracellular localization of compounds **1**–**4**, the cells were seeded and treated with the tested compounds as described in Section 3.3.2. Then, the cells were incubated with a marker for staining of endoplasmic reticulum (ER-TrackerTM Blue-White DPX, 70 nM, 30 min), mitochondria (a green-emitting dimethinium salt from Bříza et al. [47], 70 nM, 10 min and MitoTracker™ Green FM, 70 nM, 15 min), lysosomes (LysoTracker Green DND-26, 70 nM, 15 min) and Golgi apparatus (CellLight™ Golgi-GFP, BacMam 2.0, 24 h, 2 × 10^4^ particles per cell). All the markers used from stock solutions as supplied by the manufacturer (Thermo Fisher Scientific, Waltham, MA, USA).

#### 3.3.4. Fluorescence Microscopy

The intracellular localization of purpurin 18 derivatives was studied by real-time live-cell fluorescence microscopy at 37 °C and in 5% CO_2_ atmosphere. The images were acquired by an inverse fluorescence microscope Olympus IX-81 operated by xCellence System (Olympus, Tokyo, Japan) using a high-stability 150 W xenon arc burner and EM-CCD camera C9100-02 (Hamamatsu, Herrsching am Ammersee, Germany). Living cells were analyzed under physiological conditions (37 °C, 5% CO_2_) by a 60× oil immersion objective (Olympus, Tokyo, Japan) with the numerical aperture of 1.4. All images were deconvolved using xCellence 2D deconvolution module and background-corrected.

#### 3.3.5. Corrected Total Cell Fluorescence

To calculate corrected total cell fluorescence (CTCF), the number of 1 × 10^5^ PC-3 cells was seeded in DMEM+FBS onto glass-bottom MatTek dishes (35 mm, 1.5#). After 16 h, compounds **1**–**4** at 1 µM concentration were added to the cells in DMEM with 10% FBS and incubated for another 24 h. Then, the medium was removed, the cells were washed with PBS, which was replaced with FluoroBrite DMEM media (Thermo Fisher Scientific, Waltham, MA, USA) and subjected to fluorescence microscopy. The fluorescence emission intensity was measured in cells in at least 10 view fields at 600× magnification. The images were taken at the same exposition time (600 ms) and light intensity (100%). Then, the data were evaluated using ImageJ 1.52a software by an equation:

CTCF = Integrated Density − (Area of selected cell × Mean fluorescence of background readings).

#### 3.3.6. Cell Lines and Cultivation Conditions

Photo- and dark toxicity of compounds **1**–**4** was evaluated in vitro by WST-1 viability assay (Sigma, Saint Loui, MO, USA) similarly as in Rimpelová et al. [85]. The WST-1 assay is based on reduction of a tetrazolium salt (WST-1 substrate) into soluble formazan in metabolically active cells. The following cell lines were used: MCF-7, LNCaP, PC-3, U-2 OS, MIA PaCa-2 and HeLa. The cells were seeded into individual wells of 96-well plates (3500 cells per well; except of LNCaP, for which 7000 cells per well was seeded) in 100 µL of DMEM media supplemented with 10% FBS. After 16 h of incubation, the cells were treated with the tested compounds (0.5, 1, 5 and 10 µM) in 100 µL of DMEM with 10% FBS. Then, after 24 h, the cells were washed with PBS, and 100 µL of phenol red-free DMEM was added. One half of the samples was illuminated by 150-W halogen lamp for 13 min with an edge-pass filter (Panchromar, filter (58 mm), VEB Glastechnik Lommatzsch, Lommatzch, Germany) that transmitted wavelengths longer than 500 nm (the total light dose of 4 J·cm^−2^). The second half of the samples was kept in the dark. Next, 24 h after illumination, the cell culture medium was removed and 100 µL of fresh phenol red-free DMEM with 4 µL of WST-1 was added. After 2-h incubation, the absorbance of formed formazan was measured spectrophotometrically at 450 nm (the reference wavelength of 650 nm) using UV–Vis spectrometer (Tecan). The absorbance is directly proportional to the oxidoreductase activity, and thus to the number of metabolically active cells. Cells treated only with cell culture medium and cells treated with a vehicle (DMSO) served as controls. All samples were tested in quadruplicates. The IC_50_ values were determined (GraphPad Prism 6) as the concentration necessary to kill 50% of cells.

#### 3.3.7. Cell Death Evaluation by Flow Cytometry

Evaluation of the proportion of dead cells was adapted from Vermes et al. [86] MCF-7 cells were plated in a 6-well plate (25,000 cells per well) and treated with compounds **1**–**4** (0.1–5 µM) for 24 h. Then, the cells were illuminated for 13 min by a 150-W halogen lamp with an edge-pass filter transmitting wavelengths longer than 500 nm (the total light dose of 4 J·cm^−2^). The cells were incubated at standard cultivation conditions for next 24 h. Afterwards, the cells were harvested by trypsinization, washed in cold PBS and resuspended in annexin-binding buffer followed by labeling with Alexa Fluor^®^ 488 Annexin V and propidium iodide according to the manufacturer’s protocol (Dead Cell Apoptosis Kit, Thermo Fisher Scientific, Waltham, MA, USA) as described in Kirakci et al. [87] and Rumlová et al. [88]. The stained cells were then analyzed by flow cytometer BD FACSAria III, by which live and dead (necrotic and apoptotic) cells were determined using BD FACSDiva 8. The experiments were done in triplicates.

### 3.4. Theoretical Studies

#### 3.4.1. Docking Into Human Serum Albumin

Three-dimensional structures of compounds **1**–**4** (ligands) were created by CORINA Classic (v. 4.2.0; Molecular Networks GmbH, Nuremberg, Germany) software. Other modifications of ligands and proteins were done by Maestro (v. 2018-4, NY, USA) software. To minimize the ligand energy and addition of missing hydrogen atoms, a function “ligand preparation” with the force field OPLS3e was used. The structure of HSA protein with the code of 1N5U was obtained from Protein Data Bank (PDB; https://www.rcsb.org/structure/1N5U) database. From the structure of HAS 1N5U, molecules of myristic acid, protoporphyrin IX and water were removed. In order to define the binding pocket, we chose four amino acids (TYR_161_, MET_123_, HIS_146_, LYS_190_) present in the binding pocket of HSA and using the function “receptor grid generator”, a small and big box was created with the edge length depending on the docked ligand (Table 6). The molecule center was not enabled to leave the small box and the molecule as whole could not overhang the big box. In addition to minimization and optimization of the HSA structure, all amino acids present in this protein were assigned a protonation state corresponding to pH 7 using a function PROPKA. OPLS3e was used as force field. Ligand docking was done in the “extra precisions” mode.

#### 3.4.2. Calculation of the Logarithm of a Partition Coefficient

For compounds **1**–**4**, logP between *n*-octanol and water was calculated as the arithmetic average of results obtained by algorithms XLOGP3, WLOGP, MLOGP, SILICO-IT in SwissADME software (Molecular Modeling Group, Swiss Institute of Bioinformatics, Lausanne, Switzerland) [89,90].

## 4. Conclusions

We have designed, synthesized and presented the biological activity of two PEGylated derivatives of purpurin 18 (compound **1**). Compared to their parent compound, both derivatives (compounds **3** and **4**) exhibited improved accumulation in all of the tested cell lines (PC-3, LNCaP, MCF-7 and HaCaT). Live-cell fluorescence microscopy showed that compounds **3** and **4** localized predominantly in the endoplasmic reticulum and mitochondria, which are desired targets for PDT; moreover, compound **4** also localized in lysosomes. Upon illumination, both compounds efficiently generated singlet oxygen production. This corresponded with their good photodynamic activity at nanomolar to micromolar concentrations in all tested cell lines, with the strongest effect detected for compounds **3** and **4** in the LNCaP and HeLa cell lines, respectively. At submicromolar concentrations, the photoactivated compounds **1**–**4** prevalently induced apoptosis with negligible necrosis. The most efficient apoptosis inducers (61% to 68% of cells in apoptosis) were compounds **3** and **4**. In terms of photodynamic therapy, we believe that our PEGylated derivatives have the ability to outperform their parent photosensitizer purpurin 18, application of which is limited by its aggregation. Furthermore, their enhanced water solubility can overcome the high hydrophobicity and, thus, limited bioavailability associated with photosensitizers, such as chlorin, currently used in photodynamic therapy.

## Figures and Tables

**Table 1:** Estimated chemical photodynamic efficiencies *γ* and singlet oxygen quantum yields *ϕ* for compounds **1**–**4**. Values were measured in phosphate buffered saline (PBS) (except *ϕ*_s_ = 0.75 of **RB** in PBS, as reported by Gottfried et al. [40]) and cell culture media supplemented with 10% fetal bovine serum (DMEM+FBS). Standard deviations of all calculated values were less than 10%.

Compound	Solvent	γ × 10^4^	ϕ
RB	PBS	16.5	0.75 ^1^
	DMEM+FBS	2.68	0.122
1	DMEM+FBS	0.34	0.015
2	DMEM+FBS	1.23	0.056
3	DMEM+FBS	0.63	0.029
4	DMEM+FBS	0.81	0.037

^1^ Reference value according to Gottfried et al. [40].

**Table 2:** Corrected total cell fluorescence (CTCF) of compounds **1**–**4** (1 µM, 24 h) localized in PC-3 cells (see Figure S16 for raw data).

Compound	CTCF × 10^3^
1	1.404 ± 0.134
2	1.593 ± 0.208
3	5.042 ± 0.263
4	6.643 ± 0.405

**Table 3:** Photo- and dark toxicity of compounds **1**–**4** in human cancer cell lines in vitro 24 h after photoactivation (48 h after compound treatment).

IC50 (µM) ^1^								
Compound	1		2		3		4	
Cell Line	Light	Dark	Light	Dark	Light	Dark	Light	Dark
LNCAP	0.34 ± 0.02	>10	0.47 ± 0.03	>10	0.04 ± 0.03	7.20 ± 0.08	0.02 ± 0.00	>10
PC-3	0.16 ± 0.01	>10	0.21 ± 0.01	>10	2.33 ± 0.03	>10	0.65 ± 0.00	>10
U-2OS	1.96 ± 0.01	>10	7.01 ± 0.05	>10	3.17 ± 0.05	>10	1.83 ± 0.01	>10
MIA PACA-2	1.51 ± 0.03	>10	1.04 ± 0.03	>10	1.12 ± 0.01	>10	0.45 ± 0.05	>10
MCF-7	1.62 ± 0.02	>10	2.95 ± 0.01	>10	2.00 ± 0.02	>10	0.59 ± 0.03	>10
HELA	3.40 ± 0.02	>10	>10	>10	0.06 ± 0.05	7.95 ± 0.06	0.02 ± 0.01	>10

^1^ IC_50_—Half maximal inhibitory compound concentration.

**Table 4:** Calculated binding energy of docked ligands (compounds **1**–**4**) with human serum albumin.

Ligands	Calculated Binding Energy (kcal/mol)
1	−13.37
2	−11.58
3	−9.32
4	−8.52

**Table 5:** Calculated logarithm of partition coefficients between *n*-octanol and water (logP) for compounds **1**–**4**.

Compounds	Calculated logP
1	4.55
2	3.78
3	4.05
4	2.88

**Table 6:** Edge size of a box used for ligand docking.

Ligand	Box Edge Size (Å)	
	Small	Big
1	14	22
2	14	22
3	15	30
4	15	28

## Supplementary Materials

The following are available online at https://www.mdpi.com/1420-3049/24/24/4477/s1, Chemical analysis (NMR spectra, HRMS spectra), Biological analysis (compound uptake by human cells, compound colocalization with organelle markers, cell death, photo- and cytotoxicity graphs). Figure S1: ^1^H-NMR spectra of compound **2**, Figure S2: ^1^H-NMR spectra of compound **3**, Figure S3-1: ^1^H-NMR spectra of compound **4**, Figure S3-2: ^13^C-NMR spectra of compound **3**, Figure S4: HRMS spectra of compound **2**, Figure S5: HRMS spectra of compound **3**, Figure S6-1: HRMS spectra of compound **4**, Figure S6-2: Absorption, excitation and emission spectra of compounds **3** and **4**, Figure S6-3: Depletion of 9,10-anthracenediyl-bis(methylene)dimalonic acid without presence of photosensitizer-generated singlet oxygen, Figure S6-4: Depletion of 9,10-anthracenediyl-bis(methylene)dimalonic acid with RB-generated singlet oxygen, Figure S7: Fluorescence microscopy images of the intracellular localization of purpurin 18 (compound **1**) and its derivatives (compounds **2–4**), Figure S8: Fluorescence microscopy images of purpurin 18 (compound **1**) and its derivatives (compounds **2–4**) localization in the endoplasmic reticulum of human MCF-7 cells (breast carcinoma), Figure S9: Fluorescence microscopy images of purpurin 18 (compound **1**) and its derivatives (compounds **2–4**) localization in the endoplasmic reticulum of human immortalized keratinocytes (HaCaT cells), Figure S10: Fluorescence microscopy images of purpurin 18 (compound **1**) and its derivatives (compounds **2–4**) localization in the endoplasmic reticulum of human LNCaP cells (prostate carcinoma), Figure S11: Fluorescence microscopy images of purpurin 18 (compound **1**) and its derivatives (compounds **2–4**) localization in the mitochondria of human MCF-7 cells (breast carcinoma), Figure S12: Fluorescence microscopy images of purpurin 18 (compound **1**) and its derivatives (compounds **2–4**) localization in the mitochondria of human LNCaP cells (prostate carcinoma), Figure S13: Fluorescence microscopy images of purpurin 18 (compound **1**) and its derivatives (compounds **2–4**) localization in the mitochondria of human keratinocytes HaCaT, Figure S14: Fluorescence microscopy images of compound **4** localization in the Golgi apparatus in human PC-3 and MCF-7 cells, Figure S15: Fluorescence microscopy images of compound **4** localization in lysosomes of human immortalized keratinocytes (HaCaT cells), Figure S16: Corrected total cell fluorescence (CTCF) of compounds **1–4** (1 µM, 24 h) localized in PC-3 cells, Table S1: Dose-dependent mechanisms of cell death in MCF-7 cells induced by compounds **1–4**, Figure S17: Photo- and dark toxicity of compounds **1–4** in vitro, Figure S18: Photo- and dark toxicity of compounds **1–4** in vitro.

## Abbreviations

AB, 9,10-anthracenediyl-bis(methylene)dimalonic acid; ALA, δ-aminolevulinic acid; cis-PT, cisplatin; Colo-205, human cells from colorectal carcinoma; DIC, *N*,*N*-diisopropylcarbodiimide; DCM, dichloromethane; DMEM, Dulbecco’s Modified Eagle medium; EDIPA, Hünig’s base; FBS, fetal bovine serum; GFP, green fluorescent protein; HaCaT, human keratinocytes; HSA, human serum albumin; Hep G2, human cells from liver carcinoma; HeLa, human cells from cervical carcinoma; HOBt, *N*-hydroxybenzotriazole; HSA, human serum albumin; IC_50_, half maximal inhibitory concentration of a compound; LNCaP, human cells from prostate carcinoma; MCF-7, human cells from breast carcinoma; MIA PaCa-2, human cells from pancreatic carcinoma; NIH 3T3, mouse fibroblasts; PBS, phosphate buffered saline; PC-3, human cells from prostate carcinoma; PDT, photodynamic therapy; PEG, polyethylene glycol; PEGDA, poly(ethylene glycol) diamine; PI, propidium iodide; PS, photosensitizers; RB, Rose Bengal; TFA, trifluoroacetic acid; U-2 OS, human cells from osteosarcoma; VH10, human foreskin fibroblasts.
