# Supplementary material for: PEGylated Purpurin 18 with Improved Solubility: Potent Compounds for Photodynamic Therapy of Cancer
Source: Molecules. 2019 Dec 6;24(24):4477. doi: 10.3390/molecules24244477 (PMC6943672; doi:10.3390/molecules24244477)
Supplement: Supplementary file 1 [file molecules-24-04477-s001.pdf]

# Supplementary information

## PEGylated purpurin 18 with improved solubility: Potent compounds for photodynamic therapy of cancer

Vladimíra Pavlíčková,[1] Silvie Rimpelová,[1*] Michal Jurášek,[2] Kamil Záruba,[3] Jan Fähnrich,[3] Ivana Křížová,[4] Jiří Bejček,[1] Vojtěch Spiwok,[1] Zdeňka Rottnerová,[5] Pavel Drašar[1]* and Tomáš Ruml[1]*

[1]*Department of Biochemistry and Microbiology, University of Chemistry and Technology in Prague, Technická 3, 166 28, Prague 6, Czech Republic*

[2]*Department of Chemistry of Natural Compounds; University of Chemistry and Technology in Prague, Technická 5, 166 28, Prague 6, Czech Republic*

[3]*Department of Analytical Chemistry; University of Chemistry and Technology in Prague, Technická 5, 166 28, Prague 6, Czech Republic*

[4]*Department of Biotechnology; University of Chemistry and Technology in Prague, Technická 5, 166 28, Prague 6, Czech Republic*

[5]*Central laboratories; University of Chemistry and Technology in Prague, Technická 5, 166 28, Prague 6, Czech Republic*

Corresponding authors:

Silvie Rimpelová E-mail: *silvie.rimpelova@vscht.cz*

Pavel Drašar E-mail: *pavel.drasar@vscht.cz*

Tomáš Ruml E-mail: *tomas.ruml@vscht.cz*

## TABLE OF CONTENTS

**1. CHEMICAL ANALYSIS**

1.1. NMR spectra

1.2. HRMS spectra

**2. BIOLOGICAL ANALYSIS**

# 1. CHEMICAL ANALYSIS

## 1.1. NMR spectra

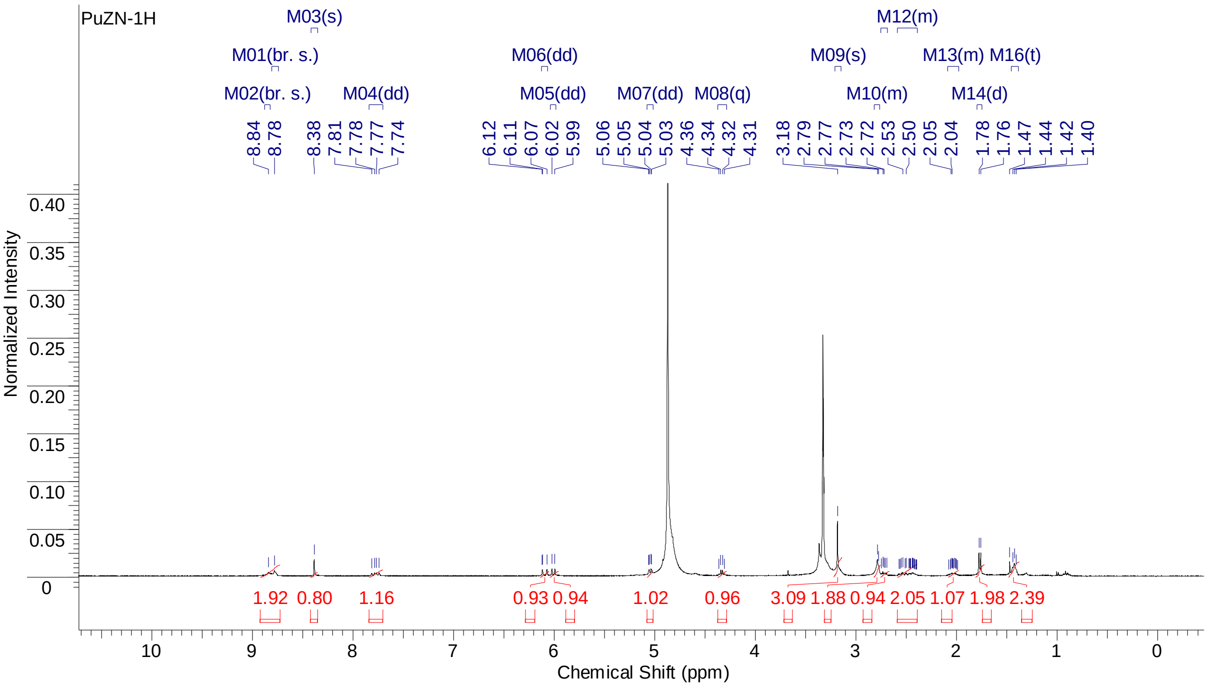


**Figure S1:** $^{1}$H-NMR spectra of compound **2**

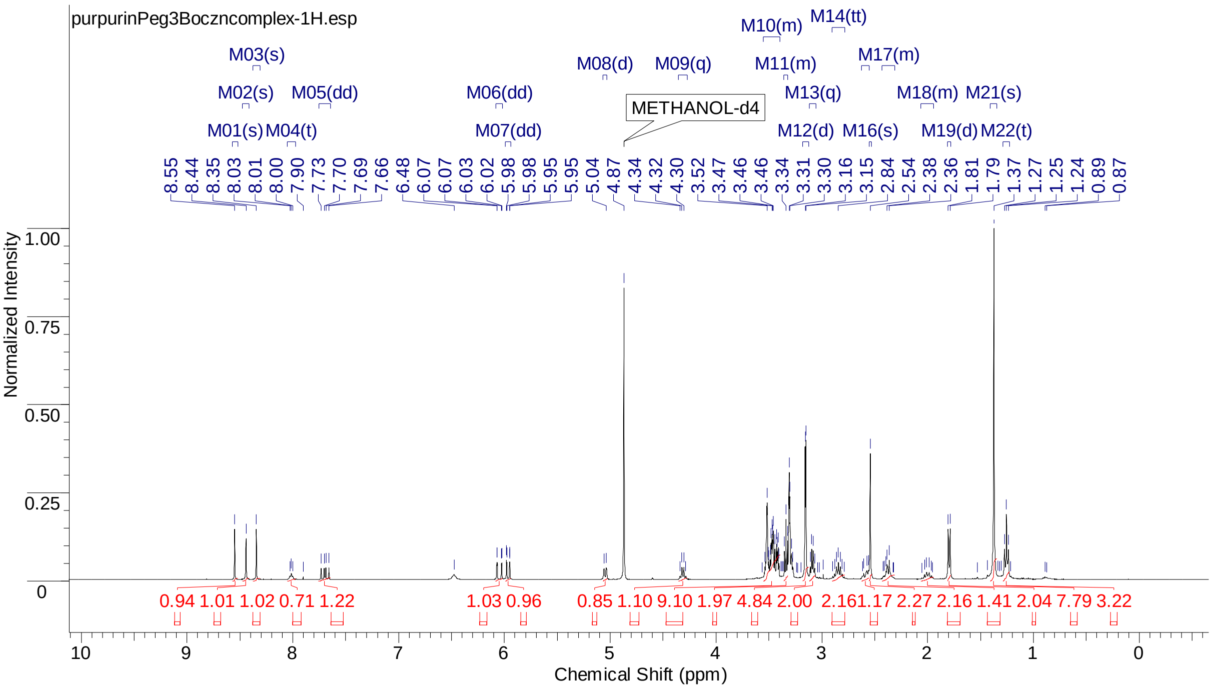


**Figure S2:** $^{1}$H-NMR spectra of compound **3**

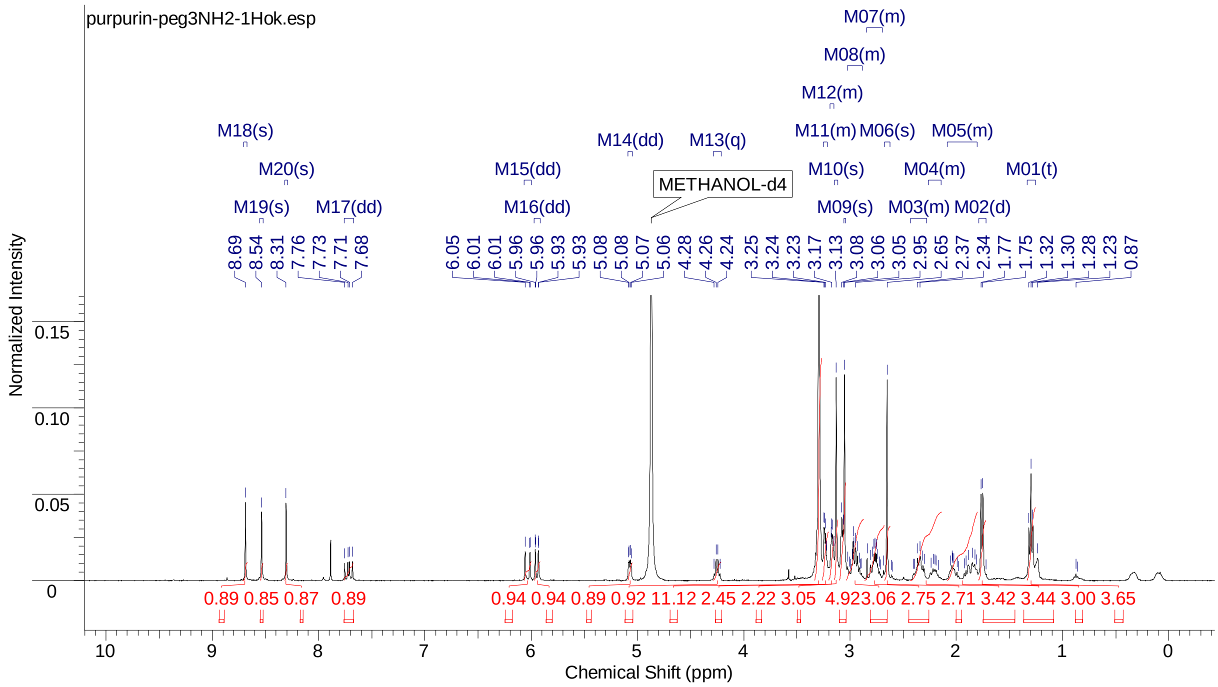

**Figure S3-1:** $^{1}$H-NMR spectra of compound **4**

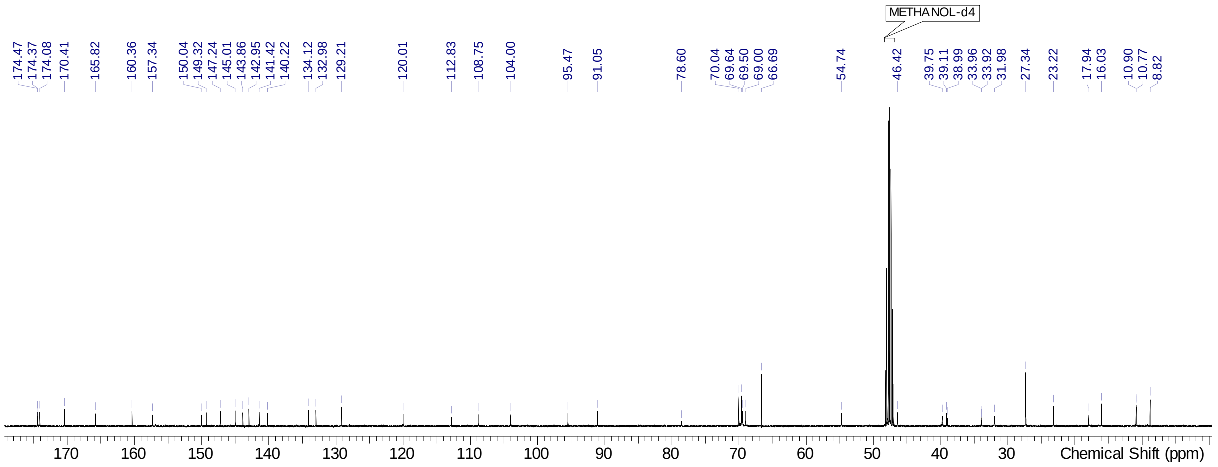

**Figure S3-2:** $^{13}$C-NMR spectra of compound **3**

**$^{13}$C NMR** (101 MHz, $CD_3OD$) δ ppm: 8.82, 10.77, 10.90, 16.03, 17.94, 23.22, 27.34, 31.98, 33.92, 33.96, 38.99, 39.11, 39.75, 46.42, 54.74, 66.69, 69.00, 69.50, 69.64, 69.68, 70.01, 70.04, 78.60, 91.05, 95.47, 104.00, 108.75, 112.83, 120.01, 129.21, 132.98, 134.12, 140.22, 141.42, 142.95, 143.86, 145.01, 147.24, 149.32, 150.04, 157.34, 160.36, 165.82, 170.41, 174.08, 174.37, 174.47.

**$^{13}$C-NMR of compound 4**

$^{13}$C NMR (101 MHz, $CD_3OD$) δ ppm: *Major signals were determined from HSQC:* 8.10, 9.76, 9.78, 15.28, 17.21, 22.11, 28.24, 30.91, 30.92, 32.88, 37.94, 46.96, 45.56, 53.74, 57.89, 65.66, 68.85, 69.00, 69.51, 69.64, 69.90, 94.65, 103.44, 108.61, 119.14, 128.15. The carbons of methyls from Boc group at 27.34 ppm disappeared.

## 1.2.HRMS spectra

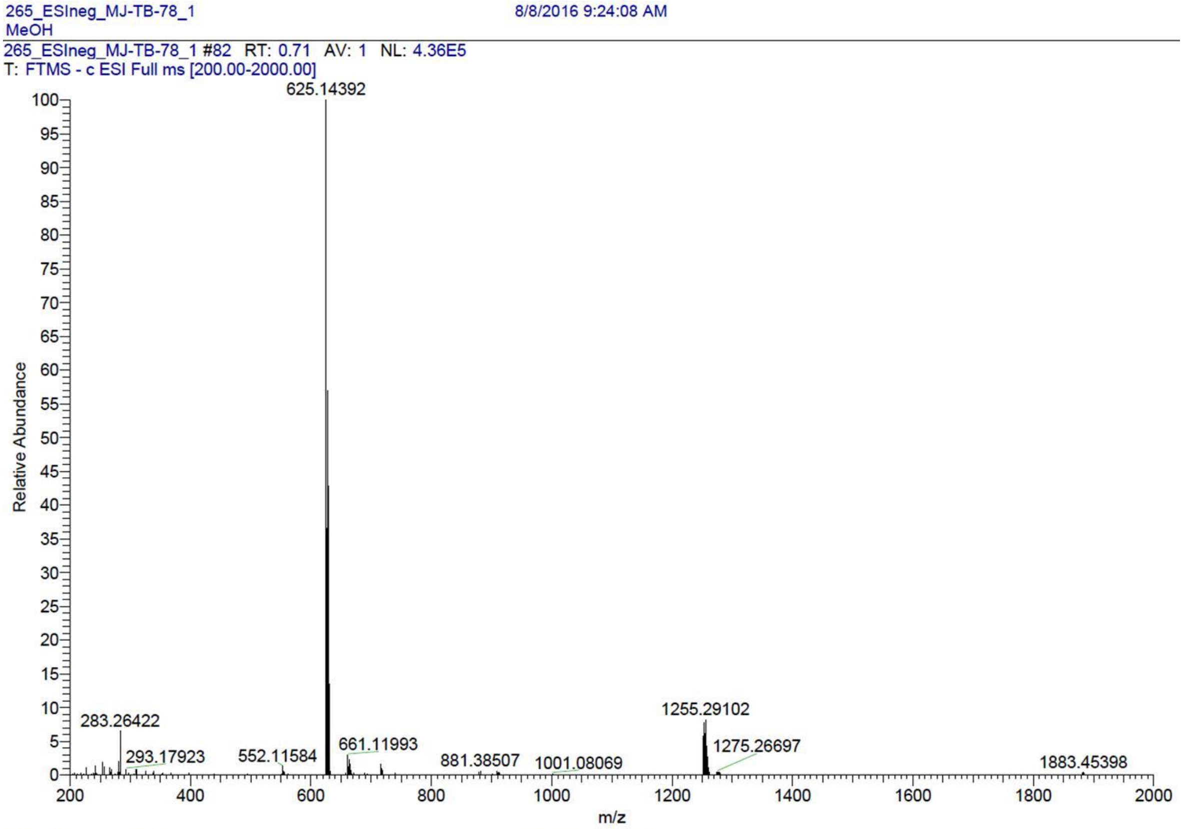


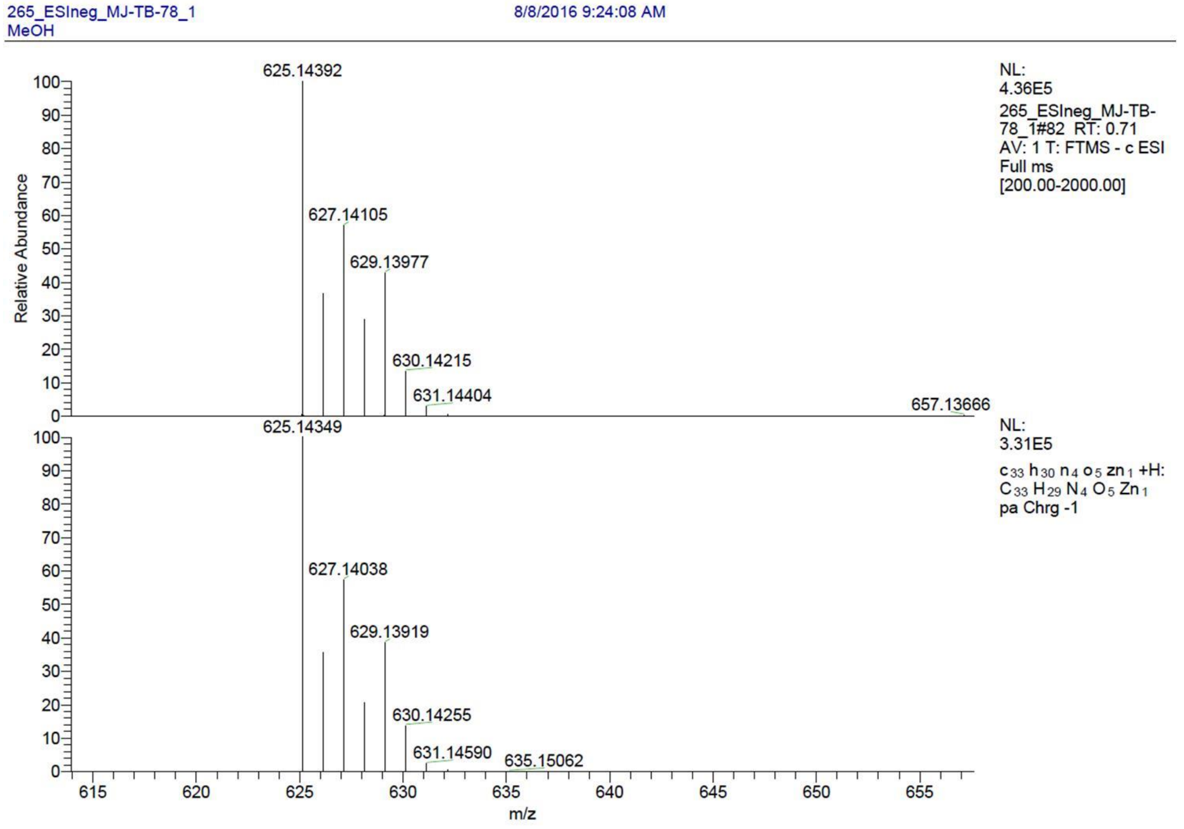


**Figure S4:** HRMS spectra of compound **2**

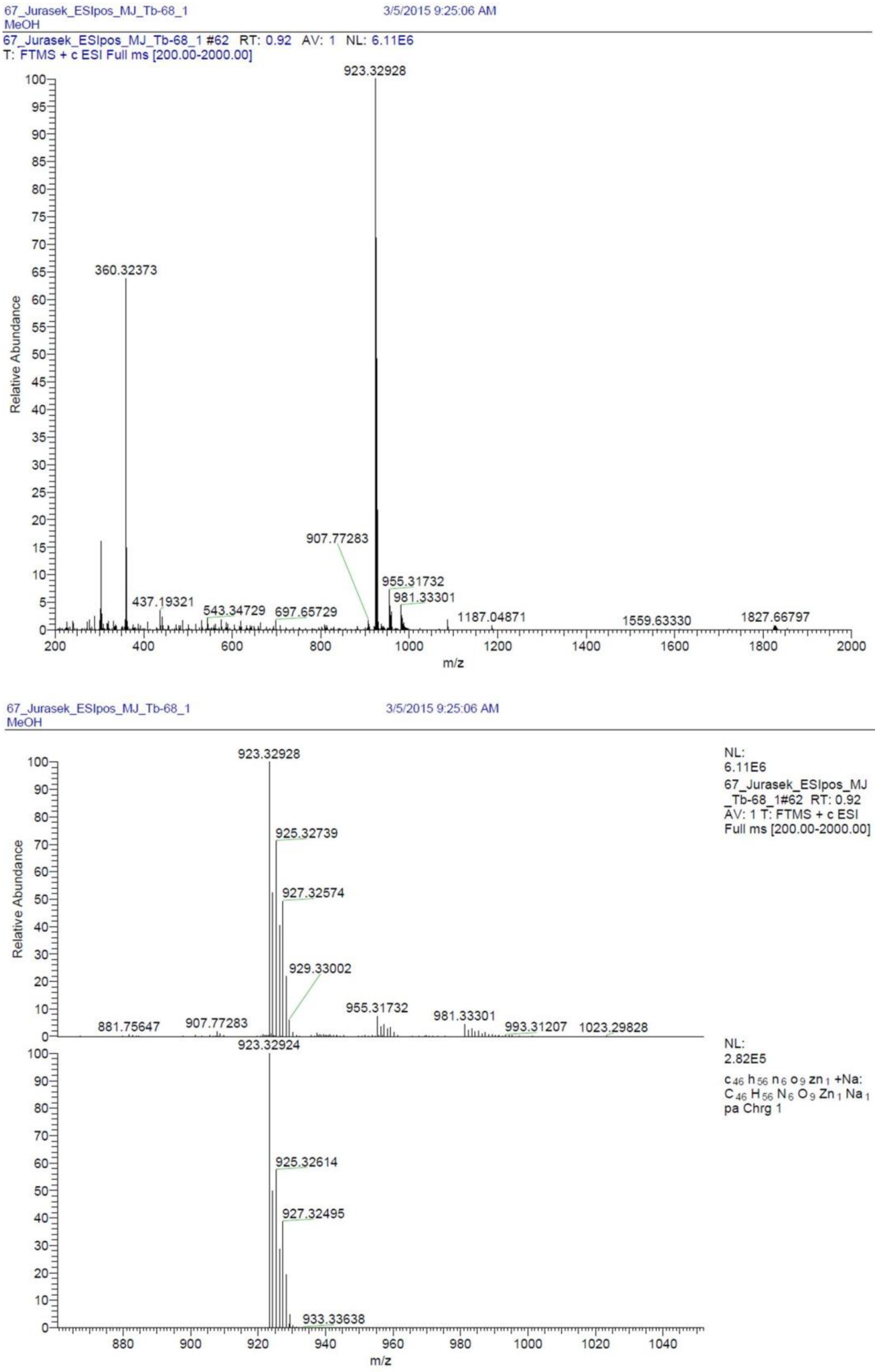


**Figure S5:** HRMS spectra of compound **3**

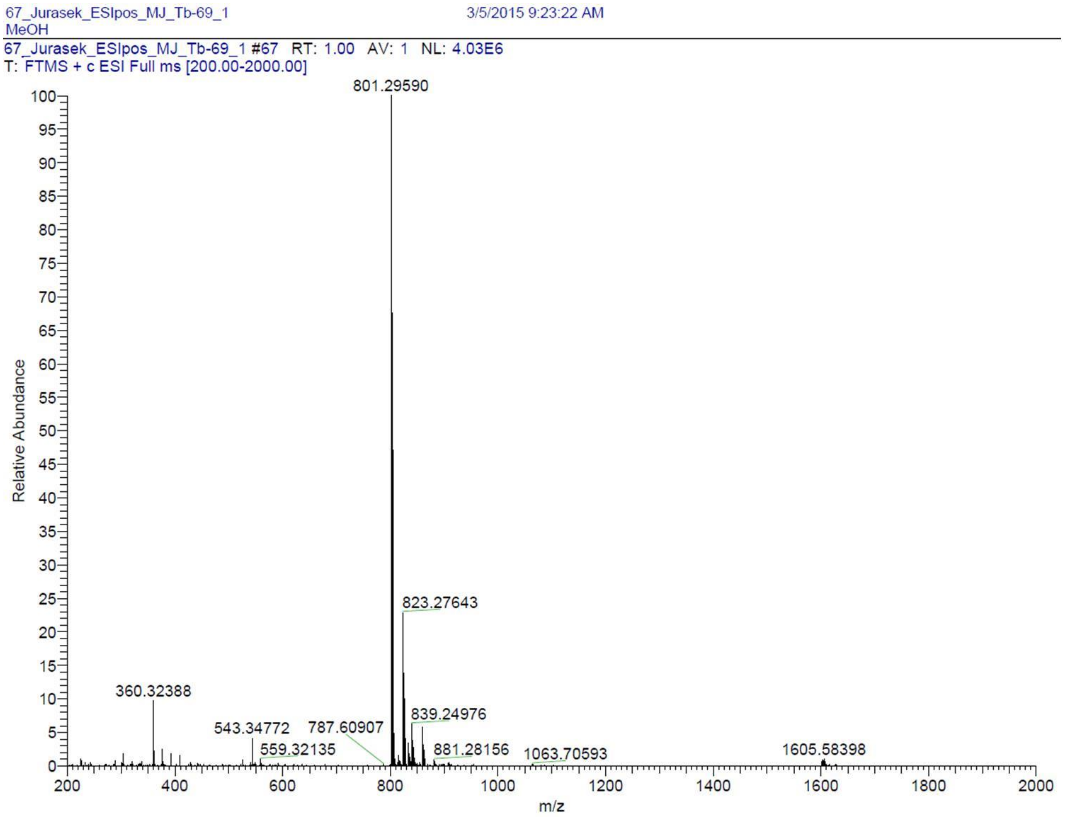


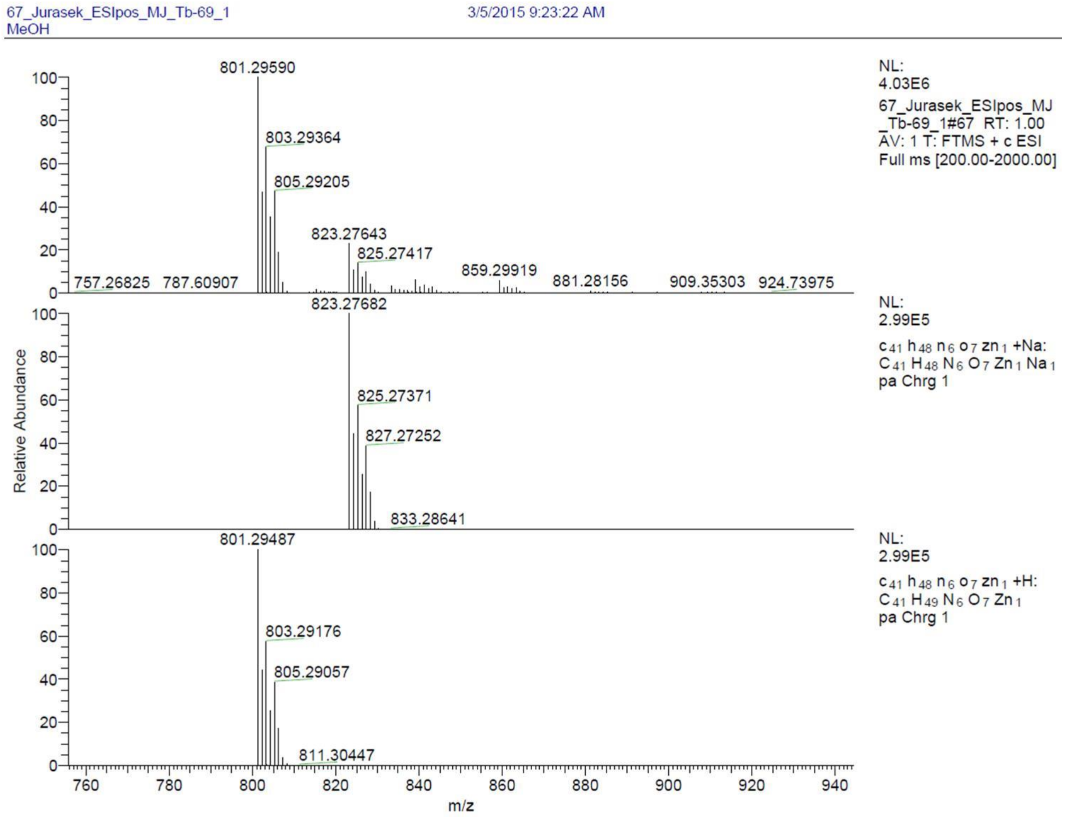


**Figure S6-1:** HRMS spectra of compound **4**

## Spectral properties of compounds 3 and 4

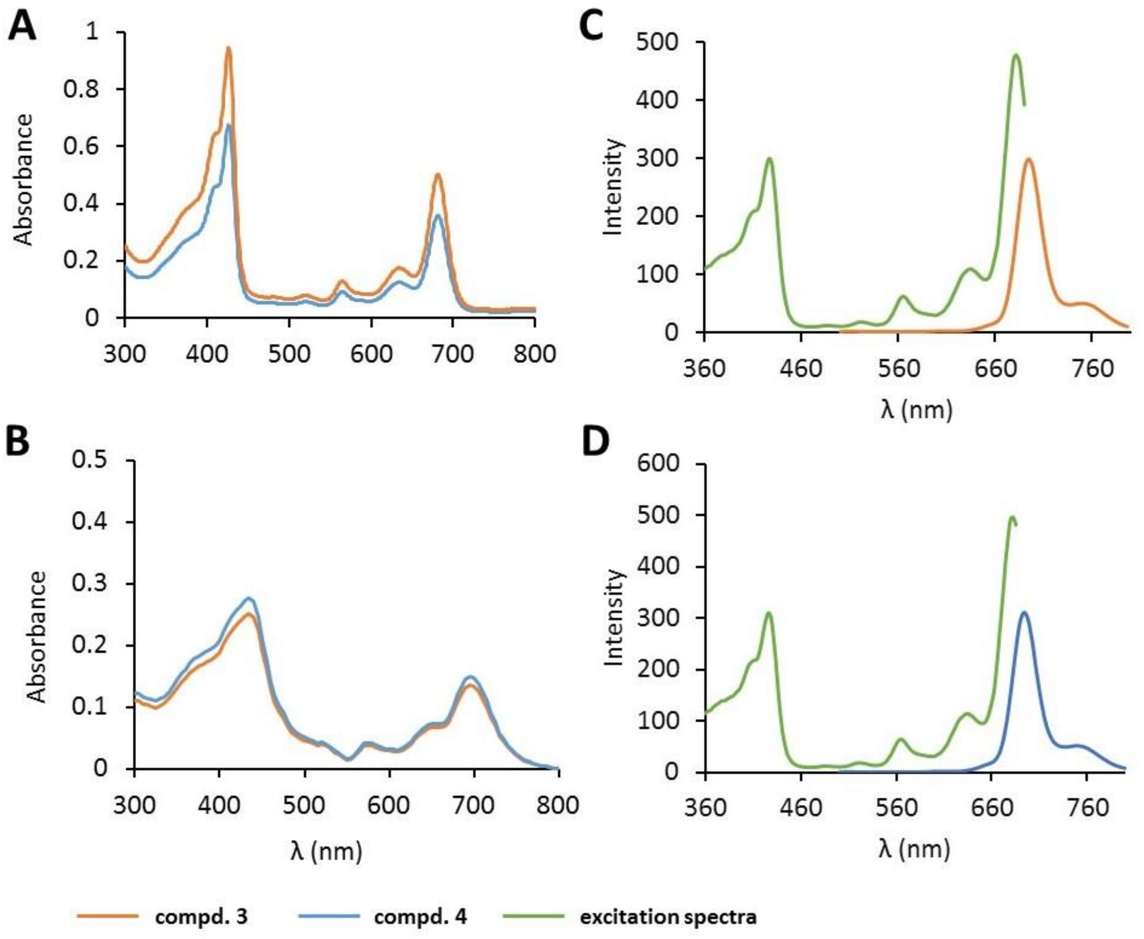


**Figure S6-2:** Absorption, excitation and emission spectra of compounds **3** and **4**. Absorption spectra were measured in **A**) $CHCl_3$-MeOH, 8/2, **B)** phosphate buffered saline (PBS); amber color represents compound **3**, blue color represents compound **4**. **C, D**) Excitation and emission spectra were measured in $CHCl_3$-MeOH, 8/2. Each spectrum was the average of two repeated measurements. The emission and excitation wavelengths were following: **C**) 695 nm (green), 427 nm (amber, compound **3**), **D)** 694 nm (green), 426 nm (blue, compound **4**).

### 1.3. SINGLET OXYGEN GENERATION EFFICIENCY

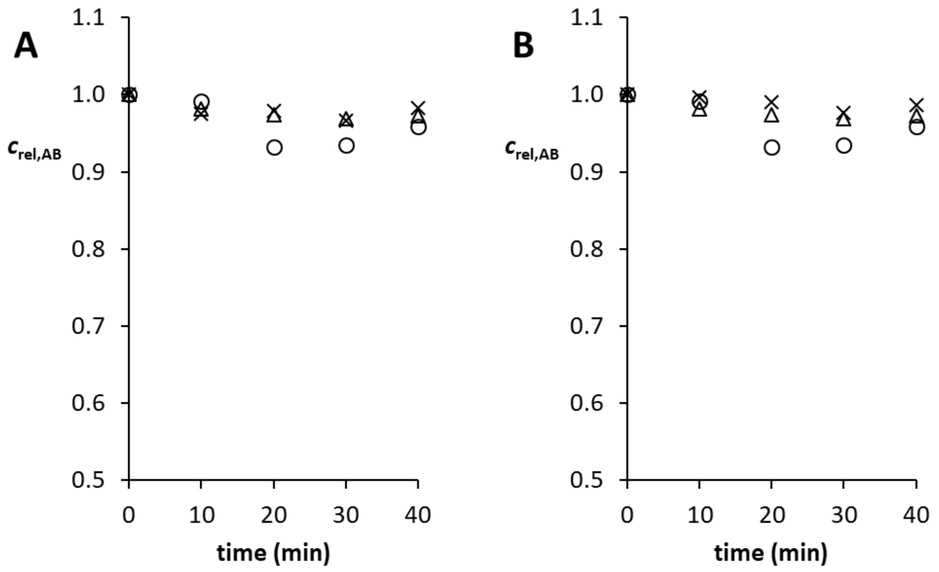


**Figure S6-3:** Depletion of 9,10-anthracenediyl-bis(methylene)dimalonic acid (**AB**, $7\cdot10^{-5}$ M) without presence of photosensitizer-generated singlet oxygen in Dulbecco's Modified Eagle Medium with 10% fetal bovine serum. The experiments were triplicated. (A) solution exposed to light, (B) solution kept in dark. $c_{rel,\mathbf{AB}}$ – relative concentration of **AB** (actual concentration with respect to concentration at experiment start).

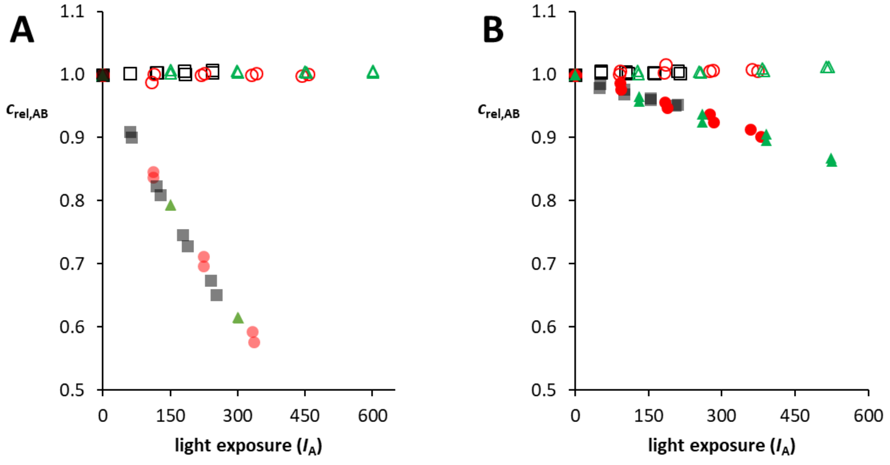


**Figure S6-4:** Depletion of 9,10-anthracenediyl-bis(methylene)dimalonic acid (**AB**, $7\cdot10^{-5}$ M) with **RB**-generated singlet oxygen in (A) PBS (concentrations of **RB** were: $1.3\cdot10^{-7}$ M, $2.6\cdot10^{-7}$ M, $3.1\cdot10^{-7}$ M) and (B) Dulbecco's Modified Eagle Medium with 10% fetal bovine serum (concentrations of **RB** were $2.6\cdot10^{-7}$ M, $5.1\cdot10^{-7}$ M, $7.5\cdot10^{-}$

$^{7}$ M). The experiments were duplicated. ■ ● ▲ – solution exposed to light, □ ○ △ – solution kept in dark. $c_{rel,\mathbf{AB}}$ – relative concentration of **AB** (actual concentration with respect to concentration at experiment start)

## 2. BIOLOGICAL ANALYSIS

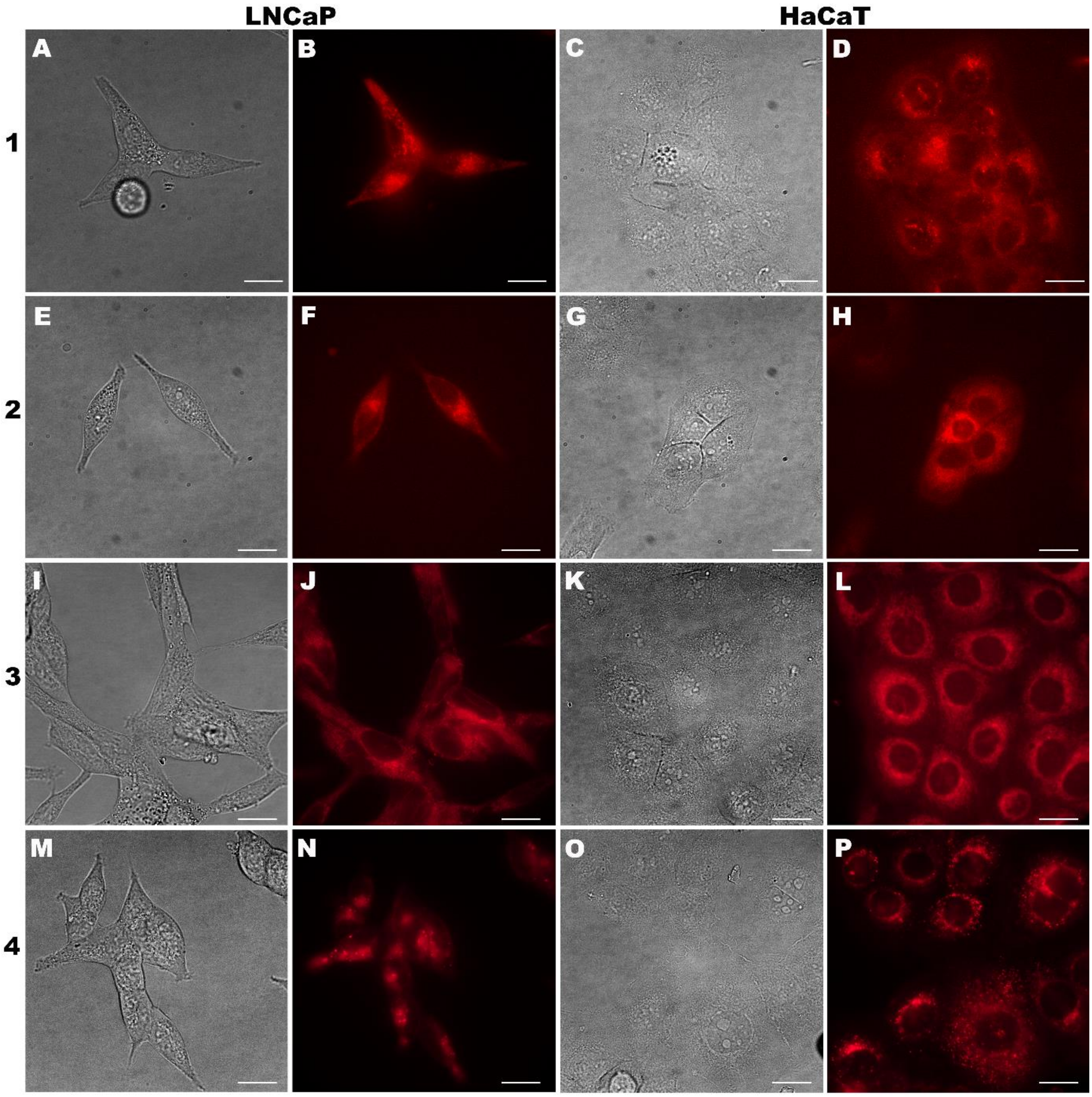


**Figure S7:** Fluorescence microscopy images of the intracellular localization of purpurin 18 (compound **1**) and its derivatives (compounds **2–4**) at 0.5 µM concentration in human cancer cell lines of LNCaP (prostate carcinoma) and HaCaT (keratinocytes) after 24 h incubation. In the first and third column, there are bright field images; in the second and fourth column, there is compound localization. The scale bars represent 20 µm.

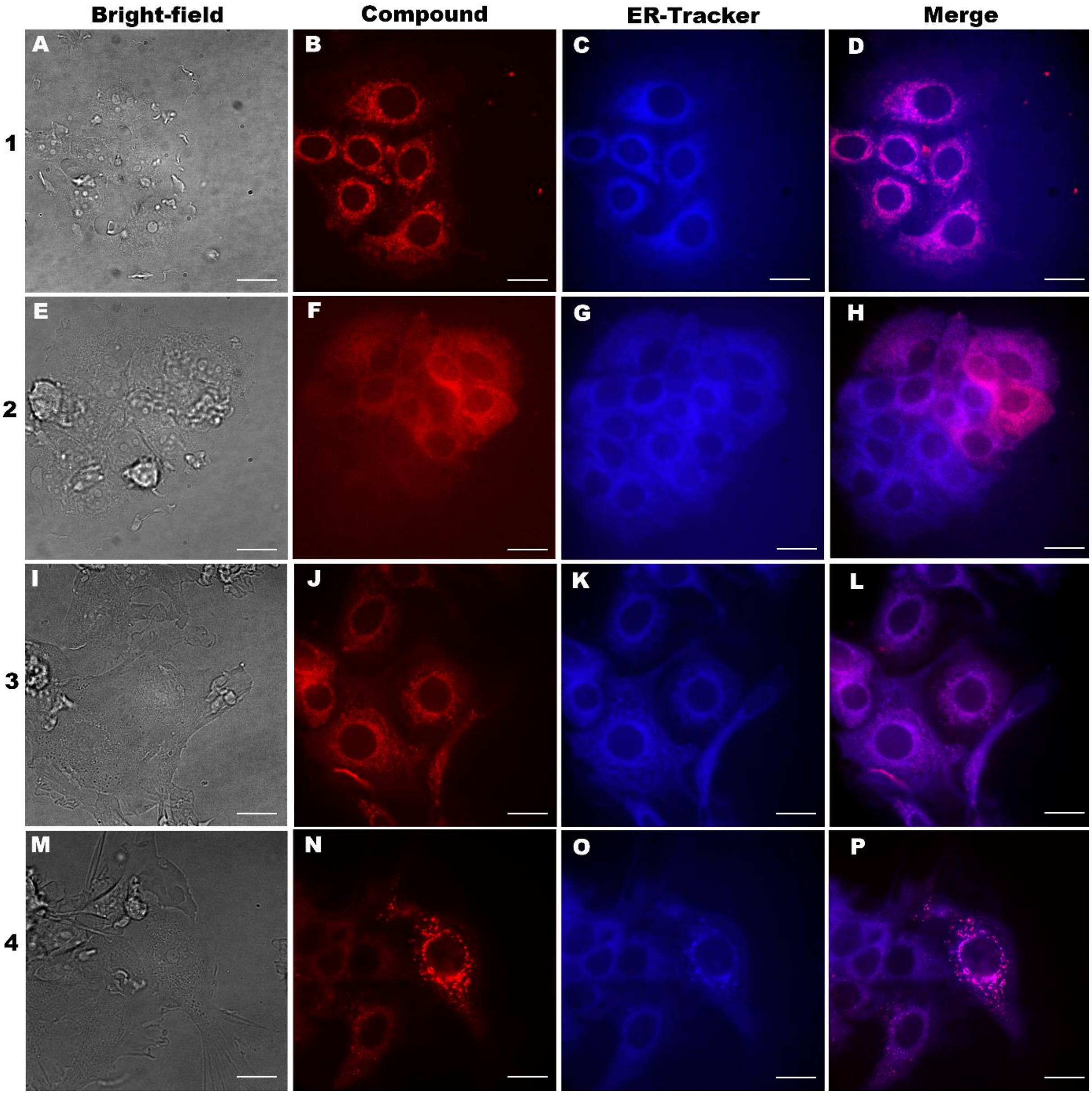


**Figure S8:** Fluorescence microscopy images of purpurin 18 (compound **1**) and its derivatives (compounds **2–4**) localization in the endoplasmic reticulum of human MCF-7 cells (breast carcinoma). Colocalization of compounds **1–2** (0.5 µM, 24 h) or compounds **3–4** (0.5 µM, 24 h) with ER-Tracker™ Blue-White DPX (70 nM, 30 min.). A, E, I, M) Bright-field images; B, F, J, N) localization of the tested compounds; C, G, K, O) ER-Tracker™ Blue-White DPX; D, H, L, P) merge of the fluorescent images. The scale bars represent 20 µm.

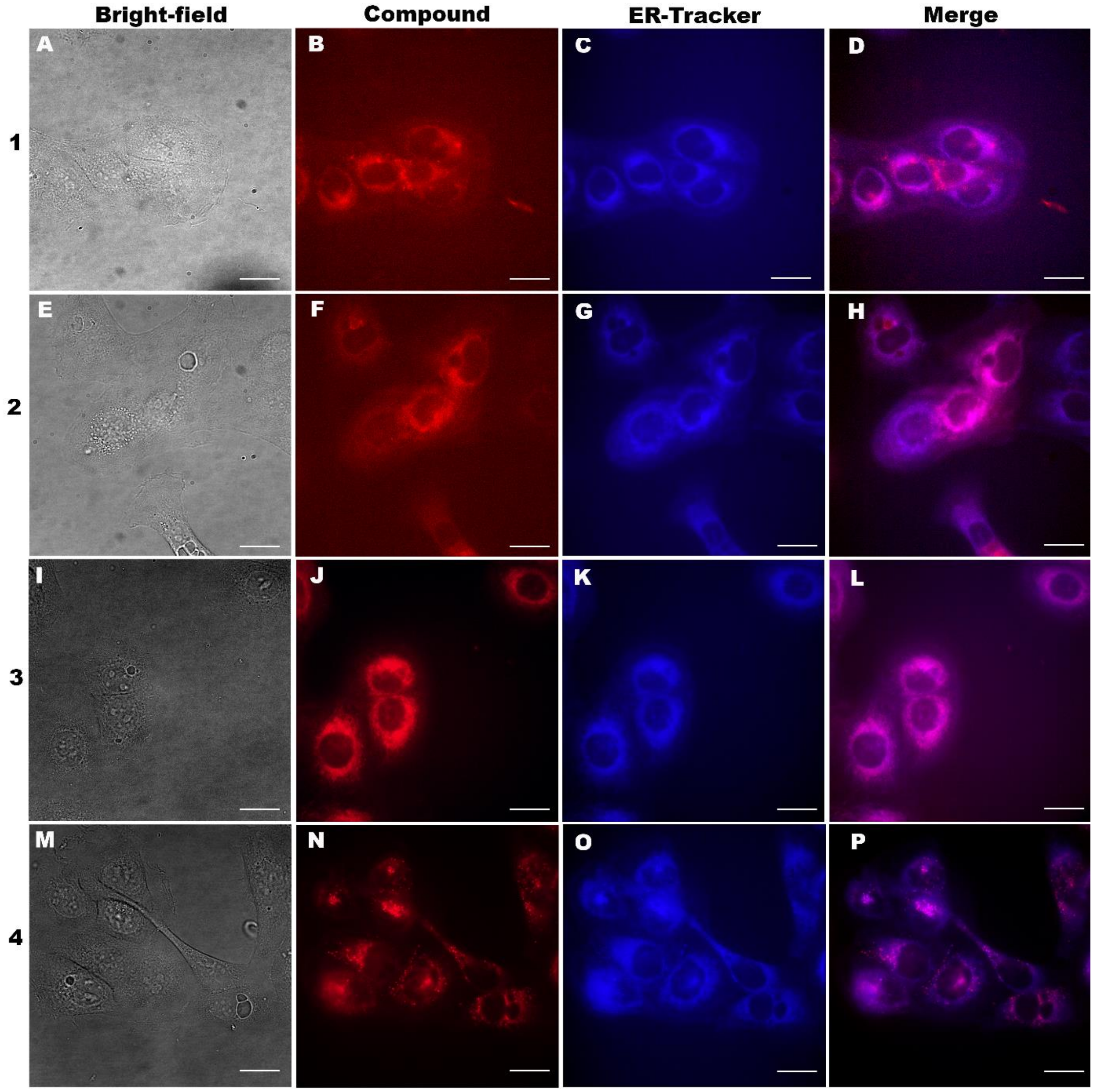


**Figure S9:** Fluorescence microscopy images of purpurin 18 (compound **1**) and its derivatives (compounds **2–4**) localization in the endoplasmic reticulum of human immortalized keratinocytes (HaCaT cells). Colocalization of compounds **1–2** (1 µM, 24 h) or compounds **3–4** (0.5 µM, 24 h) with ER-Tracker™ Blue-White DPX (70 nM, 30 min.). A, E, I, M) Bright-field images; B, F, J, N) localization of the tested compounds; C, G, K, O) ER-Tracker™ Blue-White DPX; D, H, L, P) merge of the fluorescent images. The scale bars represent 20 µm.

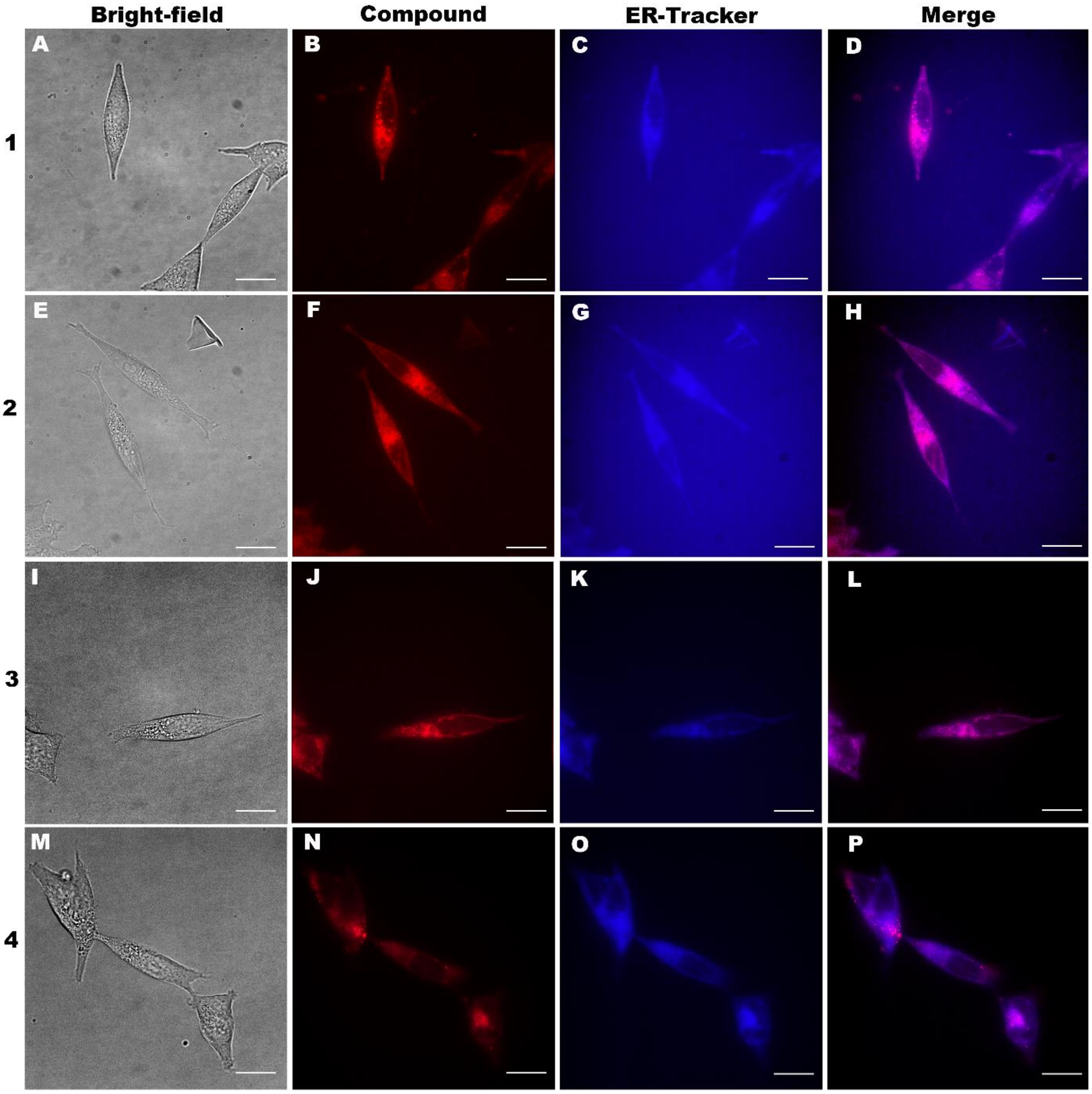


**Figure S10:** Fluorescence microscopy images of purpurin 18 (compound **1**) and its derivatives (compounds **2–4**) localization in the endoplasmic reticulum of human LNCaP cells (prostate carcinoma). Colocalization of compounds **1–2** (0.5 µM, 24 h) or compounds **3–4** (0.5 µM, 3 h) and ER-Tracker™ Blue-White DPX (70 nM, 30 min.). A, E, I, M) Bright-field images; B, F, J, N) localization of the tested compounds; C, G, K, O) ER-Tracker™ Blue-White DPX; D, H, L, P) merge of the fluorescent images. The scale bars represent 20 µm.

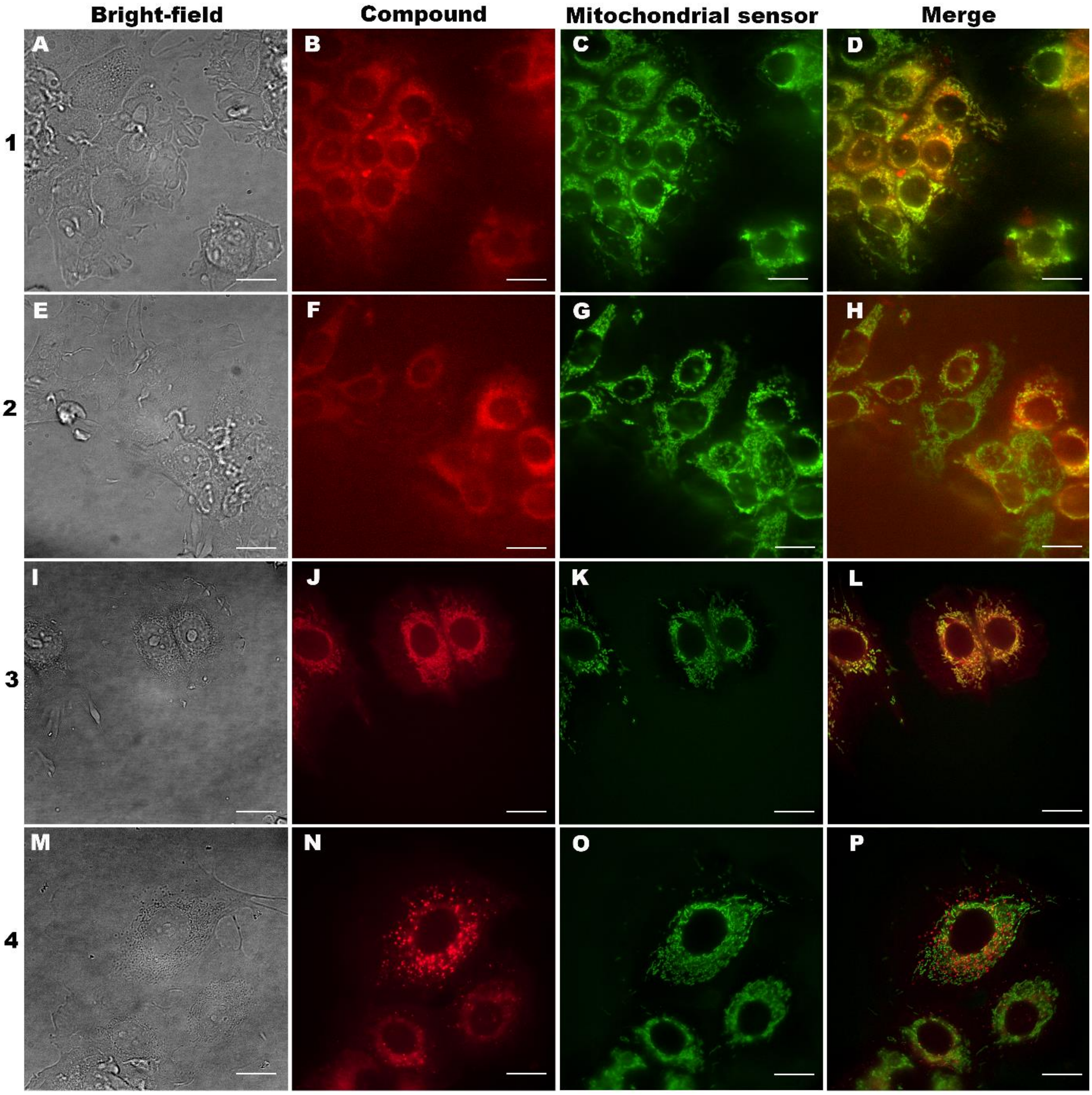


**Figure S11:** Fluorescence microscopy images of purpurin 18 (compound **1**) and its derivatives (compounds **2–4**) localization in the mitochondria of human MCF-7 cells (breast carcinoma). Colocalization of compounds **1–2** (0.5 µM, 24 h) or compounds **3–4** (0.5 µM, 24 h) with a mitosensor (70 nM, 10 min.) based on a dimethinium salt according to Bříza *et al.*[1]. A, E, I, M) Bright-field images; B, F, J, N) localization of the tested compounds; C, G, K, O) mitosensor; D, H, L, P) merge of the fluorescent images. The scale bars represent 20 µm.

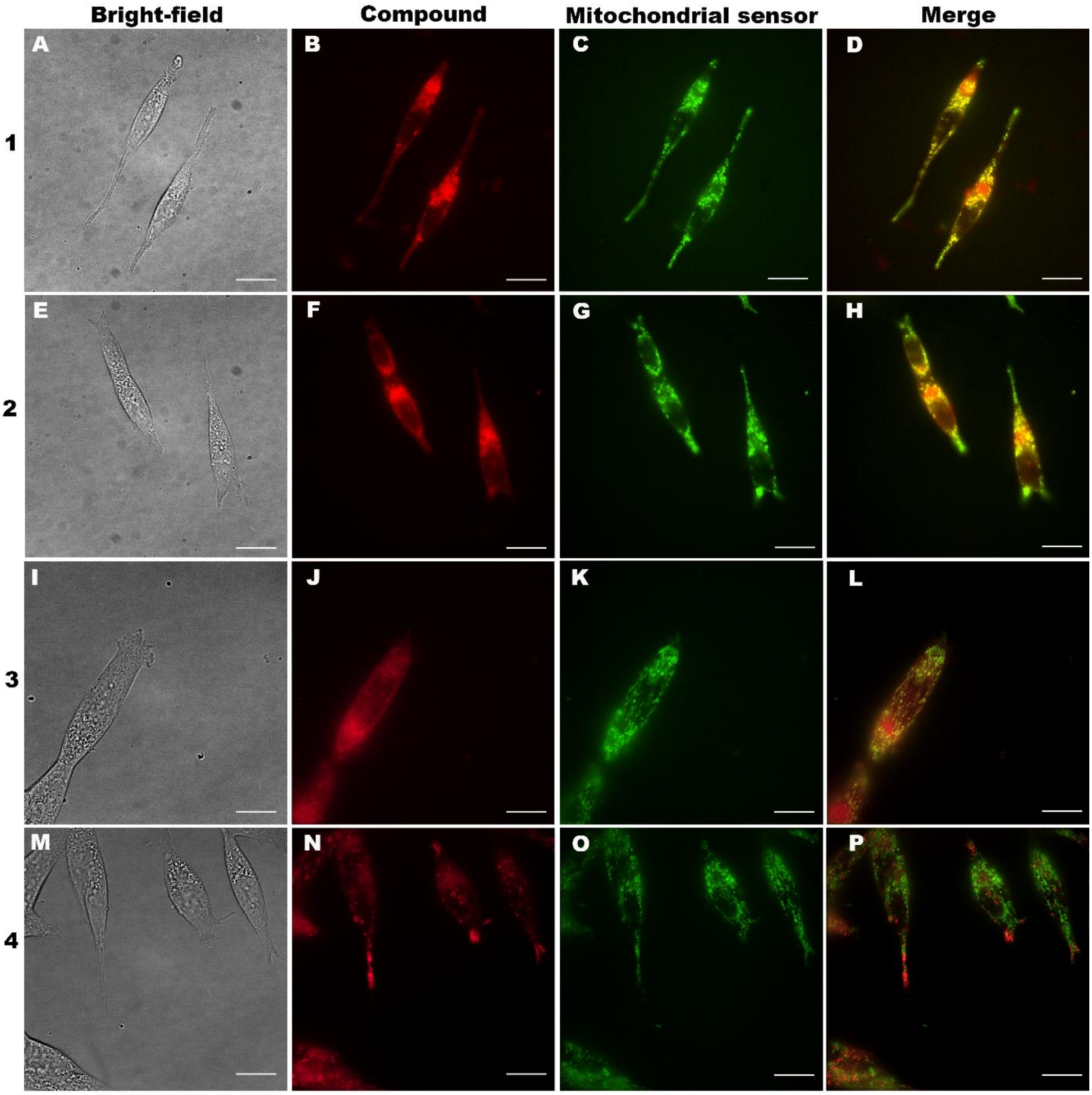


**Figure S12:** Fluorescence microscopy images of purpurin 18 (compound **1**) and its derivatives (compounds **2–4**) localization in the mitochondria of human LNCaP cells (prostate carcinoma). Colocalization of compounds **1-2** (0.5 µM, 24 h) or compounds **3–4** (0.5 µM, 24 h) with MitoTracker™ Green FM (70 nM, 20 min.). A, E, I, M) Bright-field images; B, F, J, N) localization of the tested compounds; C, G, K, O) MitoTracker™ Green FM; D, H, L, P) merge of the fluorescent images. The scale bars represent 20 µm.

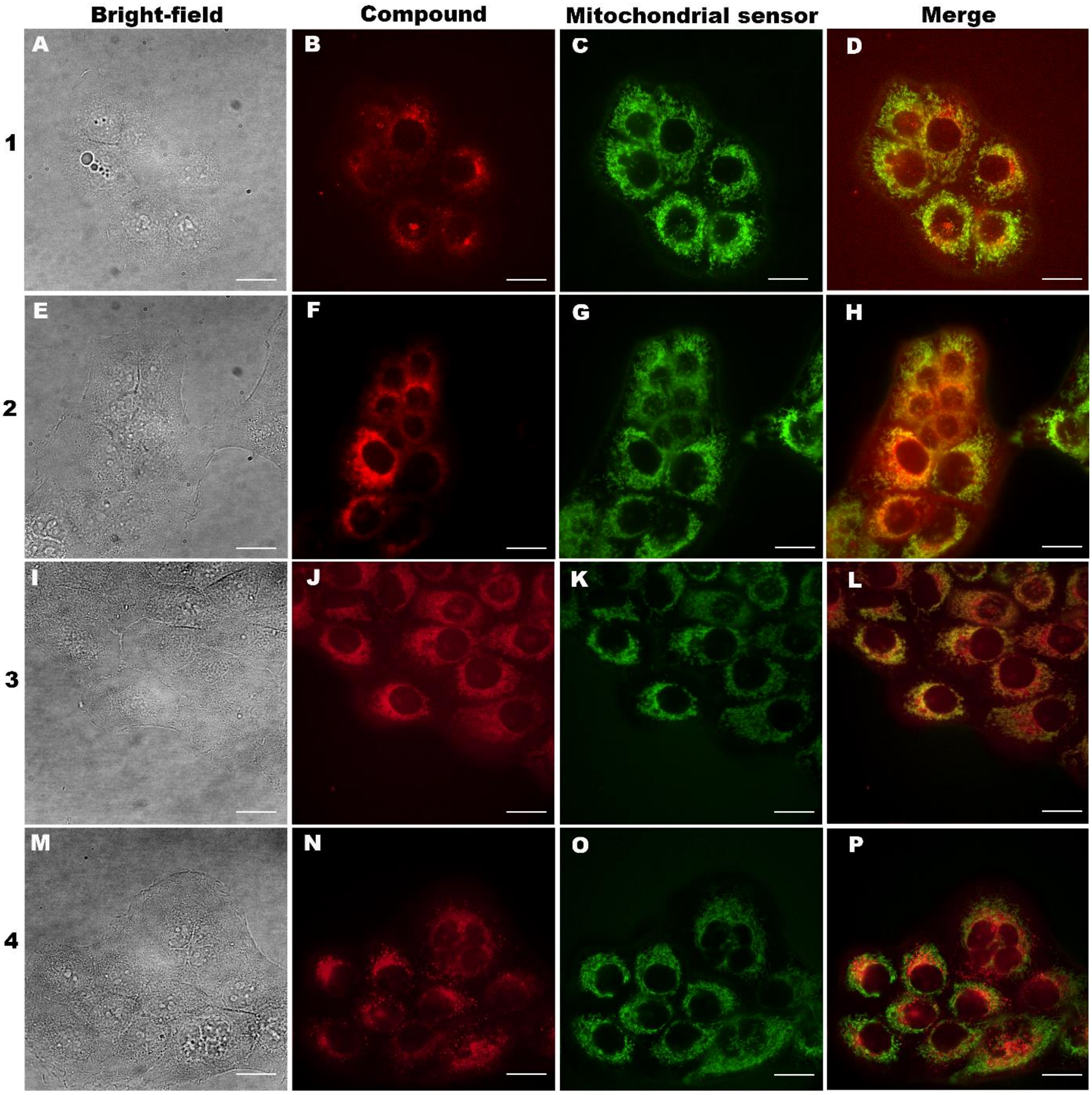


**Figure S13:** Fluorescence microscopy images of purpurin 18 (compound **1**) and its derivatives (compounds **2–4**) localization in the mitochondria of human keratinocytes HaCaT. Colocalization of compounds **1–2** (0.5 μM, 3 h) or compounds **3–4** (0.5 μM, 3 h) and MitoTracker™ Green FM (70 nM, 20 min.). A, E, I, M) Bright-field images; B, F, J, N) localization of the tested compounds; C, G, K, O) MitoTracker™ Green FM; D, H, L, P) merge of the fluorescent images. The scale bars represent 20 μm.

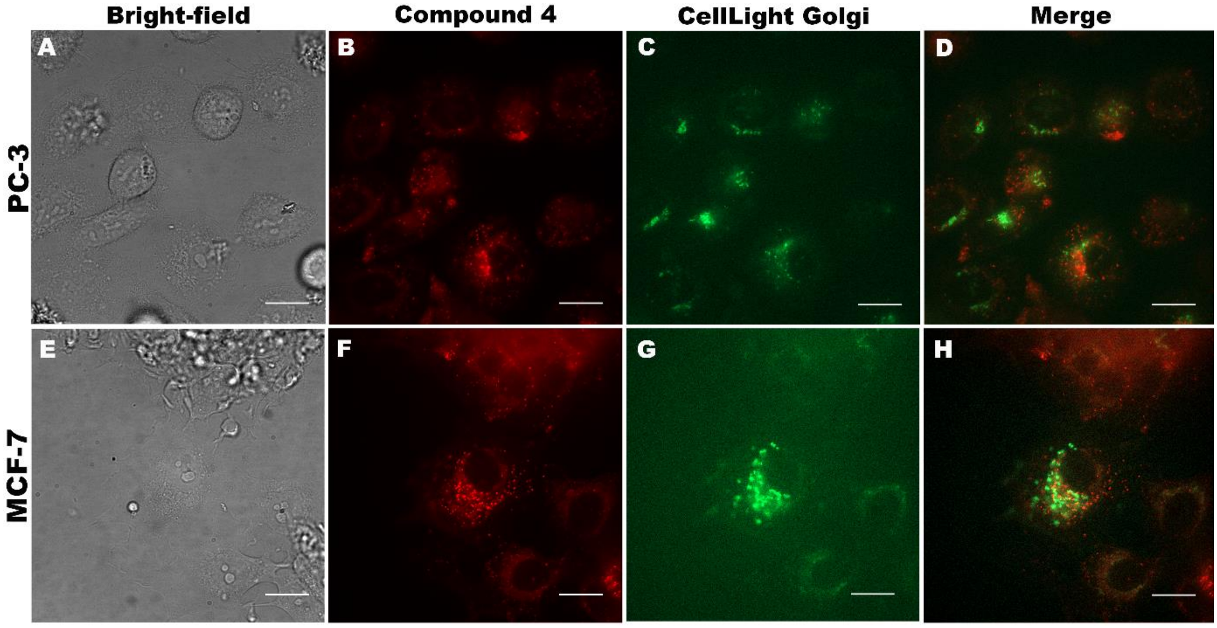


**Figure S14:** Fluorescence microscopy images of compound **4** localization in the Golgi apparatus in human PC-3 and MCF-7 cells. Colocalization of compound **4** (0.5 μM, 24 h) and CellLight Golgi-GFP, BacMam 2.0 ($2{\cdot}10^4$ particles per cell.). A, E) Bright-field images; B, F) localization of compound **4**; C, G) CellLight Golgi-GFP, BacMam 2.0; D, H) merge of the fluorescent images. The scale bars represent 20 μm.

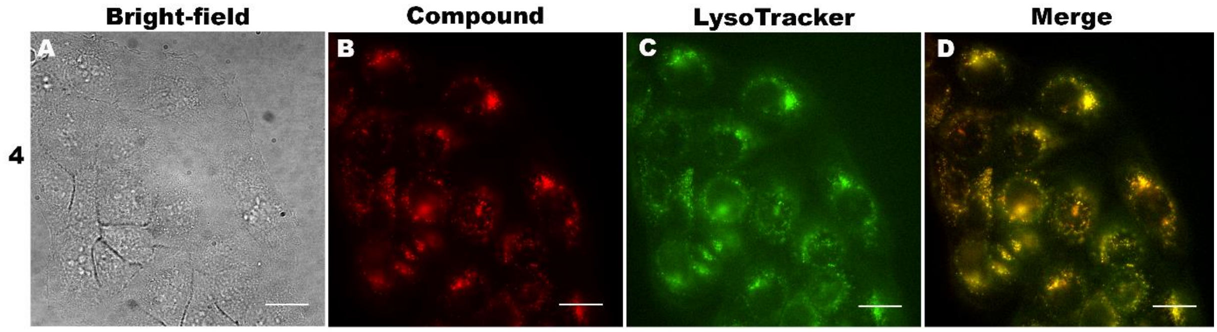


**Figure S15:** Fluorescence microscopy images of compound **4** localization in lysosomes of human immortalized keratinocytes (HaCaT cells). Colocalization of compound **4** (0.5 μM, 24 h) and LysoTracker Green DND-26 (70 nM, 20 min.). A) Bright-field images; B) localization of compound **4**; C) LysoTracker Green DND-26; D) merge of the fluorescent images. The scale bars represent 20 μm.

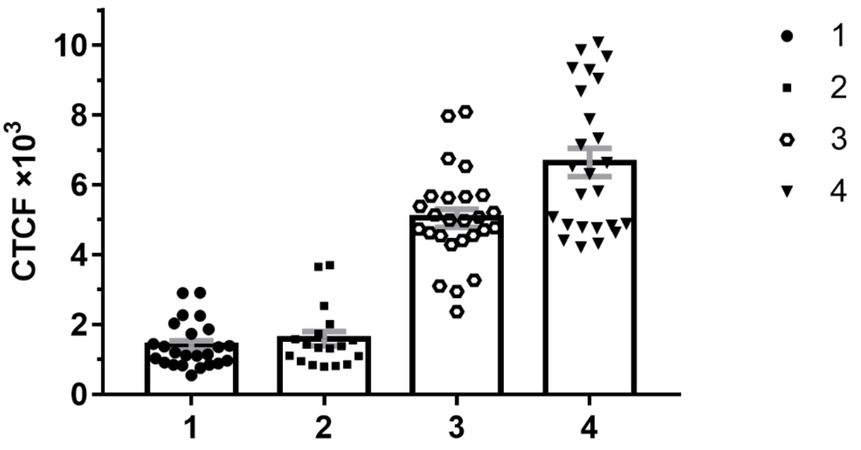


**Figure S16:** Corrected total cell fluorescence (CTCF) of compounds **1–4** (1 µM, 24 h) localized in PC-3 cells

**Table S1:** Dose-dependent mechanisms of cell death in MCF-7 cells induced by compounds **1–4** after 24 h treatment and light induction (Light) measured by flow cytometry. Control represents untreated cells and cells incubated with the same compounds without illumination (Dark). The total light dose was of 4 $J \cdot cm^{-2}$

| | | **Dark** | | **Light** | |
|---|---|---|---|---|---|
| **Compound** | **Concentration [µM]** | **Apoptosis** | **Necrosis** | **Apoptosis** | **Necrosis** |
| **Control** | **0** | 9.5 ± 1.0 | 0 | 10.6 ± 0.6 | 0 |
| **1** | **0.10** | 7.6 ± 1.4 | 0 | 11.0 ± 2.4 | 0.1± 0.1 |
| | **0.25** | 8.5 ± 2.1 | 0 | 14.2 ± 2.7 | 0 |
| | **0.50** | 8.2 ± 1.1 | 0 | 32.6 ± 6.1 | 0.1±0 |
| | **1.00** | 7.3 ± 0.2 | 0 | 51.7± 4.7 | 0.1 ± 0.1 |
| **2** | **0.10** | 10.5 ± 2.3 | 0 | 14.2 ± 2.1 | 0.1 ± 0.1 |
| | **0.25** | 12.1 ± 1.9 | 0.1 ± 0.1 | 18.2 ± 2.6 | 0 |
| | **0.50** | 12.8 ± 3.8 | 0 | 25.8 ± 4.0 | 0.4 ± 0.2 |
| | **1.00** | 14.3 ± 5.1 | 0 | 31.9 ± 4.1 | 0.3 ± 0.3 |
| **3** | **0.10** | 16.3 ± 3.5 | 1.7 ± 1.4 | 36.7 ± 12.1 | 0 |
| | **0.25** | 10.5 ± 0.8 | 0 | 56.1 ± 14.9 | 0 |
| | **0.50** | 13.7 ± 1.5 | 0 | 65.0 ± 9.5 | 0.1 ± 0 |
| | **1.00** | 21.2 ± 7.6 | 0 | 61.1 ± 7.6 | 0.3 ± 0.2 |
| **4** | **0.10** | 14.5 ± 3.7 | 0.1 ± 0.1 | 32.0 ± 2.6 | 0 |
| | **0.25** | 19.8 ± 7.7 | 0 | 49.8 ± 2.9 | 0 |
| | **0.50** | 19.0 ± 7.6 | 0.1 ± 0.1 | 57.8 ± 9.8 | 0 |
| | **1.00** | 22.4± 8.0 | 0.1 ± 0.1 | 68.0 ± 8.4 | 0 |

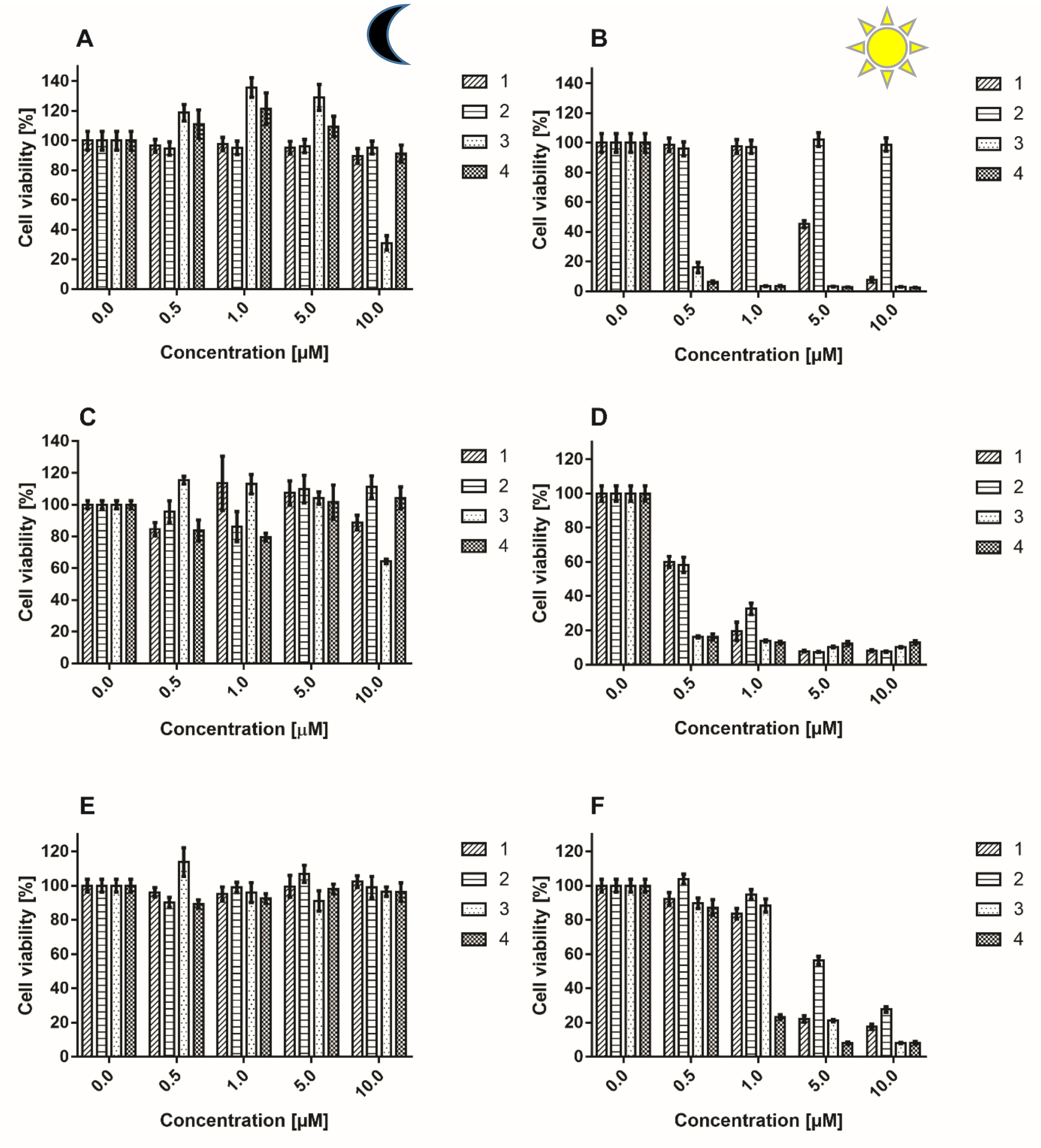


**Figure S17:** Photo- and dark toxicity of compounds **1–4** *in vitro*. Cell viability determined by WST-1 assay after 48 h of incubation with the tested compounds (24 h prior illumination + 24 h after illumination). Left panel dark toxicity (cells kept in the dark after the compound treatment), right panel phototoxicity (cell viability after compound photoactivation). A, B) HeLa, C, D) LNCaP, E, F) MCF-7 cells.

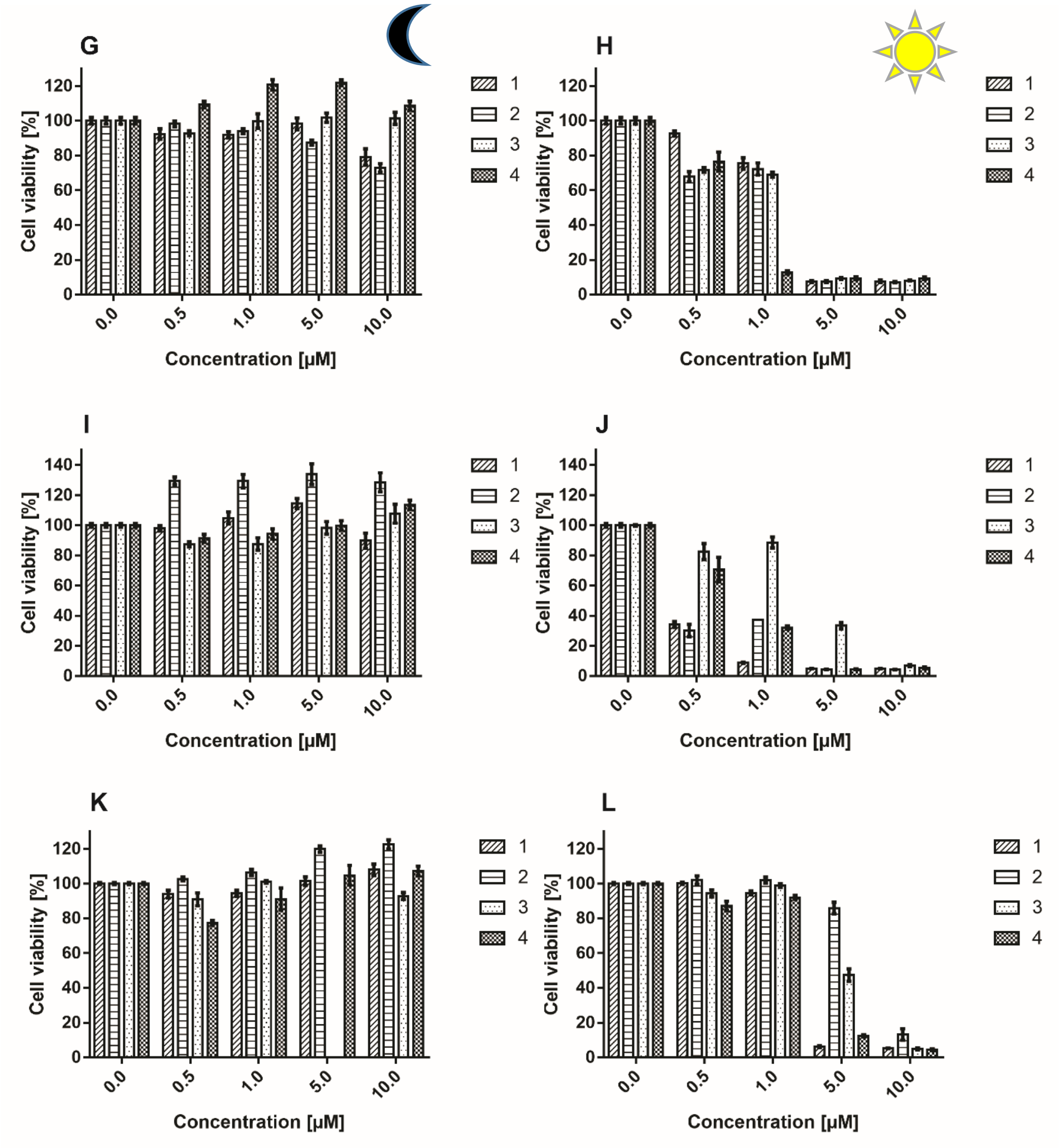


**Figure S18:** Photo- and dark toxicity of compounds **1–4** *in vitro*. Cell viability determined by WST-1 assay after 48 h of incubation with the tested compounds (24 h prior illumination + 24 h after illumination). Left panel dark toxicity (cells kept in the dark after the compound treatment), right panel phototoxicity (cell viability after compound photoactivation). A, B) MIA PaCa-2, C, D) PC-3, E, F) U-2 OS cells.

## Flow cytometry – raw data

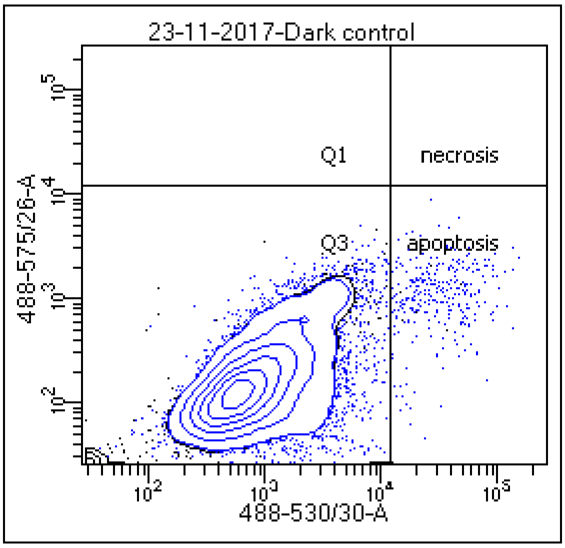


Tube: Dark control

| Population | #Events | %Parent | %Total |
|---|---|---|---|
| All Events | 10,000 | #### | 100.0 |
| P1 | 9,319 | 93.2 | 93.2 |
| P2 | 799 | 8.6 | 8.0 |
| P3 | 2,980 | 32.0 | 29.8 |
| Q1 | 0 | 0.0 | 0.0 |
| necrosis | 0 | 0.0 | 0.0 |
| Q3 | 8,932 | 95.8 | 89.3 |
| apoptosis | 387 | 4.2 | 3.9 |

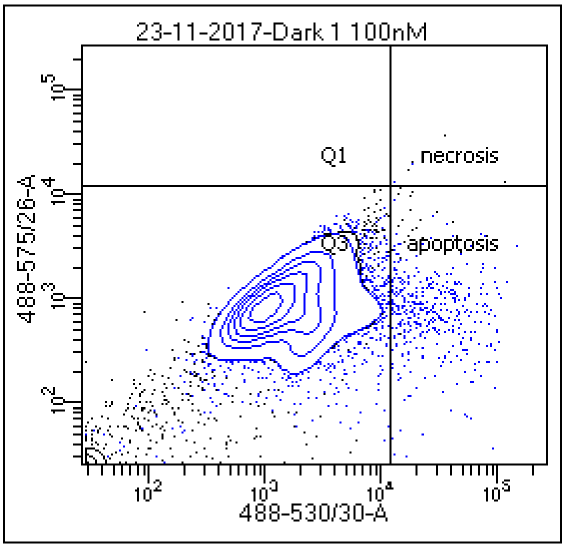


Tube: Dark 1 100nM

| Population | #Events | %Parent | %Total |
|---|---|---|---|
| All Events | 10,000 | #### | 100.0 |
| P1 | 9,192 | 91.9 | 91.9 |
| P2 | 1,179 | 12.8 | 11.8 |
| P3 | 8,920 | 97.0 | 89.2 |
| Q1 | 0 | 0.0 | 0.0 |
| necrosis | 2 | 0.0 | 0.0 |
| Q3 | 8,734 | 95.0 | 87.3 |
| apoptosis | 456 | 5.0 | 4.6 |

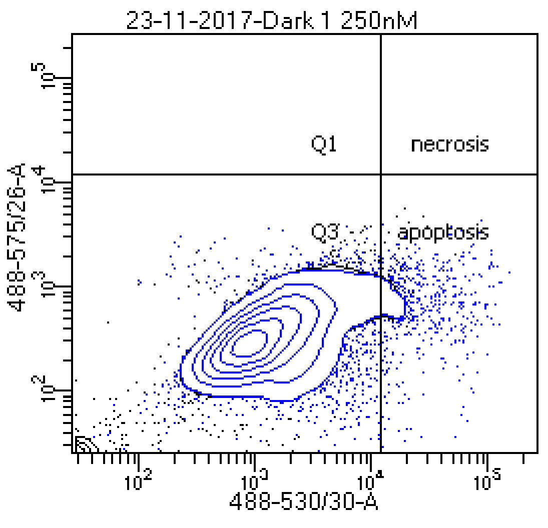


Tube: Dark 1 250nM

| Population | #Events | %Parent | %Total |
|---|---|---|---|
| All Events | 10,000 | #### | 100.0 |
| P1 | 9,209 | 92.1 | 92.1 |
| P2 | 1,413 | 15.3 | 14.1 |
| P3 | 6,839 | 74.3 | 68.4 |
| Q1 | 0 | 0.0 | 0.0 |
| necrosis | 0 | 0.0 | 0.0 |
| Q3 | 8,628 | 93.7 | 86.3 |
| apoptosis | 581 | 6.3 | 5.8 |

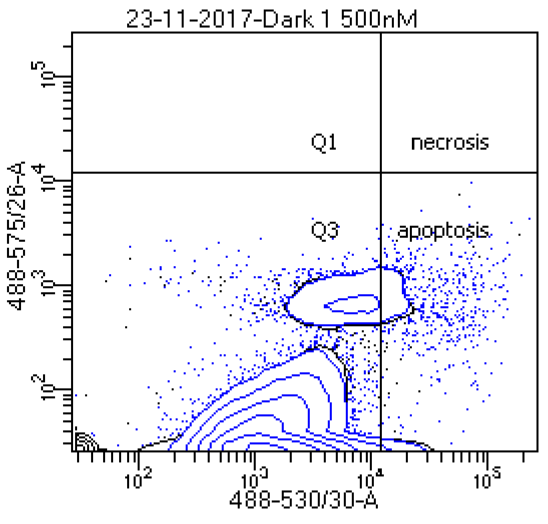


Tube: Dark 1 500nM

| Population | #Events | %Parent | %Total |
|---|---|---|---|
| All Events | 10,000 | #### | 100.0 |
| P1 | 9,120 | 91.2 | 91.2 |
| P2 | 2,090 | 22.9 | 20.9 |
| P3 | 1,547 | 17.0 | 15.5 |
| Q1 | 0 | 0.0 | 0.0 |
| necrosis | 1 | 0.0 | 0.0 |
| Q3 | 8,289 | 90.9 | 82.9 |
| apoptosis | 830 | 9.1 | 8.3 |

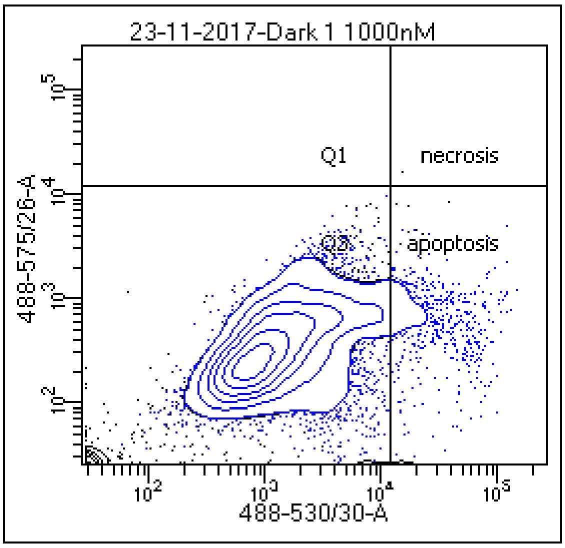


Tube: Dark 1 1000nM

| Population | #Events | %Parent | %Total |
|---|---|---|---|
| All Events | 10,000 | #### | 100.0 |
| P1 | 9,169 | 91.7 | 91.7 |
| P2 | 1,698 | 18.5 | 17.0 |
| P3 | 6,403 | 69.8 | 64.0 |
| Q1 | 0 | 0.0 | 0.0 |
| necrosis | 0 | 0.0 | 0.0 |
| Q3 | 8,466 | 92.3 | 84.7 |
| apoptosis | 703 | 7.7 | 7.0 |

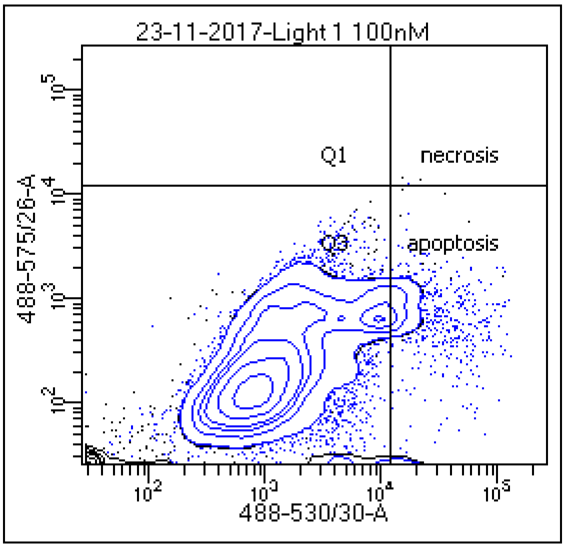


Tube: Light 1 100nM

| Population | #Events | %Parent | %Total |
|---|---|---|---|
| All Events | 10,000 | #### | 100.0 |
| P1 | 9,083 | 90.8 | 90.8 |
| P2 | 1,929 | 21.2 | 19.3 |
| P3 | 4,468 | 49.2 | 44.7 |
| Q1 | 0 | 0.0 | 0.0 |
| necrosis | 1 | 0.0 | 0.0 |
| Q3 | 8,262 | 91.0 | 82.6 |
| apoptosis | 820 | 9.0 | 8.2 |

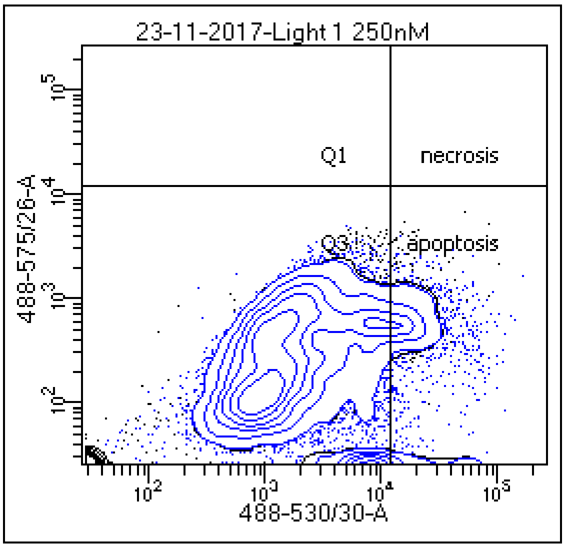


Tube: Light 1 250nM

| Population | #Events | %Parent | %Total |
|---|---|---|---|
| All Events | 10,000 | #### | 100.0 |
| P1 | 9,068 | 90.7 | 90.7 |
| P2 | 2,863 | 31.6 | 28.6 |
| P3 | 4,896 | 54.0 | 49.0 |
| Q1 | 0 | 0.0 | 0.0 |
| necrosis | 0 | 0.0 | 0.0 |
| Q3 | 7,925 | 87.4 | 79.2 |
| apoptosis | 1,143 | 12.6 | 11.4 |

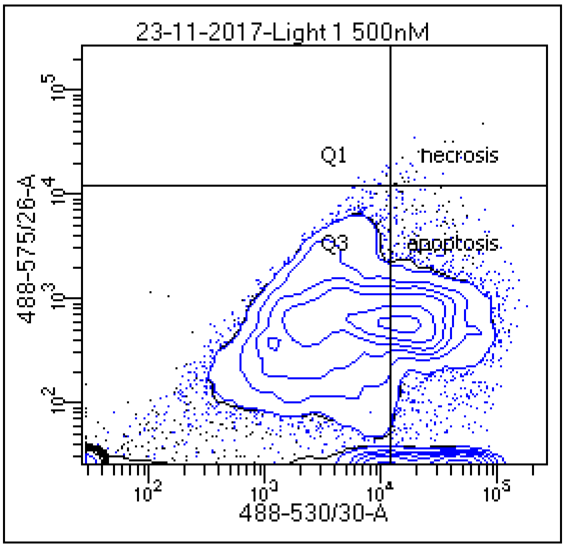


Tube: Light 1 500nM

| Population | #Events | %Parent | %Total |
|---|---|---|---|
| All Events | 10,000 | #### | 100.0 |
| P1 | 8,260 | 82.6 | 82.6 |
| P2 | 5,253 | 63.6 | 52.5 |
| P3 | 5,671 | 68.7 | 56.7 |
| Q1 | 15 | 0.2 | 0.2 |
| necrosis | 14 | 0.2 | 0.1 |
| Q3 | 5,111 | 61.9 | 51.1 |
| apoptosis | 3,120 | 37.8 | 31.2 |

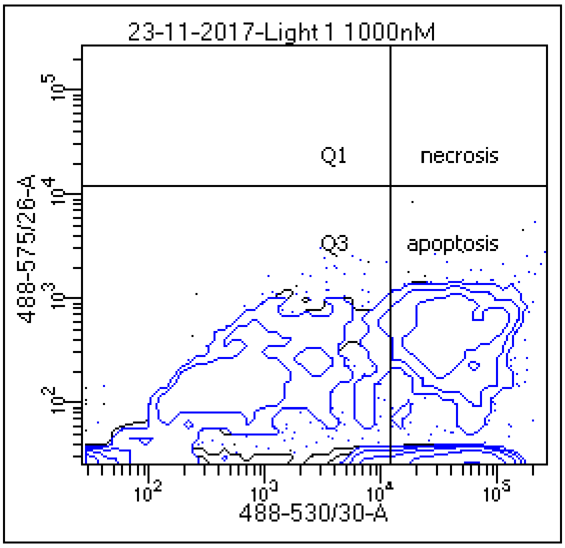


Tube: Light 1 1000nM

| Population | #Events | %Parent | %Total |
|---|---|---|---|
| All Events | 3,054 | #### | 100.0 |
| P1 | 1,766 | 57.8 | 57.8 |
| P2 | 1,175 | 66.5 | 38.5 |
| P3 | 660 | 37.4 | 21.6 |
| Q1 | 0 | 0.0 | 0.0 |
| necrosis | 1 | 0.1 | 0.0 |
| Q3 | 718 | 40.7 | 23.5 |
| apoptosis | 1,047 | 59.3 | 34.3 |

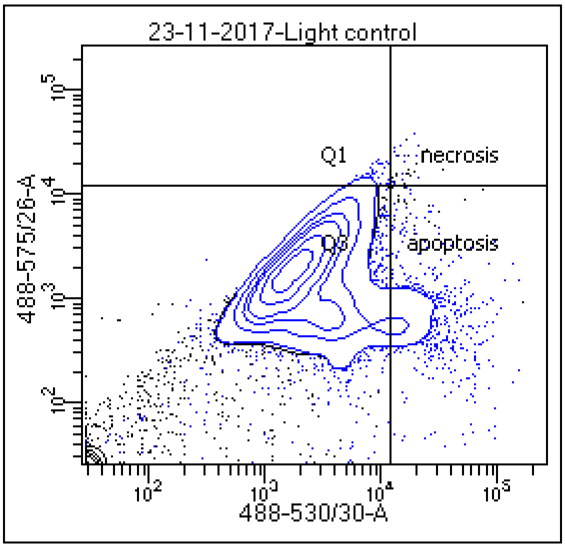


Tube: Light control

| Population | #Events | %Parent | %Total |
|---|---|---|---|
| All Events | 10,000 | #### | 100.0 |
| P1 | 8,908 | 89.1 | 89.1 |
| P2 | 2,563 | 28.8 | 25.6 |
| P3 | 8,607 | 96.6 | 86.1 |
| Q1 | 88 | 1.0 | 0.9 |
| necrosis | 14 | 0.2 | 0.1 |
| Q3 | 8,013 | 90.0 | 80.1 |
| apoptosis | 793 | 8.9 | 7.9 |

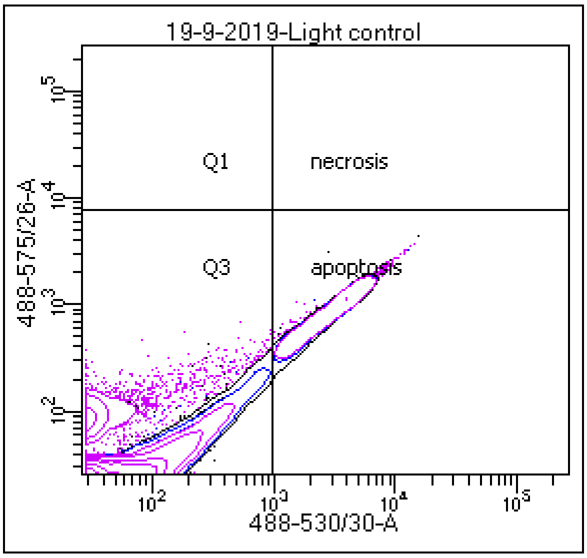


Tube: Light control

| Population | #Events | %Parent | %Total |
|---|---|---|---|
| All Events | 10,000 | #### | 100.0 |
| P1 | 8,858 | 88.6 | 88.6 |
| P2 | 7,383 | 83.3 | 73.8 |
| Q1 | 0 | 0.0 | 0.0 |
| necrosis | 0 | 0.0 | 0.0 |
| Q3 | 6,555 | 88.8 | 65.6 |
| apoptosis | 828 | 11.2 | 8.3 |

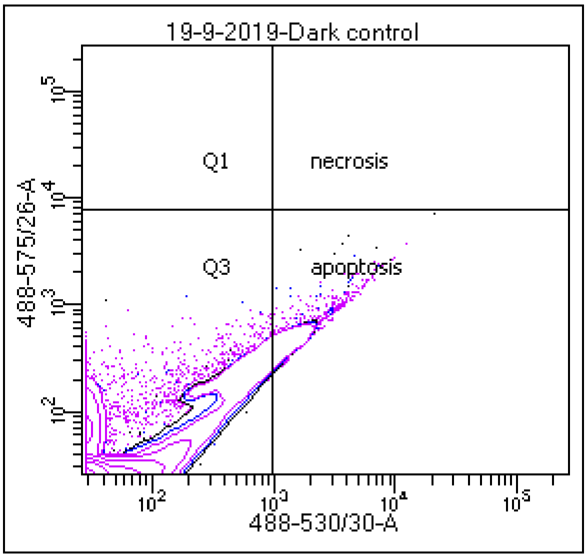


Tube: Dark control

| Population | #Events | %Parent | %Total |
|---|---|---|---|
| All Events | 10,000 | #### | 100.0 |
| P1 | 9,147 | 91.5 | 91.5 |
| P2 | 7,910 | 86.5 | 79.1 |
| Q1 | 0 | 0.0 | 0.0 |
| necrosis | 0 | 0.0 | 0.0 |
| Q3 | 7,439 | 94.0 | 74.4 |
| apoptosis | 471 | 6.0 | 4.7 |

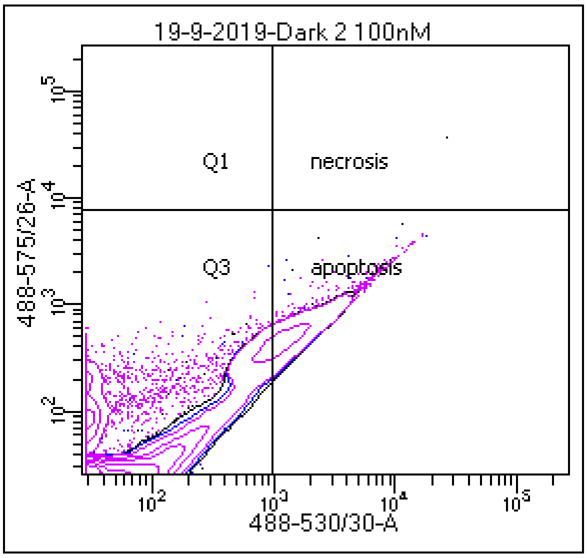


Tube: Dark 2 100nM

| Population | #Events | %Parent | %Total |
|---|---|---|---|
| All Events | 10,000 | #### | 100.0 |
| P1 | 8,877 | 88.8 | 88.8 |
| P2 | 7,541 | 84.9 | 75.4 |
| Q1 | 0 | 0.0 | 0.0 |
| necrosis | 0 | 0.0 | 0.0 |
| Q3 | 6,587 | 87.3 | 65.9 |
| apoptosis | 954 | 12.7 | 9.5 |

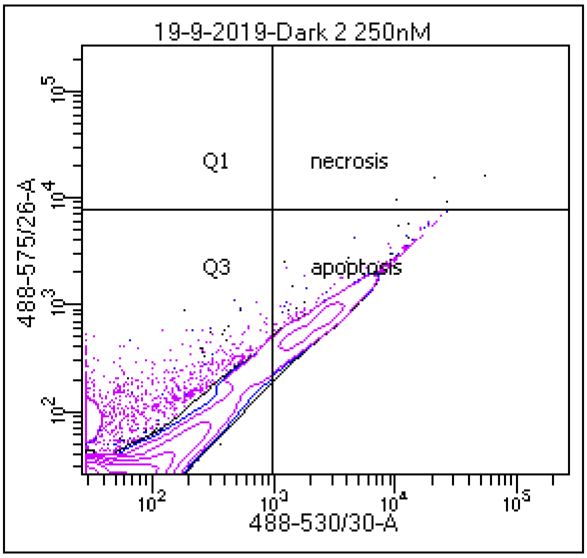


Tube: Dark 2 250nM

| Population | #Events | %Parent | %Total |
|---|---|---|---|
| All Events | 10,000 | #### | 100.0 |
| P1 | 8,817 | 88.2 | 88.2 |
| P2 | 7,419 | 84.1 | 74.2 |
| Q1 | 0 | 0.0 | 0.0 |
| necrosis | 1 | 0.0 | 0.0 |
| Q3 | 6,300 | 84.9 | 63.0 |
| apoptosis | 1,118 | 15.1 | 11.2 |

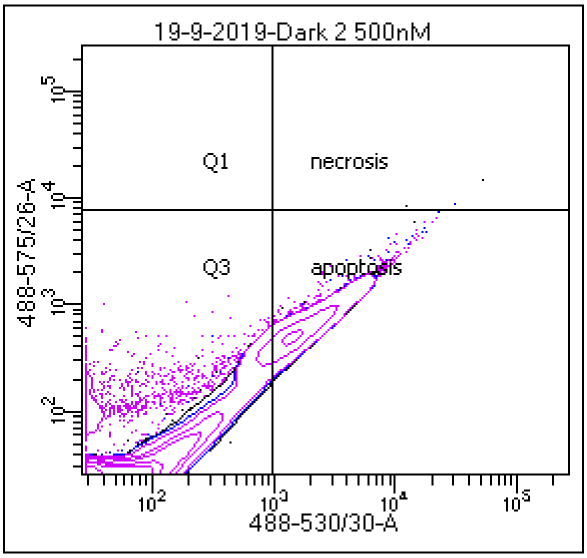


Tube: Dark 2 500nM

| Population | #Events | %Parent | %Total |
|---|---|---|---|
| All Events | 10,000 | #### | 100.0 |
| P1 | 9,006 | 90.1 | 90.1 |
| P2 | 7,750 | 86.1 | 77.5 |
| Q1 | 0 | 0.0 | 0.0 |
| necrosis | 0 | 0.0 | 0.0 |
| Q3 | 6,277 | 81.0 | 62.8 |
| apoptosis | 1,473 | 19.0 | 14.7 |

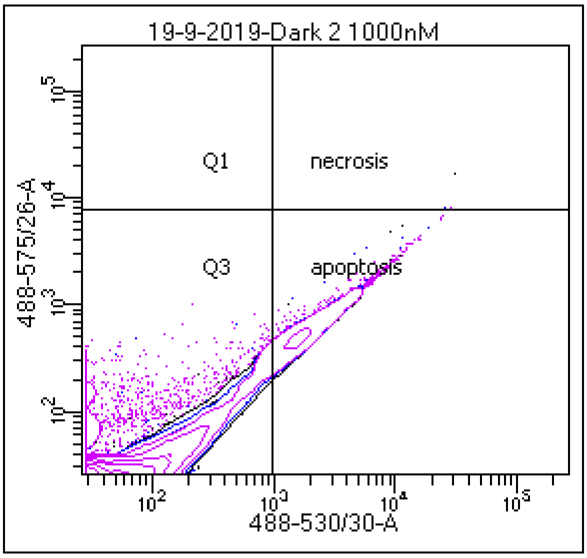


Tube: Dark 2 1000nM

| Population | #Events | %Parent | %Total |
|---|---|---|---|
| All Events | 10,000 | #### | 100.0 |
| P1 | 8,866 | 88.7 | 88.7 |
| P2 | 7,377 | 83.2 | 73.8 |
| Q1 | 0 | 0.0 | 0.0 |
| necrosis | 1 | 0.0 | 0.0 |
| Q3 | 6,421 | 87.0 | 64.2 |
| apoptosis | 955 | 12.9 | 9.6 |

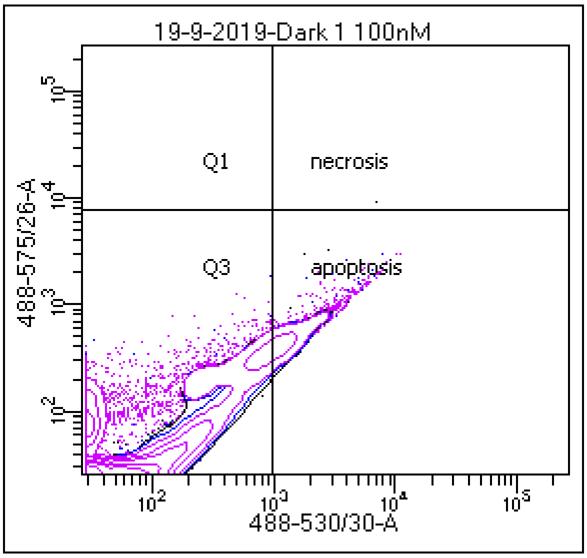


Tube: Dark 1 100nM

| Population | #Events | %Parent | %Total |
|---|---|---|---|
| All Events | 10,000 | #### | 100.0 |
| P1 | 8,945 | 89.4 | 89.4 |
| P2 | 7,589 | 84.8 | 75.9 |
| Q1 | 0 | 0.0 | 0.0 |
| necrosis | 0 | 0.0 | 0.0 |
| Q3 | 6,873 | 90.6 | 68.7 |
| apoptosis | 716 | 9.4 | 7.2 |

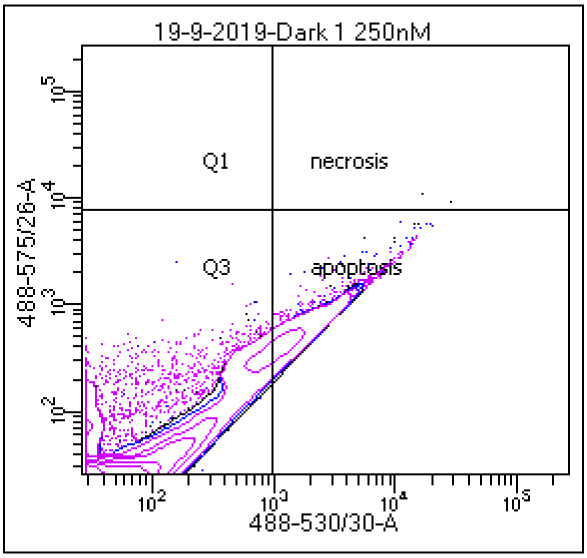


Tube: Dark 1 250nM

| Population | #Events | %Parent | %Total |
|---|---|---|---|
| All Events | 10,000 | #### | 100.0 |
| P1 | 9,084 | 90.8 | 90.8 |
| P2 | 7,803 | 85.9 | 78.0 |
| Q1 | 0 | 0.0 | 0.0 |
| necrosis | 0 | 0.0 | 0.0 |
| Q3 | 6,821 | 87.4 | 68.2 |
| apoptosis | 982 | 12.6 | 9.8 |

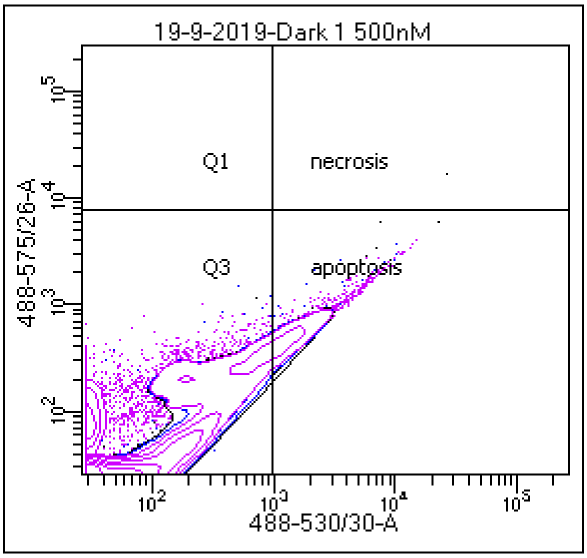


Tube: Dark 1 500nM

| Population | #Events | %Parent | %Total |
|---|---|---|---|
| All Events | 10,000 | #### | 100.0 |
| P1 | 8,889 | 88.9 | 88.9 |
| P2 | 7,524 | 84.6 | 75.2 |
| Q1 | 0 | 0.0 | 0.0 |
| necrosis | 0 | 0.0 | 0.0 |
| Q3 | 6,810 | 90.5 | 68.1 |
| apoptosis | 714 | 9.5 | 7.1 |

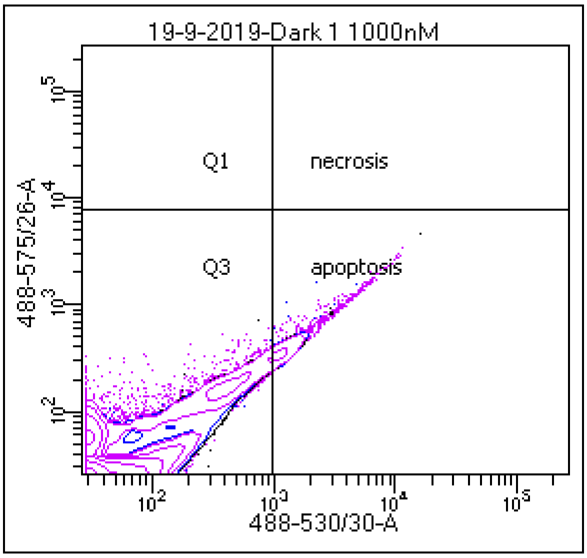


Tube: Dark 1 1000nM

| Population | #Events | %Parent | %Total |
|---|---|---|---|
| All Events | 10,000 | #### | 100.0 |
| P1 | 9,449 | 94.5 | 94.5 |
| P2 | 8,314 | 88.0 | 83.1 |
| Q1 | 0 | 0.0 | 0.0 |
| necrosis | 0 | 0.0 | 0.0 |
| Q3 | 7,700 | 92.6 | 77.0 |
| apoptosis | 614 | 7.4 | 6.1 |

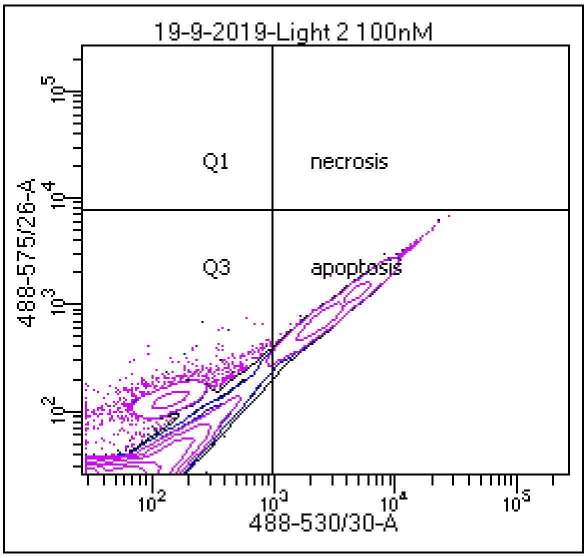


Tube: Light 2 100nM

| Population | #Events | %Parent | %Total |
|---|---|---|---|
| All Events | 10,000 | #### | 100.0 |
| P1 | 8,759 | 87.6 | 87.6 |
| P2 | 7,265 | 82.9 | 72.6 |
| Q1 | 0 | 0.0 | 0.0 |
| necrosis | 0 | 0.0 | 0.0 |
| Q3 | 5,983 | 82.4 | 59.8 |
| apoptosis | 1,282 | 17.6 | 12.8 |

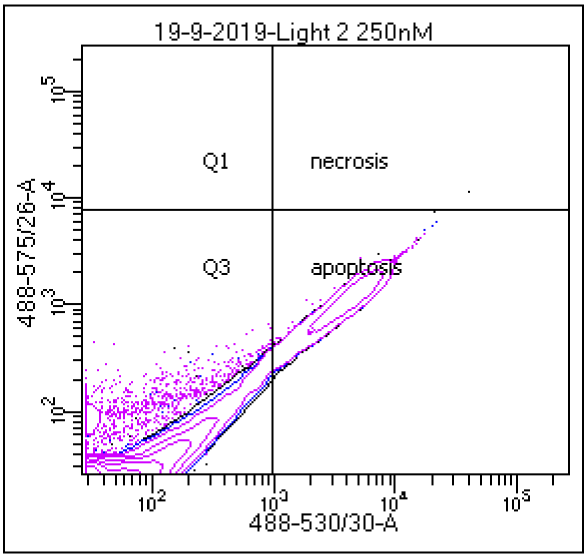


Tube: Light 2 250nM

| Population | #Events | %Parent | %Total |
|---|---|---|---|
| All Events | 10,000 | #### | 100.0 |
| P1 | 9,229 | 92.3 | 92.3 |
| P2 | 8,053 | 87.3 | 80.5 |
| Q1 | 0 | 0.0 | 0.0 |
| necrosis | 0 | 0.0 | 0.0 |
| Q3 | 6,685 | 83.0 | 66.8 |
| apoptosis | 1,368 | 17.0 | 13.7 |

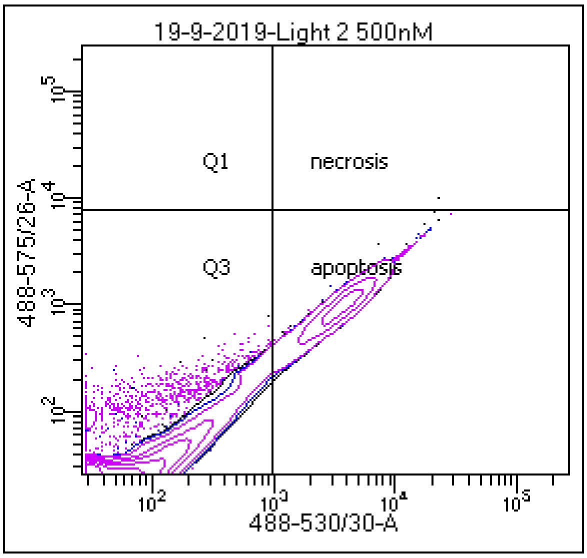


Tube: Light 2 500nM

| Population | #Events | %Parent | %Total |
|---|---|---|---|
| All Events | 10,000 | #### | 100.0 |
| P1 | 9,154 | 91.5 | 91.5 |
| P2 | 7,997 | 87.4 | 80.0 |
| Q1 | 0 | 0.0 | 0.0 |
| necrosis | 0 | 0.0 | 0.0 |
| Q3 | 6,281 | 78.5 | 62.8 |
| apoptosis | 1,716 | 21.5 | 17.2 |

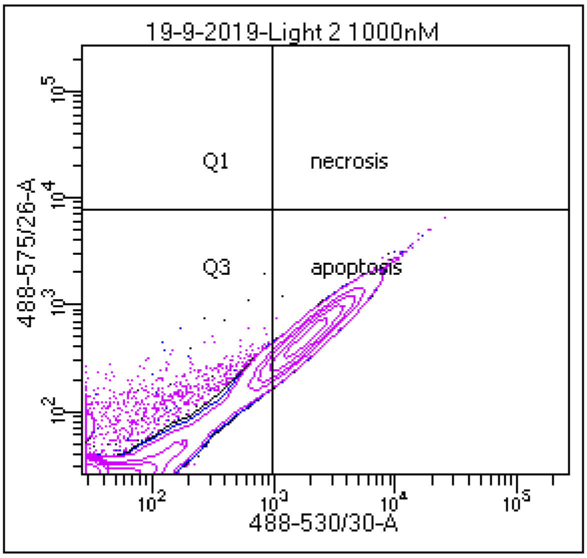


Tube: Light 2 1000nM

| Population | #Events | %Parent | %Total |
|---|---|---|---|
| All Events | 10,000 | #### | 100.0 |
| P1 | 9,476 | 94.8 | 94.8 |
| P2 | 8,560 | 90.3 | 85.6 |
| Q1 | 0 | 0.0 | 0.0 |
| necrosis | 0 | 0.0 | 0.0 |
| Q3 | 5,703 | 66.6 | 57.0 |
| apoptosis | 2,857 | 33.4 | 28.6 |

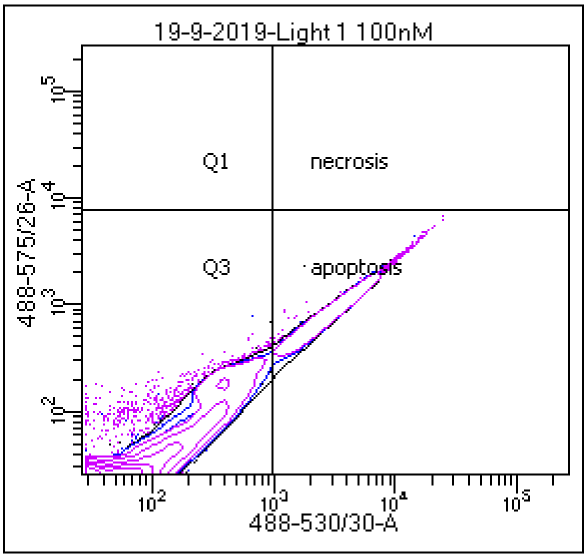


Tube: Light 1 100nM

| Population | #Events | %Parent | %Total |
|---|---|---|---|
| All Events | 10,000 | #### | 100.0 |
| P1 | 8,508 | 85.1 | 85.1 |
| P2 | 6,874 | 80.8 | 68.7 |
| Q1 | 0 | 0.0 | 0.0 |
| necrosis | 0 | 0.0 | 0.0 |
| Q3 | 5,781 | 84.1 | 57.8 |
| apoptosis | 1,093 | 15.9 | 10.9 |

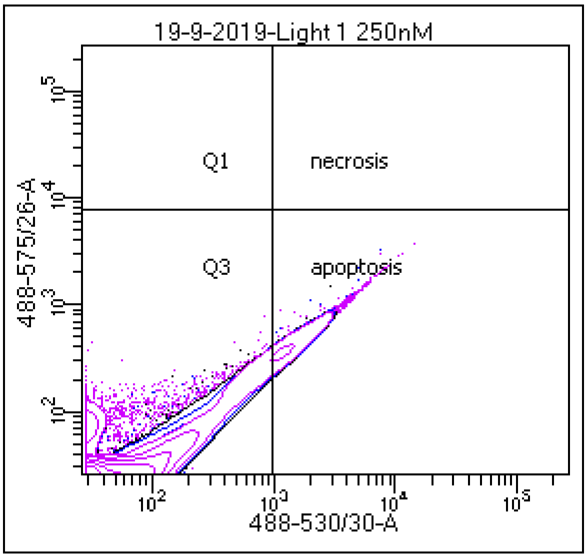


Tube: Light 1 250nM

| Population | #Events | %Parent | %Total |
|---|---|---|---|
| All Events | 10,000 | #### | 100.0 |
| P1 | 8,967 | 89.7 | 89.7 |
| P2 | 7,408 | 82.6 | 74.1 |
| Q1 | 0 | 0.0 | 0.0 |
| necrosis | 0 | 0.0 | 0.0 |
| Q3 | 6,625 | 89.4 | 66.2 |
| apoptosis | 783 | 10.6 | 7.8 |

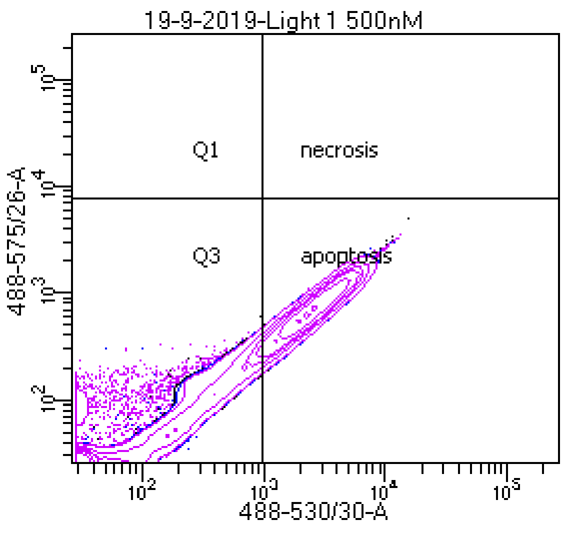


Tube: Light 1 500nM

| Population | #Events | %Parent | %Total |
|---|---|---|---|
| All Events | 10,000 | #### | 100.0 |
| P1 | 9,744 | 97.4 | 97.4 |
| P2 | 9,153 | 93.9 | 91.5 |
| Q1 | 0 | 0.0 | 0.0 |
| necrosis | 0 | 0.0 | 0.0 |
| Q3 | 5,542 | 60.5 | 55.4 |
| apoptosis | 3,611 | 39.5 | 36.1 |

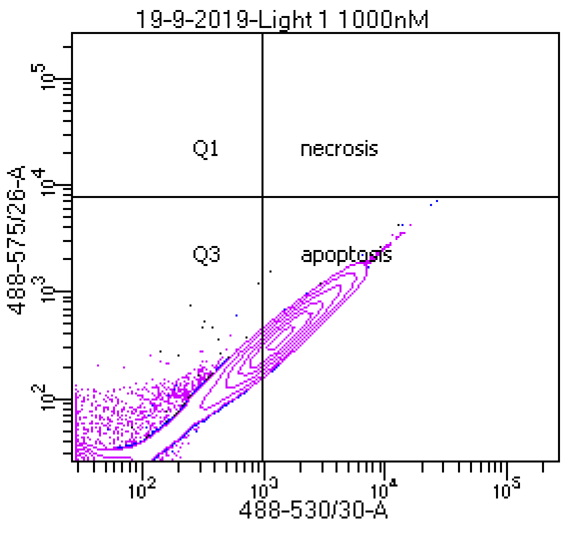


Tube: Light 1 1000nM

| Population | #Events | %Parent | %Total |
|---|---|---|---|
| All Events | 10,000 | #### | 100.0 |
| P1 | 9,923 | 99.2 | 99.2 |
| P2 | 9,493 | 95.7 | 94.9 |
| Q1 | 0 | 0.0 | 0.0 |
| necrosis | 0 | 0.0 | 0.0 |
| Q3 | 5,042 | 53.1 | 50.4 |
| apoptosis | 4,451 | 46.9 | 44.5 |

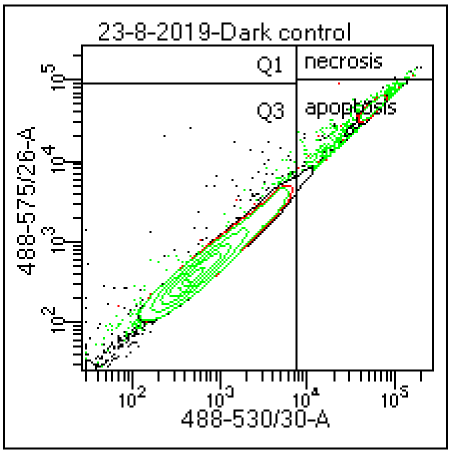


Tube: Dark control

| Population | #Events | %Parent | %Total |
|---|---|---|---|
| All Events | 10,000 | #### | 100.0 |
| P1 | 8,385 | 83.9 | 83.9 |
| P2 | 6,835 | 81.5 | 68.4 |
| Q1 | 0 | 0.0 | 0.0 |
| necrosis | 15 | 0.2 | 0.2 |
| Q3 | 6,081 | 89.0 | 60.8 |
| apoptosis | 739 | 10.8 | 7.4 |

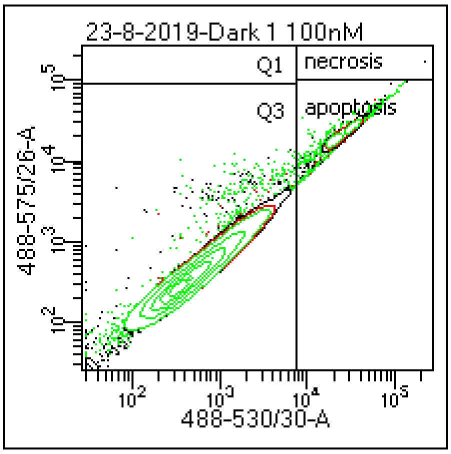


Tube: Dark 1 100nM

| Population | #Events | %Parent | %Total |
|---|---|---|---|
| All Events | 10,000 | #### | 100.0 |
| P1 | 8,582 | 85.8 | 85.8 |
| P2 | 7,147 | 83.3 | 71.5 |
| Q1 | 0 | 0.0 | 0.0 |
| necrosis | 1 | 0.0 | 0.0 |
| Q3 | 6,557 | 91.7 | 65.6 |
| apoptosis | 589 | 8.2 | 5.9 |

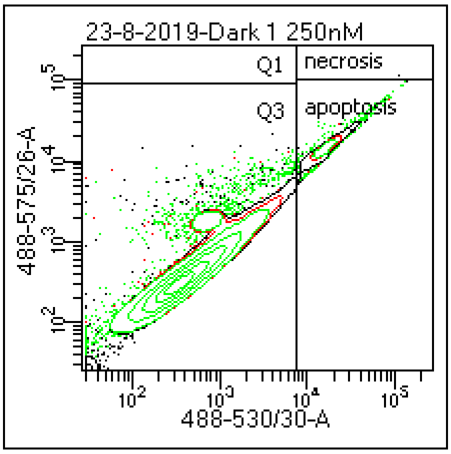


Tube: Dark 1 250nM

| Population | #Events | %Parent | %Total |
|---|---|---|---|
| All Events | 10,000 | #### | 100.0 |
| P1 | 8,515 | 85.2 | 85.2 |
| P2 | 7,004 | 82.3 | 70.0 |
| Q1 | 0 | 0.0 | 0.0 |
| necrosis | 1 | 0.0 | 0.0 |
| Q3 | 6,576 | 93.9 | 65.8 |
| apoptosis | 427 | 6.1 | 4.3 |

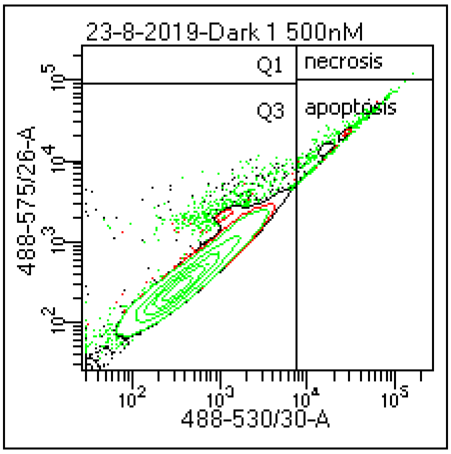


Tube: Dark 1 500nM

| Population | #Events | %Parent | %Total |
|---|---|---|---|
| All Events | 10,000 | #### | 100.0 |
| P1 | 8,585 | 85.9 | 85.9 |
| P2 | 7,063 | 82.3 | 70.6 |
| Q1 | 0 | 0.0 | 0.0 |
| necrosis | 2 | 0.0 | 0.0 |
| Q3 | 6,654 | 94.2 | 66.5 |
| apoptosis | 407 | 5.8 | 4.1 |

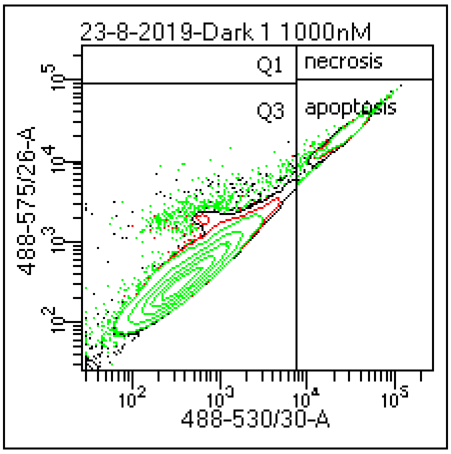


Tube: Dark 1 1000nM

| Population | #Events | %Parent | %Total |
|---|---|---|---|
| All Events | 10,000 | #### | 100.0 |
| P1 | 8,840 | 88.4 | 88.4 |
| P2 | 7,505 | 84.9 | 75.0 |
| Q1 | 0 | 0.0 | 0.0 |
| necrosis | 0 | 0.0 | 0.0 |
| Q3 | 7,006 | 93.4 | 70.1 |
| apoptosis | 499 | 6.6 | 5.0 |

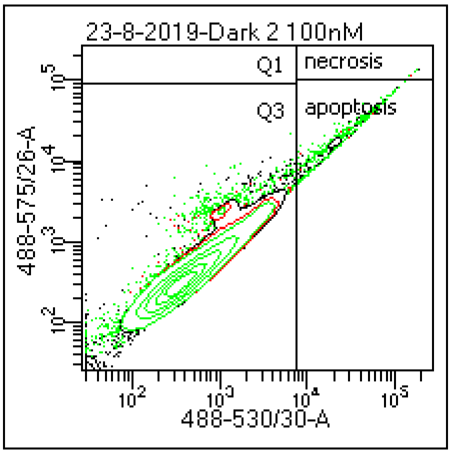


Tube: Dark 2 100nM

| Population | #Events | %Parent | %Total |
|---|---|---|---|
| All Events | 10,000 | #### | 100.0 |
| P1 | 8,412 | 84.1 | 84.1 |
| P2 | 6,881 | 81.8 | 68.8 |
| Q1 | 0 | 0.0 | 0.0 |
| necrosis | 5 | 0.1 | 0.0 |
| Q3 | 6,491 | 94.3 | 64.9 |
| apoptosis | 385 | 5.6 | 3.8 |

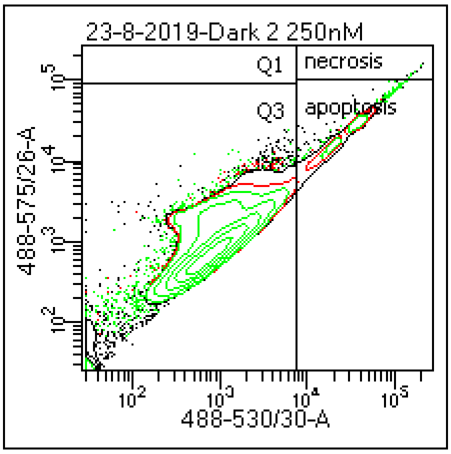


Tube: Dark 2 250nM

| Population | #Events | %Parent | %Total |
|---|---|---|---|
| All Events | 10,000 | #### | 100.0 |
| P1 | 8,117 | 81.2 | 81.2 |
| P2 | 6,661 | 82.1 | 66.6 |
| Q1 | 0 | 0.0 | 0.0 |
| necrosis | 18 | 0.3 | 0.2 |
| Q3 | 6,109 | 91.7 | 61.1 |
| apoptosis | 534 | 8.0 | 5.3 |

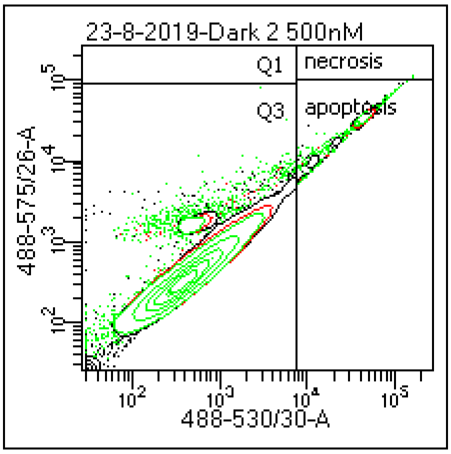


Tube: Dark 2 500nM

| Population | #Events | %Parent | %Total |
|---|---|---|---|
| All Events | 10,000 | #### | 100.0 |
| P1 | 8,206 | 82.1 | 82.1 |
| P2 | 6,687 | 81.5 | 66.9 |
| Q1 | 0 | 0.0 | 0.0 |
| necrosis | 3 | 0.0 | 0.0 |
| Q3 | 6,325 | 94.6 | 63.2 |
| apoptosis | 359 | 5.4 | 3.6 |

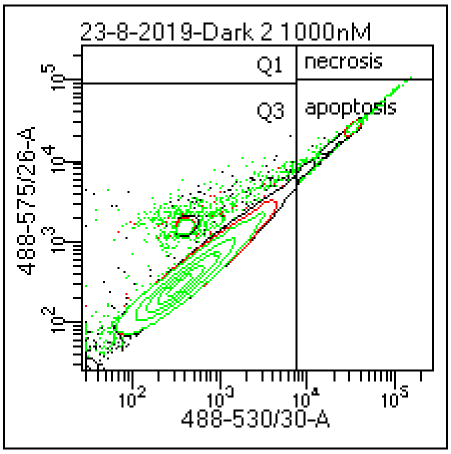


Tube: Dark 2 1000nM

| Population | #Events | %Parent | %Total |
|---|---|---|---|
| All Events | 10,000 | #### | 100.0 |
| P1 | 8,371 | 83.7 | 83.7 |
| P2 | 6,699 | 80.0 | 67.0 |
| Q1 | 0 | 0.0 | 0.0 |
| necrosis | 3 | 0.0 | 0.0 |
| Q3 | 6,308 | 94.2 | 63.1 |
| apoptosis | 388 | 5.8 | 3.9 |

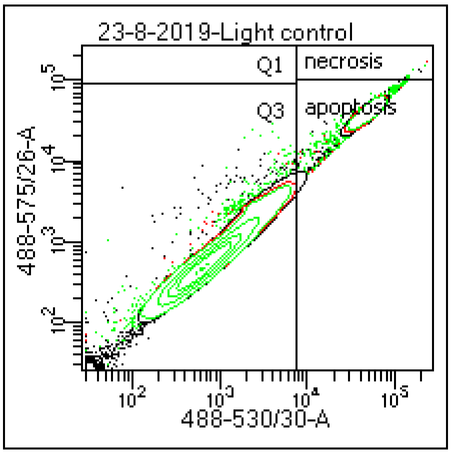


Tube: Light control

| Population | #Events | %Parent | %Total |
|---|---|---|---|
| All Events | 10,000 | #### | 100.0 |
| P1 | 6,626 | 66.3 | 66.3 |
| P2 | 5,485 | 82.8 | 54.8 |
| Q1 | 0 | 0.0 | 0.0 |
| necrosis | 18 | 0.3 | 0.2 |
| Q3 | 4,874 | 88.9 | 48.7 |
| apoptosis | 593 | 10.8 | 5.9 |

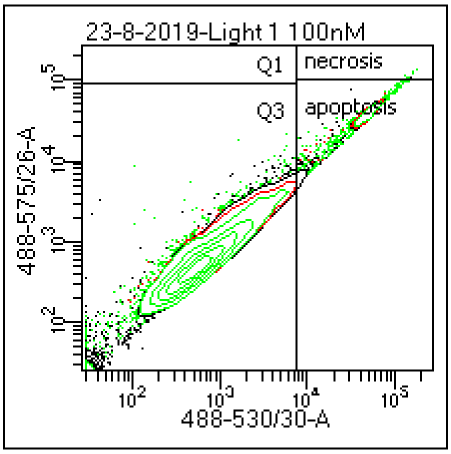


Tube: Light 1 100nM

| Population | #Events | %Parent | %Total |
|---|---|---|---|
| All Events | 10,000 | #### | 100.0 |
| P1 | 7,702 | 77.0 | 77.0 |
| P2 | 6,400 | 83.1 | 64.0 |
| Q1 | 0 | 0.0 | 0.0 |
| necrosis | 16 | 0.2 | 0.2 |
| Q3 | 5,893 | 92.1 | 58.9 |
| apoptosis | 491 | 7.7 | 4.9 |

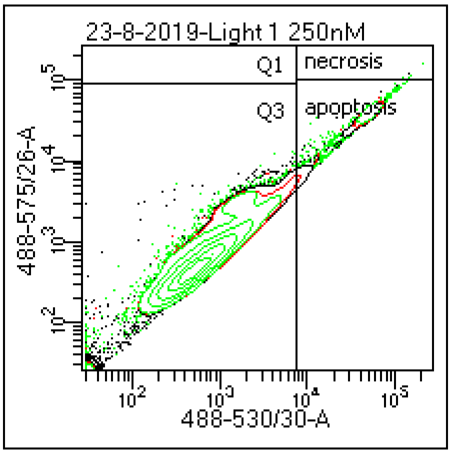


Tube: Light 1 250nM

| Population | #Events | %Parent | %Total |
|---|---|---|---|
| All Events | 10,000 | #### | 100.0 |
| P1 | 7,595 | 75.9 | 75.9 |
| P2 | 6,345 | 83.5 | 63.4 |
| Q1 | 0 | 0.0 | 0.0 |
| necrosis | 9 | 0.1 | 0.1 |
| Q3 | 5,789 | 91.2 | 57.9 |
| apoptosis | 547 | 8.6 | 5.5 |

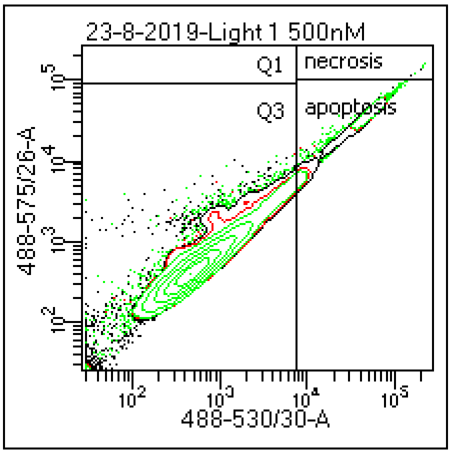


Tube: Light 1 500nM

| Population | #Events | %Parent | %Total |
|---|---|---|---|
| All Events | 10,000 | #### | 100.0 |
| P1 | 7,633 | 76.3 | 76.3 |
| P2 | 6,313 | 82.7 | 63.1 |
| Q1 | 0 | 0.0 | 0.0 |
| necrosis | 9 | 0.1 | 0.1 |
| Q3 | 5,697 | 90.2 | 57.0 |
| apoptosis | 607 | 9.6 | 6.1 |

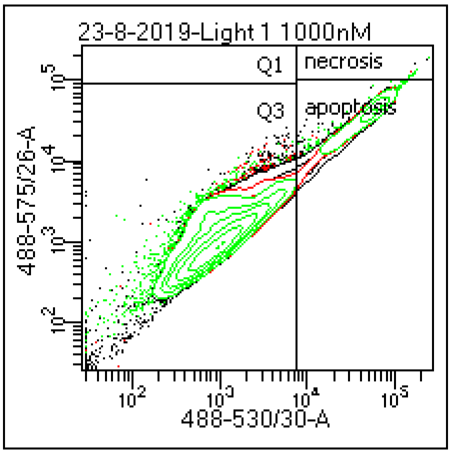


Tube: Light 1 1000nM

| Population | #Events | %Parent | %Total |
|---|---|---|---|
| All Events | 10,000 | #### | 100.0 |
| P1 | 8,207 | 82.1 | 82.1 |
| P2 | 6,950 | 84.7 | 69.5 |
| Q1 | 0 | 0.0 | 0.0 |
| necrosis | 31 | 0.4 | 0.3 |
| Q3 | 5,804 | 83.5 | 58.0 |
| apoptosis | 1,115 | 16.0 | 11.2 |

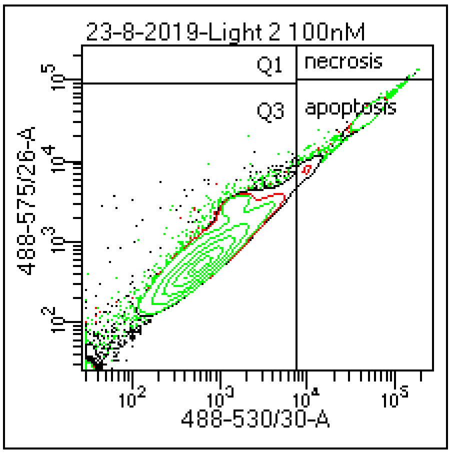


Tube: Light 2 100nM

| Population | #Events | %Parent | %Total |
|---|---|---|---|
| All Events | 10,000 | #### | 100.0 |
| P1 | 7,130 | 71.3 | 71.3 |
| P2 | 5,833 | 81.8 | 58.3 |
| Q1 | 0 | 0.0 | 0.0 |
| necrosis | 11 | 0.2 | 0.1 |
| Q3 | 5,241 | 89.9 | 52.4 |
| apoptosis | 581 | 10.0 | 5.8 |

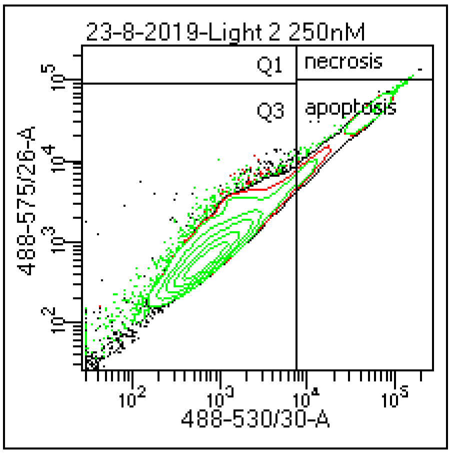


Tube: Light 2 250nM

| Population | #Events | %Parent | %Total |
|---|---|---|---|
| All Events | 10,000 | #### | 100.0 |
| P1 | 8,129 | 81.3 | 81.3 |
| P2 | 6,733 | 82.8 | 67.3 |
| Q1 | 0 | 0.0 | 0.0 |
| necrosis | 17 | 0.3 | 0.2 |
| Q3 | 5,800 | 86.1 | 58.0 |
| apoptosis | 916 | 13.6 | 9.2 |

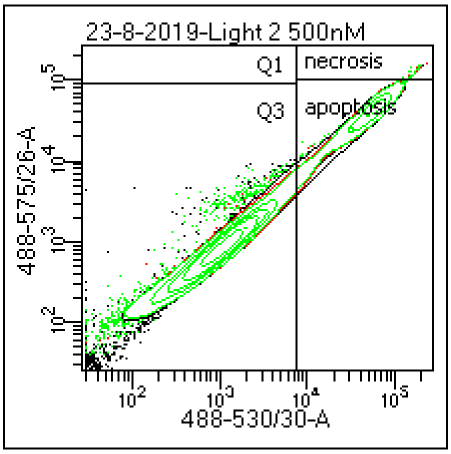


Tube: Light 2 500nM

| Population | #Events | %Parent | %Total |
|---|---|---|---|
| All Events | 10,000 | #### | 100.0 |
| P1 | 7,719 | 77.2 | 77.2 |
| P2 | 6,793 | 88.0 | 67.9 |
| Q1 | 0 | 0.0 | 0.0 |
| necrosis | 60 | 0.9 | 0.6 |
| Q3 | 5,324 | 78.4 | 53.2 |
| apoptosis | 1,409 | 20.7 | 14.1 |

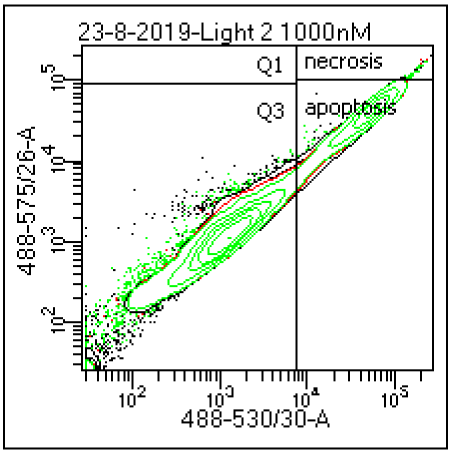


Tube: Light 2 1000nM

| Population | #Events | %Parent | %Total |
|---|---|---|---|
| All Events | 10,000 | #### | 100.0 |
| P1 | 7,764 | 77.6 | 77.6 |
| P2 | 6,751 | 87.0 | 67.5 |
| Q1 | 0 | 0.0 | 0.0 |
| necrosis | 70 | 1.0 | 0.7 |
| Q3 | 5,143 | 76.2 | 51.4 |
| apoptosis | 1,538 | 22.8 | 15.4 |

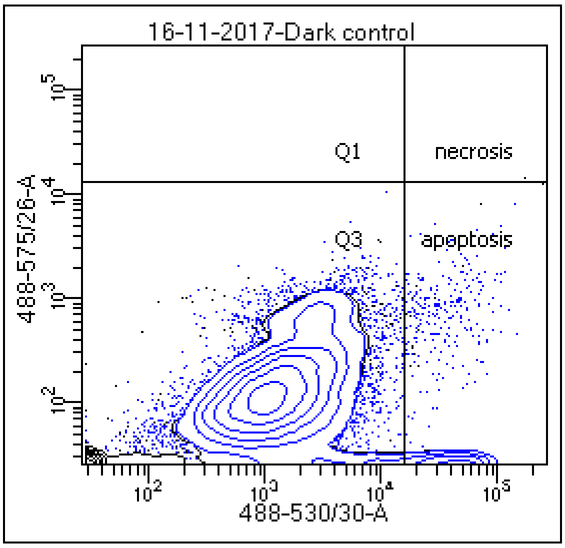


Tube: Dark control

| Population | #Events | %Parent | %Total |
|---|---|---|---|
| All Events | 10,000 | #### | 100.0 |
| P1 | 8,936 | 89.4 | 89.4 |
| P2 | 1,971 | 22.1 | 19.7 |
| P3 | 2,339 | 26.2 | 23.4 |
| Q1 | 0 | 0.0 | 0.0 |
| necrosis | 0 | 0.0 | 0.0 |
| Q3 | 7,997 | 89.5 | 80.0 |
| apoptosis | 939 | 10.5 | 9.4 |

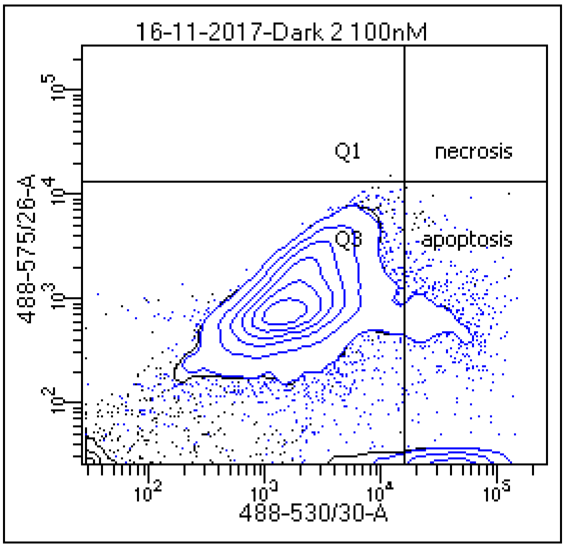


Tube: Dark 2 100nM

| Population | #Events | %Parent | %Total |
|---|---|---|---|
| All Events | 10,000 | #### | 100.0 |
| P1 | 9,081 | 90.8 | 90.8 |
| P2 | 2,426 | 26.7 | 24.3 |
| P3 | 7,852 | 86.5 | 78.5 |
| Q1 | 1 | 0.0 | 0.0 |
| necrosis | 0 | 0.0 | 0.0 |
| Q3 | 7,921 | 87.2 | 79.2 |
| apoptosis | 1,159 | 12.8 | 11.6 |

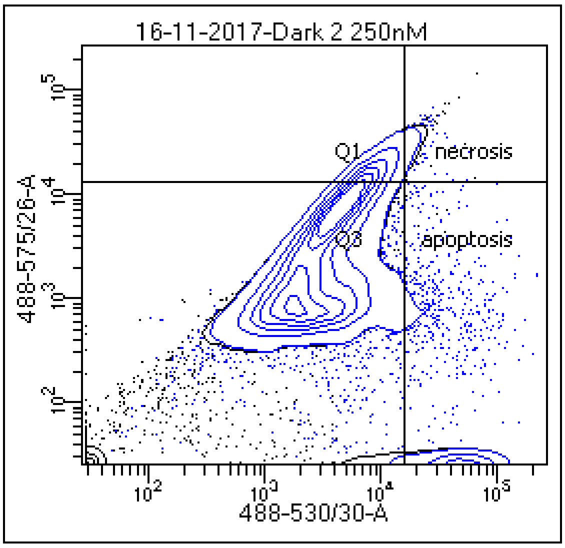


Tube: Dark 2 250nM

| Population | #Events | %Parent | %Total |
|---|---|---|---|
| All Events | 10,000 | #### | 100.0 |
| P1 | 9,045 | 90.4 | 90.4 |
| P2 | 4,138 | 45.7 | 41.4 |
| P3 | 8,196 | 90.6 | 82.0 |
| Q1 | 762 | 8.4 | 7.6 |
| necrosis | 117 | 1.3 | 1.2 |
| Q3 | 7,162 | 79.2 | 71.6 |
| apoptosis | 1,004 | 11.1 | 10.0 |

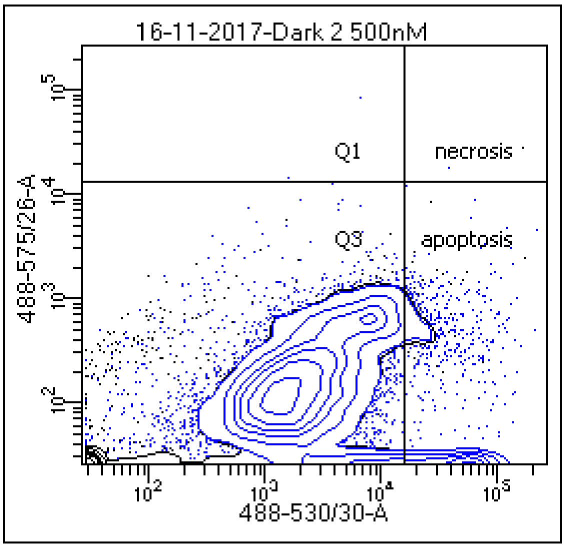


Tube: Dark 2 500nM

| Population | #Events | %Parent | %Total |
|---|---|---|---|
| All Events | 10,000 | #### | 100.0 |
| P1 | 8,900 | 89.0 | 89.0 |
| P2 | 3,169 | 35.6 | 31.7 |
| P3 | 2,972 | 33.4 | 29.7 |
| Q1 | 3 | 0.0 | 0.0 |
| necrosis | 1 | 0.0 | 0.0 |
| Q3 | 7,708 | 86.6 | 77.1 |
| apoptosis | 1,188 | 13.3 | 11.9 |

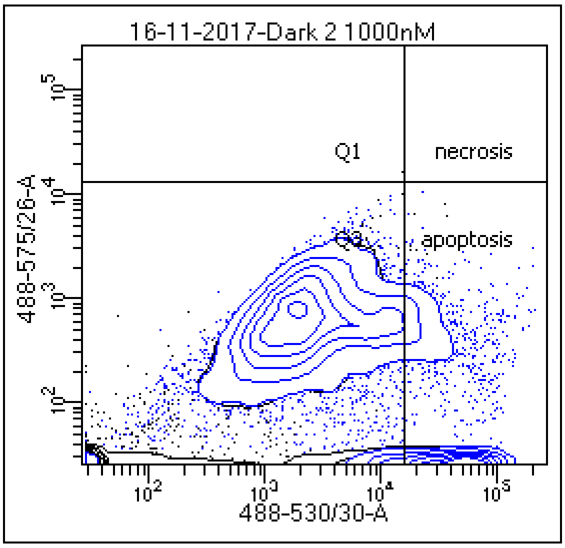


Tube: Dark 2 1000nM

| Population | #Events | %Parent | %Total |
|---|---|---|---|
| All Events | 10,000 | #### | 100.0 |
| P1 | 8,434 | 84.3 | 84.3 |
| P2 | 3,766 | 44.7 | 37.7 |
| P3 | 5,828 | 69.1 | 58.3 |
| Q1 | 0 | 0.0 | 0.0 |
| necrosis | 0 | 0.0 | 0.0 |
| Q3 | 6,459 | 76.6 | 64.6 |
| apoptosis | 1,975 | 23.4 | 19.8 |

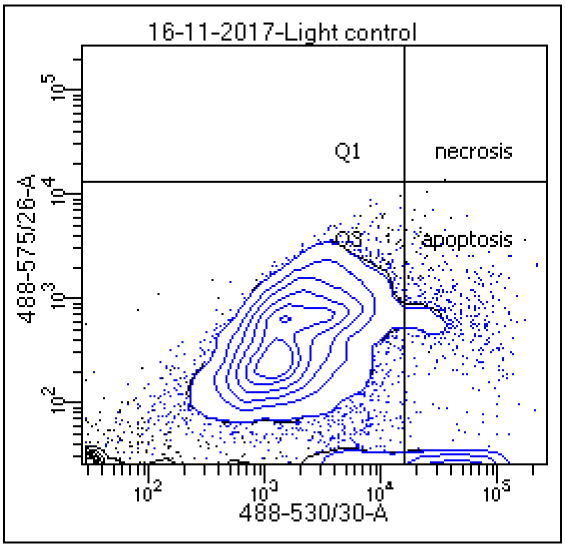


Tube: Light control

| Population | #Events | %Parent | %Total |
|---|---|---|---|
| All Events | 10,000 | #### | 100.0 |
| P1 | 8,988 | 89.9 | 89.9 |
| P2 | 2,496 | 27.8 | 25.0 |
| P3 | 5,982 | 66.6 | 59.8 |
| Q1 | 0 | 0.0 | 0.0 |
| necrosis | 0 | 0.0 | 0.0 |
| Q3 | 7,993 | 88.9 | 79.9 |
| apoptosis | 995 | 11.1 | 10.0 |

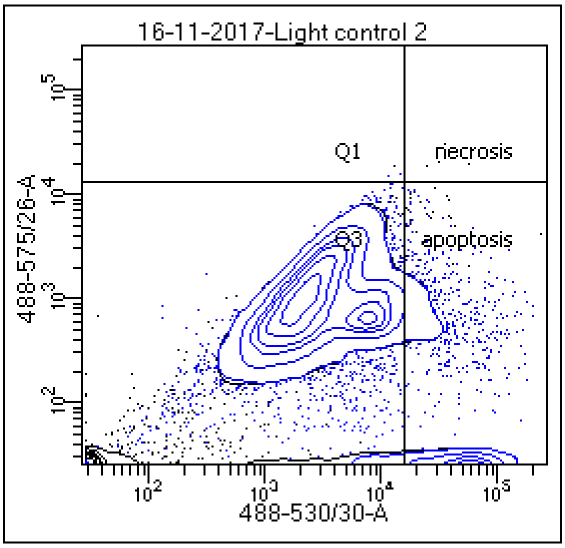


Tube: Light control 2

| Population | #Events | %Parent | %Total |
|---|---|---|---|
| All Events | 10,000 | #### | 100.0 |
| P1 | 8,823 | 88.2 | 88.2 |
| P2 | 3,935 | 44.6 | 39.4 |
| P3 | 7,387 | 83.7 | 73.9 |
| Q1 | 7 | 0.1 | 0.1 |
| necrosis | 0 | 0.0 | 0.0 |
| Q3 | 7,501 | 85.0 | 75.0 |
| apoptosis | 1,315 | 14.9 | 13.2 |

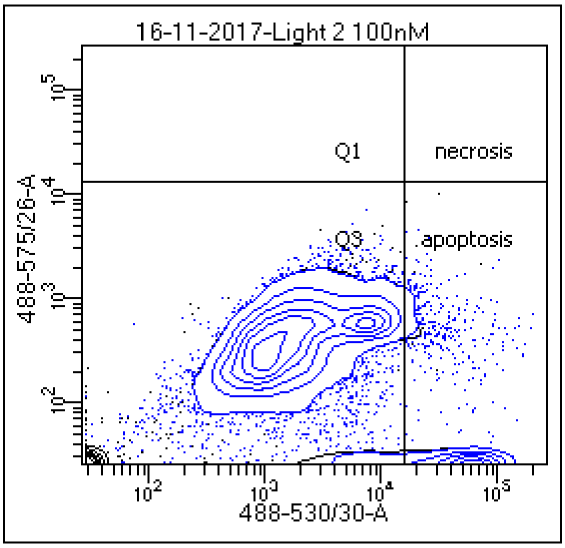


Tube: Light 2 100nM

| Population | #Events | %Parent | %Total |
|---|---|---|---|
| All Events | 10,000 | #### | 100.0 |
| P1 | 8,981 | 89.8 | 89.8 |
| P2 | 3,043 | 33.9 | 30.4 |
| P3 | 5,979 | 66.6 | 59.8 |
| Q1 | 0 | 0.0 | 0.0 |
| necrosis | 0 | 0.0 | 0.0 |
| Q3 | 7,710 | 85.8 | 77.1 |
| apoptosis | 1,271 | 14.2 | 12.7 |

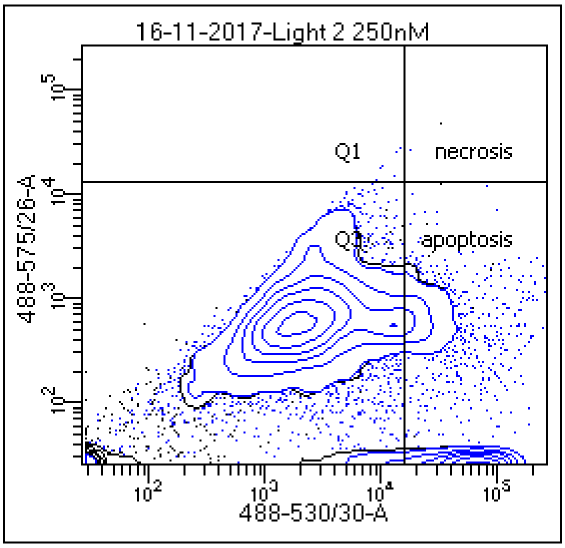


Tube: Light 2 250nM

| Population | #Events | %Parent | %Total |
|---|---|---|---|
| All Events | 10,000 | #### | 100.0 |
| P1 | 8,743 | 87.4 | 87.4 |
| P2 | 3,793 | 43.4 | 37.9 |
| P3 | 6,522 | 74.6 | 65.2 |
| Q1 | 7 | 0.1 | 0.1 |
| necrosis | 1 | 0.0 | 0.0 |
| Q3 | 6,738 | 77.1 | 67.4 |
| apoptosis | 1,997 | 22.8 | 20.0 |

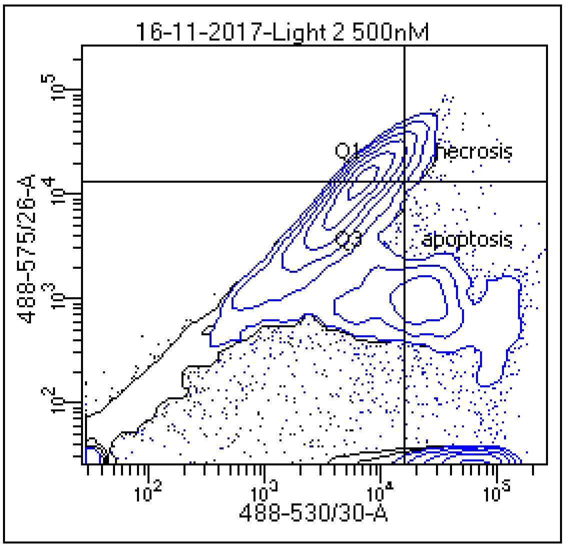


Tube: Light 2 500nM

| Population | #Events | %Parent | %Total |
|---|---|---|---|
| All Events | 8,051 | #### | 100.0 |
| P1 | 6,413 | 79.7 | 79.7 |
| P2 | 4,839 | 75.5 | 60.1 |
| P3 | 5,286 | 82.4 | 65.7 |
| Q1 | 1,203 | 18.8 | 14.9 |
| necrosis | 228 | 3.6 | 2.8 |
| Q3 | 3,049 | 47.5 | 37.9 |
| apoptosis | 1,933 | 30.1 | 24.0 |

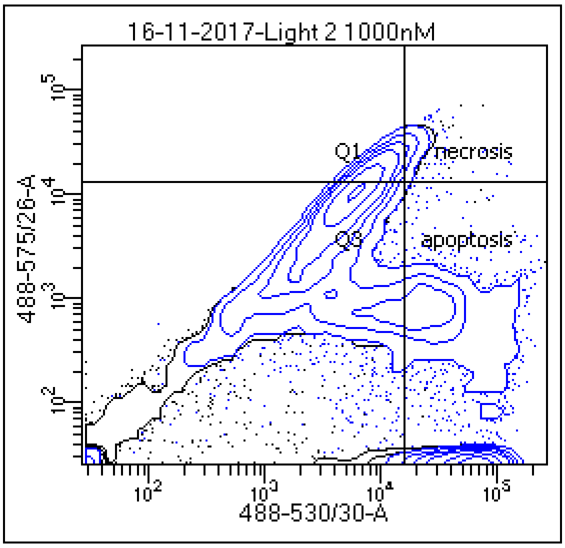


Tube: Light 2 1000nM

| Population | #Events | %Parent | %Total |
|---|---|---|---|
| All Events | 8,150 | #### | 100.0 |
| P1 | 6,653 | 81.6 | 81.6 |
| P2 | 4,783 | 71.9 | 58.7 |
| P3 | 5,028 | 75.6 | 61.7 |
| Q1 | 703 | 10.6 | 8.6 |
| necrosis | 125 | 1.9 | 1.5 |
| Q3 | 3,432 | 51.6 | 42.1 |
| apoptosis | 2,393 | 36.0 | 29.4 |

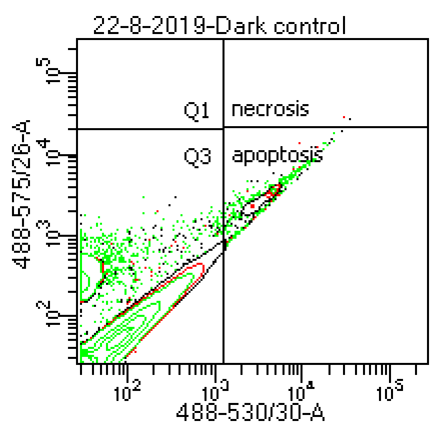


Tube: Dark control

| Population | #Events | %Parent | %Total |
|---|---|---|---|
| All Events | 10,000 | #### | 100.0 |
| P1 | 7,424 | 74.2 | 74.2 |
| P2 | 6,228 | 83.9 | 62.3 |
| Q1 | 0 | 0.0 | 0.0 |
| necrosis | 0 | 0.0 | 0.0 |
| Q3 | 5,834 | 93.7 | 58.3 |
| apoptosis | 394 | 6.3 | 3.9 |

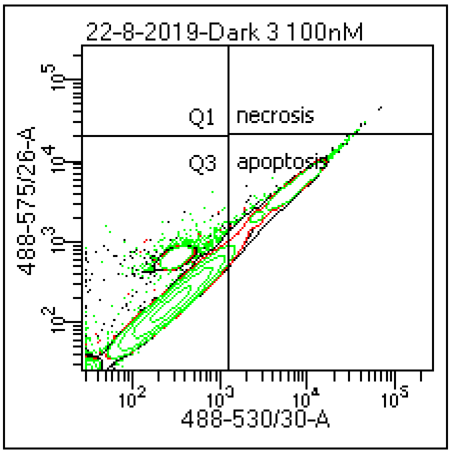


Tube: Dark 3 100nM

| Population | #Events | %Parent | %Total |
|---|---|---|---|
| All Events | 10,000 | #### | 100.0 |
| P1 | 6,694 | 66.9 | 66.9 |
| P2 | 5,704 | 85.2 | 57.0 |
| Q1 | 0 | 0.0 | 0.0 |
| necrosis | 7 | 0.1 | 0.1 |
| Q3 | 4,727 | 82.9 | 47.3 |
| apoptosis | 970 | 17.0 | 9.7 |

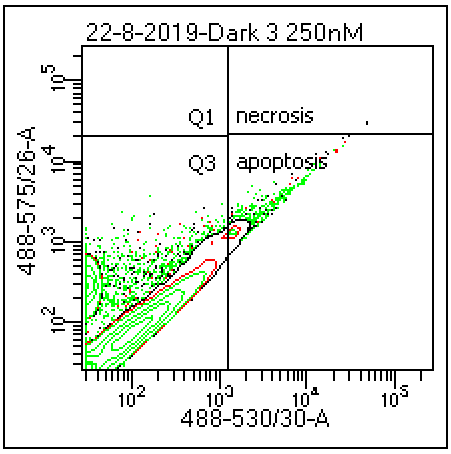


Tube: Dark 3 250nM

| Population | #Events | %Parent | %Total |
|---|---|---|---|
| All Events | 10,000 | #### | 100.0 |
| P1 | 7,812 | 78.1 | 78.1 |
| P2 | 6,586 | 84.3 | 65.9 |
| Q1 | 0 | 0.0 | 0.0 |
| necrosis | 0 | 0.0 | 0.0 |
| Q3 | 6,240 | 94.7 | 62.4 |
| apoptosis | 346 | 5.3 | 3.5 |

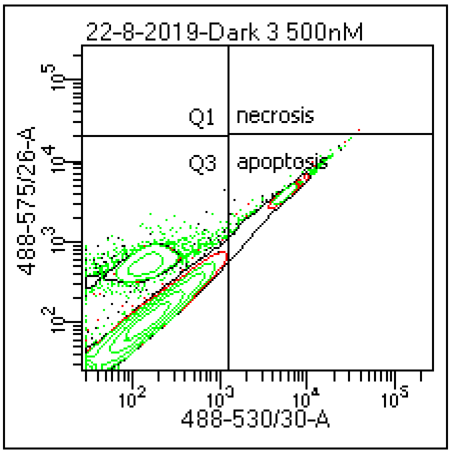


Tube: Dark 3 500nM

| Population | #Events | %Parent | %Total |
|---|---|---|---|
| All Events | 10,000 | #### | 100.0 |
| P1 | 7,067 | 70.7 | 70.7 |
| P2 | 5,948 | 84.2 | 59.5 |
| Q1 | 0 | 0.0 | 0.0 |
| necrosis | 0 | 0.0 | 0.0 |
| Q3 | 5,388 | 90.6 | 53.9 |
| apoptosis | 560 | 9.4 | 5.6 |

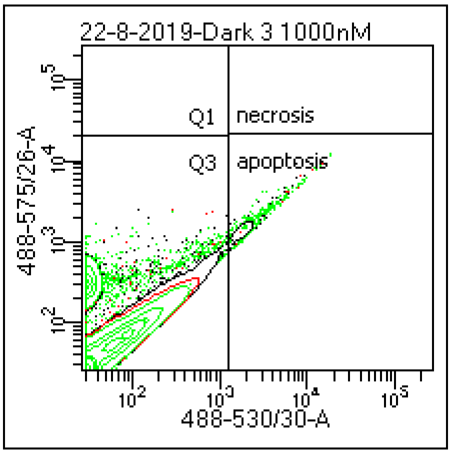


Tube: Dark 3 1000nM

| Population | #Events | %Parent | %Total |
|---|---|---|---|
| All Events | 10,000 | #### | 100.0 |
| P1 | 7,530 | 75.3 | 75.3 |
| P2 | 6,353 | 84.4 | 63.5 |
| Q1 | 0 | 0.0 | 0.0 |
| necrosis | 0 | 0.0 | 0.0 |
| Q3 | 6,068 | 95.5 | 60.7 |
| apoptosis | 285 | 4.5 | 2.8 |

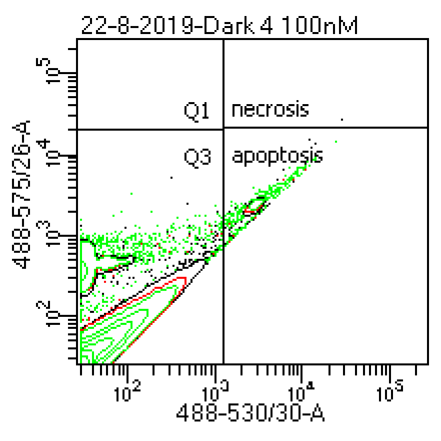


Tube: Dark 4 100nM

| Population | #Events | %Parent | %Total |
|---|---|---|---|
| All Events | 10,000 | #### | 100.0 |
| P1 | 8,124 | 81.2 | 81.2 |
| P2 | 6,723 | 82.8 | 67.2 |
| Q1 | 0 | 0.0 | 0.0 |
| necrosis | 0 | 0.0 | 0.0 |
| Q3 | 6,375 | 94.8 | 63.7 |
| apoptosis | 348 | 5.2 | 3.5 |

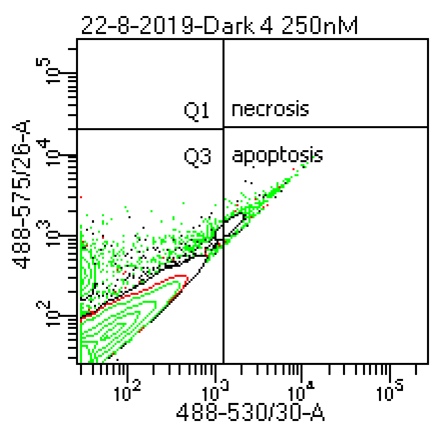


Tube: Dark 4 250nM

| Population | #Events | %Parent | %Total |
|---|---|---|---|
| All Events | 10,000 | #### | 100.0 |
| P1 | 7,313 | 73.1 | 73.1 |
| P2 | 6,264 | 85.7 | 62.6 |
| Q1 | 0 | 0.0 | 0.0 |
| necrosis | 0 | 0.0 | 0.0 |
| Q3 | 5,980 | 95.5 | 59.8 |
| apoptosis | 284 | 4.5 | 2.8 |

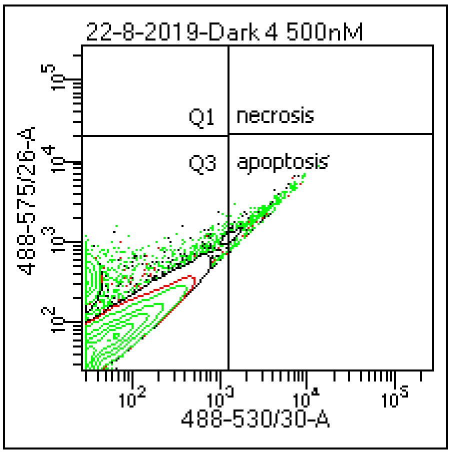


Tube: Dark 4 500nM

| Population | #Events | %Parent | %Total |
|---|---|---|---|
| All Events | 10,000 | #### | 100.0 |
| P1 | 8,121 | 81.2 | 81.2 |
| P2 | 6,880 | 84.7 | 68.8 |
| Q1 | 0 | 0.0 | 0.0 |
| necrosis | 0 | 0.0 | 0.0 |
| Q3 | 6,616 | 96.2 | 66.2 |
| apoptosis | 264 | 3.8 | 2.6 |

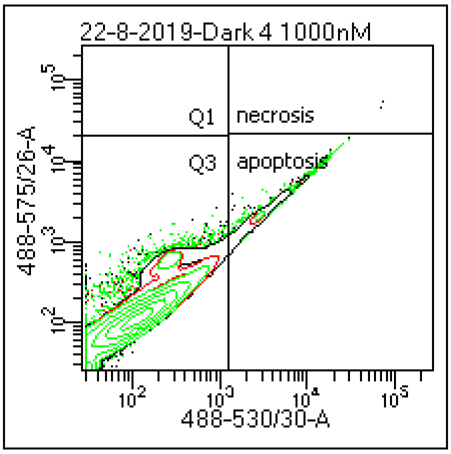


Tube: Dark 4 1000nM

| Population | #Events | %Parent | %Total |
|---|---|---|---|
| All Events | 10,000 | #### | 100.0 |
| P1 | 7,614 | 76.1 | 76.1 |
| P2 | 6,386 | 83.9 | 63.9 |
| Q1 | 0 | 0.0 | 0.0 |
| necrosis | 0 | 0.0 | 0.0 |
| Q3 | 5,778 | 90.5 | 57.8 |
| apoptosis | 608 | 9.5 | 6.1 |

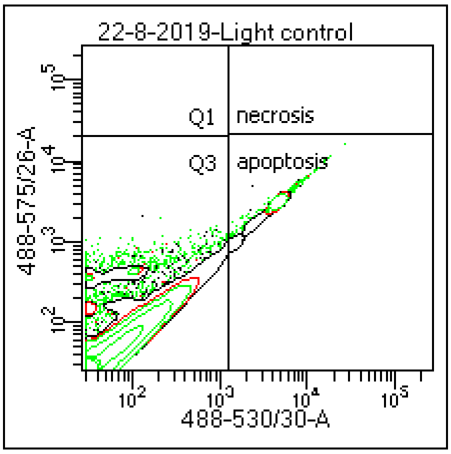


Tube: Light control

| Population | #Events | %Parent | %Total |
|---|---|---|---|
| All Events | 10,000 | #### | 100.0 |
| P1 | 6,692 | 66.9 | 66.9 |
| P2 | 5,662 | 84.6 | 56.6 |
| Q1 | 0 | 0.0 | 0.0 |
| necrosis | 0 | 0.0 | 0.0 |
| Q3 | 5,334 | 94.2 | 53.3 |
| apoptosis | 328 | 5.8 | 3.3 |

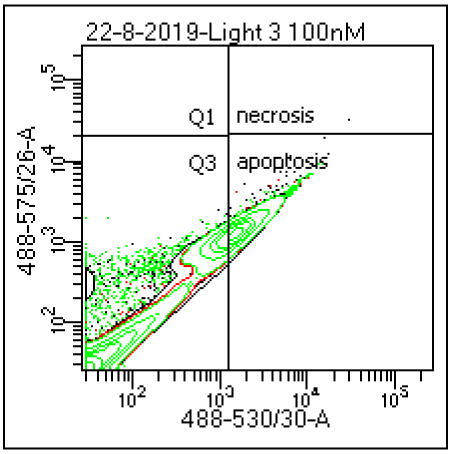


Tube: Light 3 100nM

| Population | #Events | %Parent | %Total |
|---|---|---|---|
| All Events | 10,000 | #### | 100.0 |
| P1 | 7,590 | 75.9 | 75.9 |
| P2 | 6,962 | 91.7 | 69.6 |
| Q1 | 0 | 0.0 | 0.0 |
| necrosis | 0 | 0.0 | 0.0 |
| Q3 | 5,608 | 80.6 | 56.1 |
| apoptosis | 1,354 | 19.4 | 13.5 |

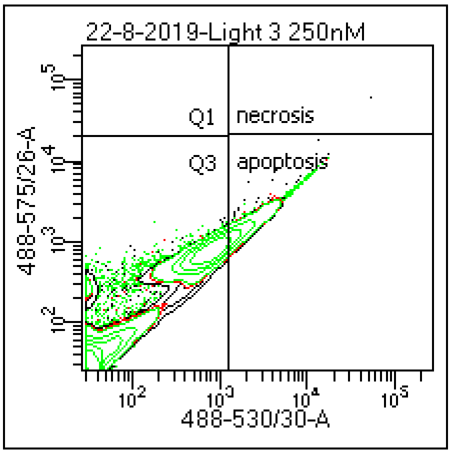


Tube: Light 3 250nM

| Population | #Events | %Parent | %Total |
|---|---|---|---|
| All Events | 10,000 | #### | 100.0 |
| P1 | 7,986 | 79.9 | 79.9 |
| P2 | 7,590 | 95.0 | 75.9 |
| Q1 | 0 | 0.0 | 0.0 |
| necrosis | 0 | 0.0 | 0.0 |
| Q3 | 6,789 | 89.4 | 67.9 |
| apoptosis | 801 | 10.6 | 8.0 |

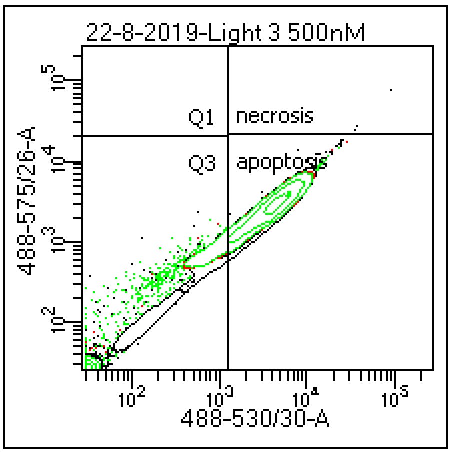


Tube: Light 3 500nM

| Population | #Events | %Parent | %Total |
|---|---|---|---|
| All Events | 10,000 | #### | 100.0 |
| P1 | 2,940 | 29.4 | 29.4 |
| P2 | 2,832 | 96.3 | 28.3 |
| Q1 | 0 | 0.0 | 0.0 |
| necrosis | 0 | 0.0 | 0.0 |
| Q3 | 1,273 | 45.0 | 12.7 |
| apoptosis | 1,559 | 55.0 | 15.6 |

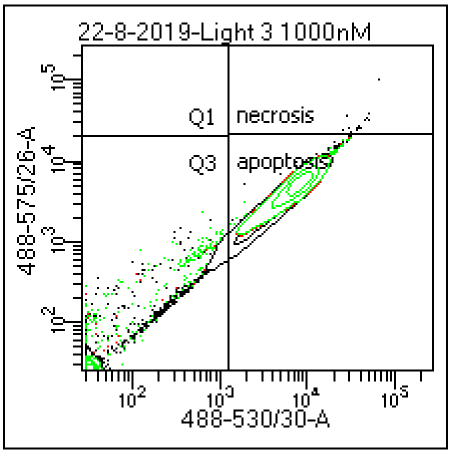


Tube: Light 3 1000nM

| Population | #Events | %Parent | %Total |
|---|---|---|---|
| All Events | 10,000 | #### | 100.0 |
| P1 | 3,976 | 39.8 | 39.8 |
| P2 | 3,656 | 92.0 | 36.6 |
| Q1 | 0 | 0.0 | 0.0 |
| necrosis | 0 | 0.0 | 0.0 |
| Q3 | 1,547 | 42.3 | 15.5 |
| apoptosis | 2,109 | 57.7 | 21.1 |

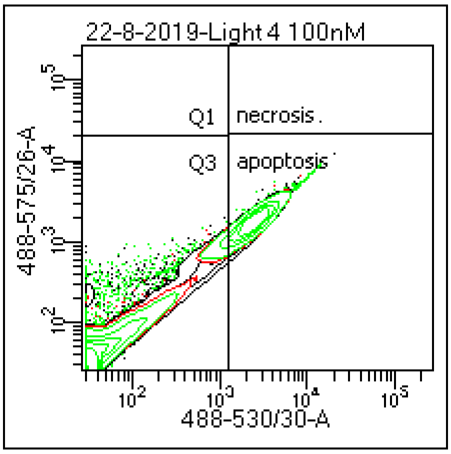


Tube: Light 4 100nM

| Population | #Events | %Parent | %Total |
|---|---|---|---|
| All Events | 10,000 | #### | 100.0 |
| P1 | 7,366 | 73.7 | 73.7 |
| P2 | 6,728 | 91.3 | 67.3 |
| Q1 | 0 | 0.0 | 0.0 |
| necrosis | 0 | 0.0 | 0.0 |
| Q3 | 5,216 | 77.5 | 52.2 |
| apoptosis | 1,512 | 22.5 | 15.1 |

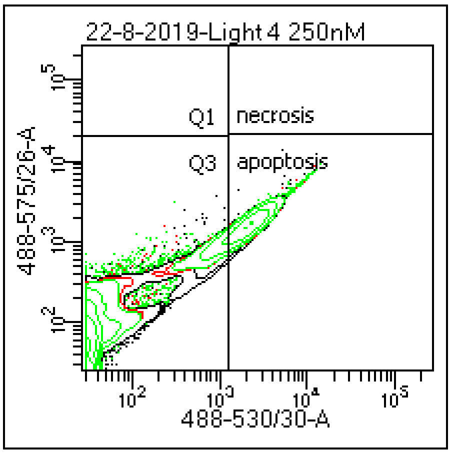


Tube: Light 4 250nM

| Population | #Events | %Parent | %Total |
|---|---|---|---|
| All Events | 10,000 | #### | 100.0 |
| P1 | 6,823 | 68.2 | 68.2 |
| P2 | 6,483 | 95.0 | 64.8 |
| Q1 | 0 | 0.0 | 0.0 |
| necrosis | 0 | 0.0 | 0.0 |
| Q3 | 5,327 | 82.2 | 53.3 |
| apoptosis | 1,156 | 17.8 | 11.6 |

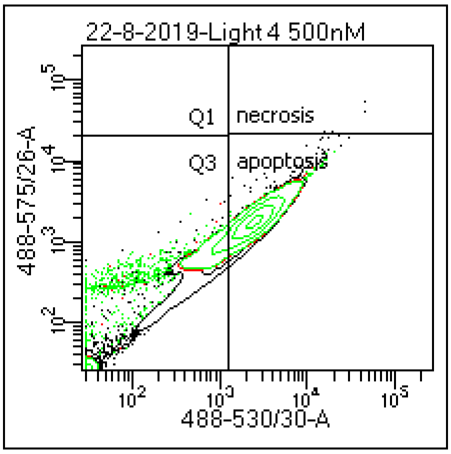


Tube: Light 4 500nM

| Population | #Events | %Parent | %Total |
|---|---|---|---|
| All Events | 10,000 | #### | 100.0 |
| P1 | 4,521 | 45.2 | 45.2 |
| P2 | 4,112 | 91.0 | 41.1 |
| Q1 | 0 | 0.0 | 0.0 |
| necrosis | 0 | 0.0 | 0.0 |
| Q3 | 1,535 | 37.3 | 15.4 |
| apoptosis | 2,577 | 62.7 | 25.8 |

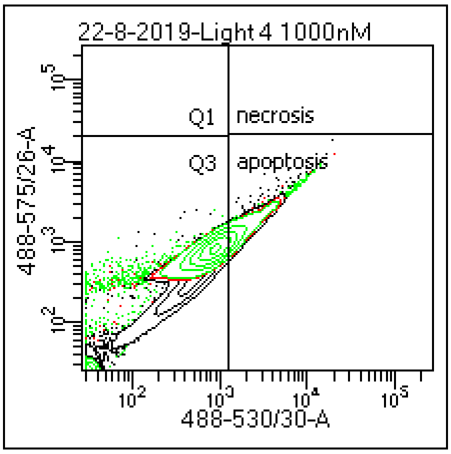


Tube: Light 4 1000nM

| Population | #Events | %Parent | %Total |
|---|---|---|---|
| All Events | 10,000 | #### | 100.0 |
| P1 | 5,101 | 51.0 | 51.0 |
| P2 | 4,465 | 87.5 | 44.6 |
| Q1 | 0 | 0.0 | 0.0 |
| necrosis | 0 | 0.0 | 0.0 |
| Q3 | 3,633 | 81.4 | 36.3 |
| apoptosis | 832 | 18.6 | 8.3 |

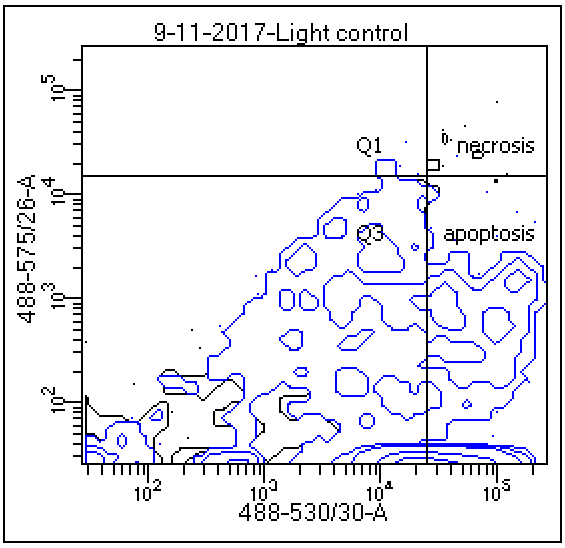


Tube: Light control

| Population | #Events | %Parent | %Total |
|---|---|---|---|
| All Events | 1,957 | #### | 100.0 |
| P1 | 1,471 | 75.2 | 75.2 |
| P2 | 1,227 | 83.4 | 62.7 |
| P3 | 473 | 32.2 | 24.2 |
| Q1 | 6 | 0.4 | 0.3 |
| necrosis | 3 | 0.2 | 0.2 |
| Q3 | 777 | 52.8 | 39.7 |
| apoptosis | 685 | 46.6 | 35.0 |

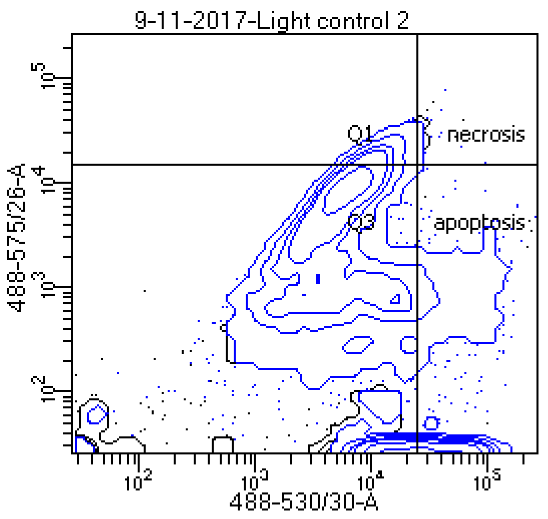


Tube: Light control 2

| Population | #Events | %Parent | %Total |
|---|---|---|---|
| All Events | 3,102 | #### | 100.0 |
| P1 | 2,682 | 86.5 | 86.5 |
| P2 | 1,990 | 74.2 | 64.2 |
| P3 | 1,792 | 66.8 | 57.8 |
| Q1 | 141 | 5.3 | 4.5 |
| necrosis | 15 | 0.6 | 0.5 |
| Q3 | 1,880 | 70.1 | 60.6 |
| apoptosis | 646 | 24.1 | 20.8 |

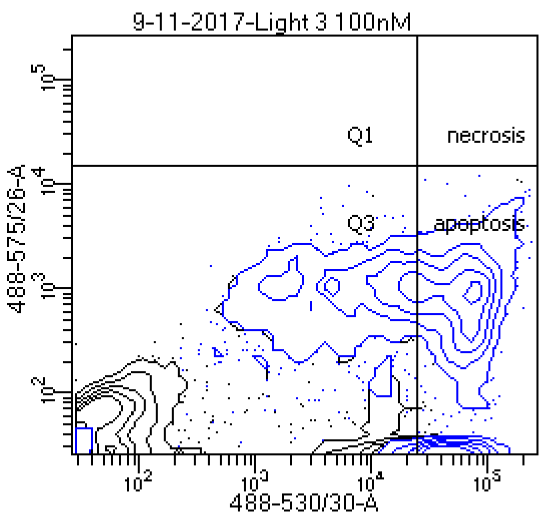


Tube: Light 3 100nM

| Population | #Events | %Parent | %Total |
|---|---|---|---|
| All Events | 3,400 | #### | 100.0 |
| P1 | 1,963 | 57.7 | 57.7 |
| P2 | 1,609 | 82.0 | 47.3 |
| P3 | 1,465 | 74.6 | 43.1 |
| Q1 | 0 | 0.0 | 0.0 |
| necrosis | 0 | 0.0 | 0.0 |
| Q3 | 833 | 42.4 | 24.5 |
| apoptosis | 1,130 | 57.6 | 33.2 |

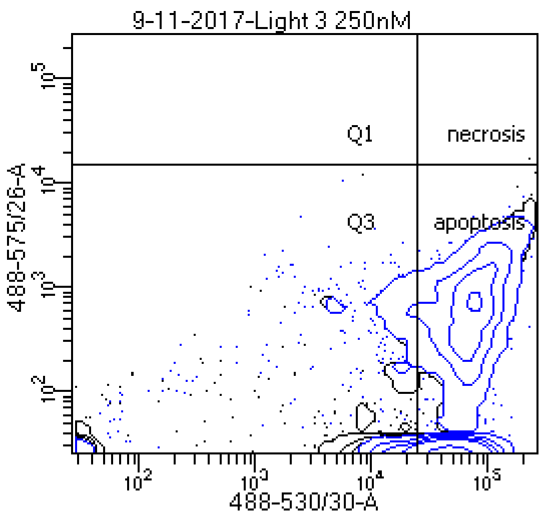


Tube: Light 3 250nM

| Population | #Events | %Parent | %Total |
|---|---|---|---|
| All Events | 3,103 | #### | 100.0 |
| P1 | 2,143 | 69.1 | 69.1 |
| P2 | 2,034 | 94.9 | 65.5 |
| P3 | 1,035 | 48.3 | 33.4 |
| Q1 | 0 | 0.0 | 0.0 |
| necrosis | 0 | 0.0 | 0.0 |
| Q3 | 394 | 18.4 | 12.7 |
| apoptosis | 1,749 | 81.6 | 56.4 |

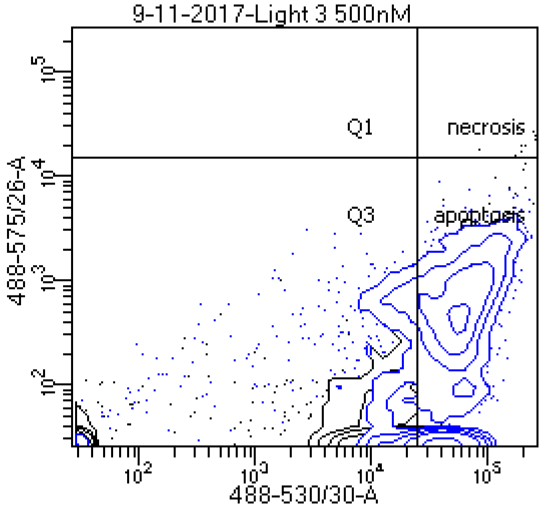


Tube: Light 3 500nM

| Population | #Events | %Parent | %Total |
|---|---|---|---|
| All Events | 3,185 | #### | 100.0 |
| P1 | 1,958 | 61.5 | 61.5 |
| P2 | 1,845 | 94.2 | 57.9 |
| P3 | 949 | 48.5 | 29.8 |
| Q1 | 0 | 0.0 | 0.0 |
| necrosis | 0 | 0.0 | 0.0 |
| Q3 | 462 | 23.6 | 14.5 |
| apoptosis | 1,496 | 76.4 | 47.0 |

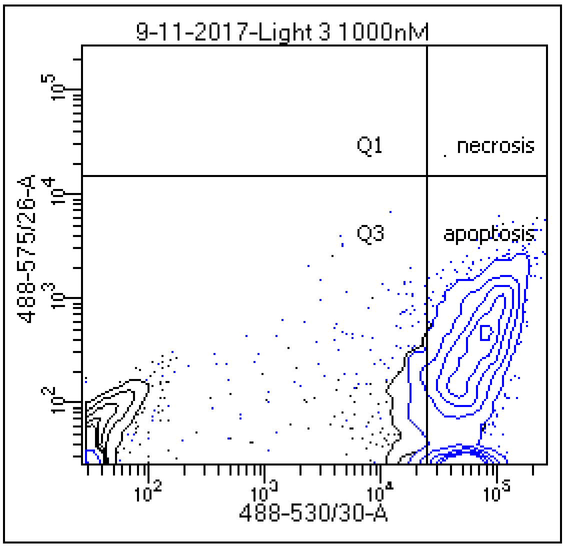


Tube: Light 3 1000nM

| Population | #Events | %Parent | %Total |
|---|---|---|---|
| All Events | 3,175 | #### | 100.0 |
| P1 | 1,842 | 58.0 | 58.0 |
| P2 | 1,734 | 94.1 | 54.6 |
| P3 | 941 | 51.1 | 29.6 |
| Q1 | 0 | 0.0 | 0.0 |
| necrosis | 2 | 0.1 | 0.1 |
| Q3 | 271 | 14.7 | 8.5 |
| apoptosis | 1,569 | 85.2 | 49.4 |

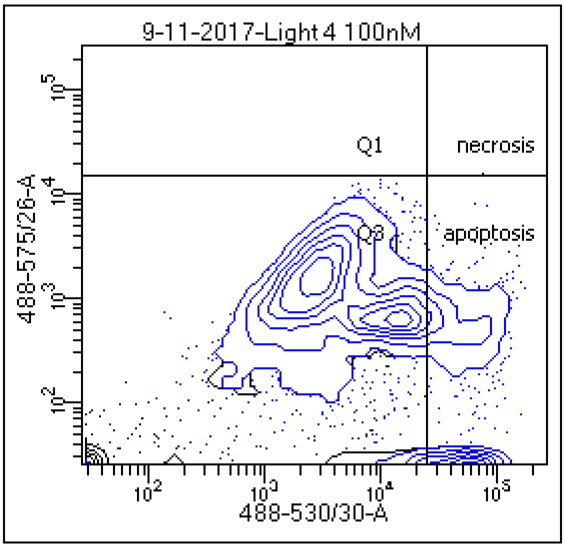


Tube: Light 4 100nM

| Population | #Events | %Parent | %Total |
|---|---|---|---|
| All Events | 3,455 | #### | 100.0 |
| P1 | 3,140 | 90.9 | 90.9 |
| P2 | 1,811 | 57.7 | 52.4 |
| P3 | 2,587 | 82.4 | 74.9 |
| Q1 | 0 | 0.0 | 0.0 |
| necrosis | 0 | 0.0 | 0.0 |
| Q3 | 2,485 | 79.1 | 71.9 |
| apoptosis | 655 | 20.9 | 19.0 |

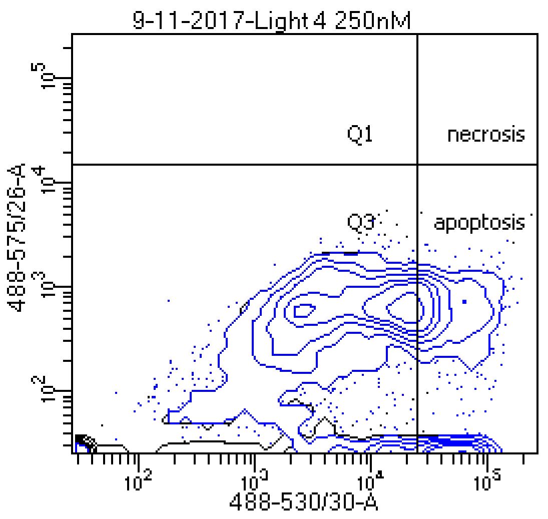


Tube: Light 4 250nM

| Population | #Events | %Parent | %Total |
|---|---|---|---|
| All Events | 3,073 | #### | 100.0 |
| P1 | 2,660 | 86.6 | 86.6 |
| P2 | 1,782 | 67.0 | 58.0 |
| P3 | 1,846 | 69.4 | 60.1 |
| Q1 | 0 | 0.0 | 0.0 |
| necrosis | 0 | 0.0 | 0.0 |
| Q3 | 1,852 | 69.6 | 60.3 |
| apoptosis | 808 | 30.4 | 26.3 |

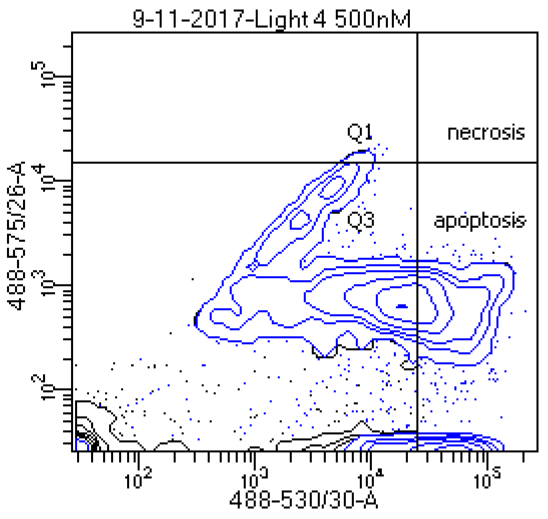


Tube: Light 4 500nM

| Population | #Events | %Parent | %Total |
|---|---|---|---|
| All Events | 3,493 | #### | 100.0 |
| P1 | 2,560 | 73.3 | 73.3 |
| P2 | 1,917 | 74.9 | 54.9 |
| P3 | 2,000 | 78.1 | 57.3 |
| Q1 | 25 | 1.0 | 0.7 |
| necrosis | 0 | 0.0 | 0.0 |
| Q3 | 1,567 | 61.2 | 44.9 |
| apoptosis | 968 | 37.8 | 27.7 |

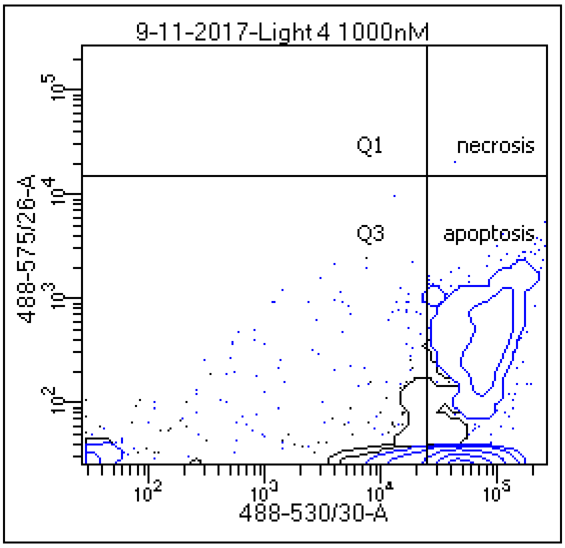


Tube: Light 4 1000nM

| Population | #Events | %Parent | %Total |
|---|---|---|---|
| All Events | 2,446 | #### | 100.0 |
| P1 | 1,497 | 61.2 | 61.2 |
| P2 | 1,373 | 91.7 | 56.1 |
| P3 | 409 | 27.3 | 16.7 |
| Q1 | 0 | 0.0 | 0.0 |
| necrosis | 2 | 0.1 | 0.1 |
| Q3 | 241 | 16.1 | 9.9 |
| apoptosis | 1,254 | 83.8 | 51.3 |

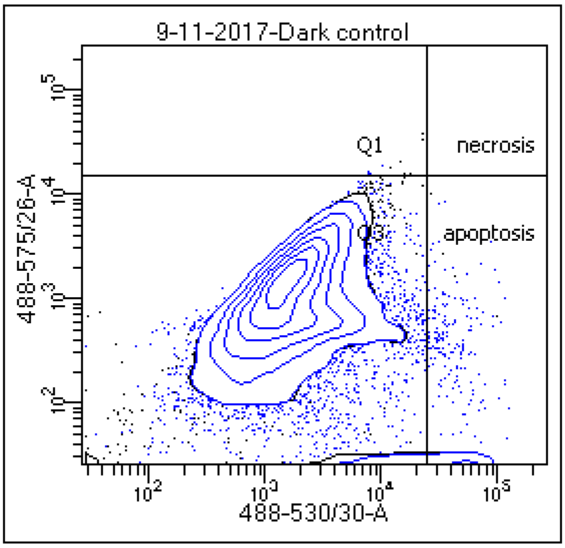


Tube: Dark control

| Population | #Events | %Parent | %Total |
|---|---|---|---|
| All Events | 10,000 | #### | 100.0 |
| P1 | 9,491 | 94.9 | 94.9 |
| P2 | 1,929 | 20.3 | 19.3 |
| P3 | 8,030 | 84.6 | 80.3 |
| Q1 | 4 | 0.0 | 0.0 |
| necrosis | 0 | 0.0 | 0.0 |
| Q3 | 9,050 | 95.4 | 90.5 |
| apoptosis | 437 | 4.6 | 4.4 |

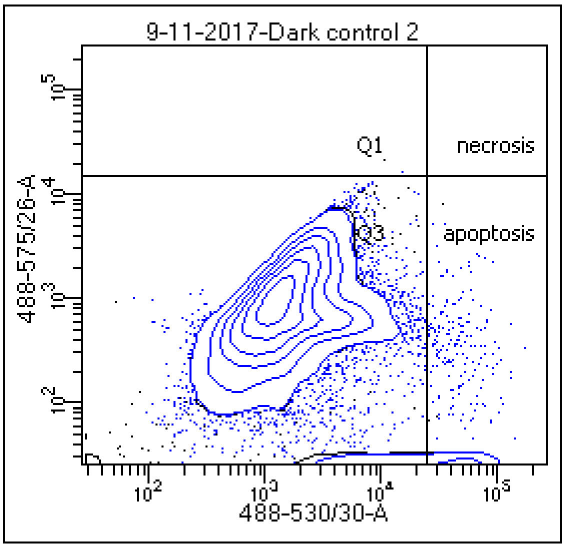


Tube: Dark control 2

| Population | #Events | %Parent | %Total |
|---|---|---|---|
| All Events | 10,000 | #### | 100.0 |
| P1 | 9,533 | 95.3 | 95.3 |
| P2 | 1,908 | 20.0 | 19.1 |
| P3 | 7,745 | 81.2 | 77.4 |
| Q1 | 1 | 0.0 | 0.0 |
| necrosis | 0 | 0.0 | 0.0 |
| Q3 | 9,017 | 94.6 | 90.2 |
| apoptosis | 515 | 5.4 | 5.1 |

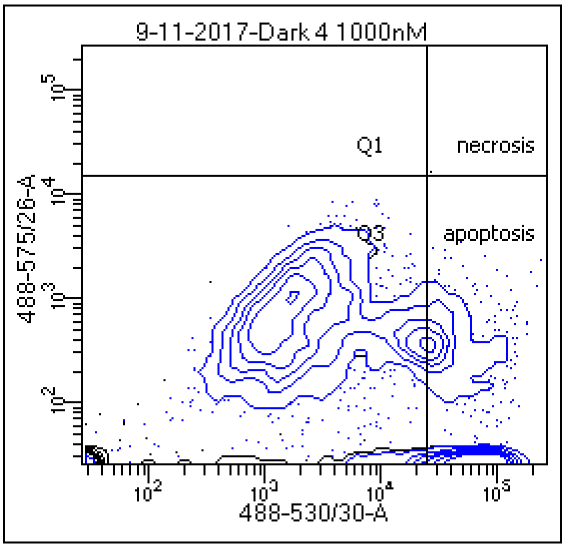


Tube: Dark 4 1000nM

| Population | #Events | %Parent | %Total |
|---|---|---|---|
| All Events | 3,049 | #### | 100.0 |
| P1 | 2,784 | 91.3 | 91.3 |
| P2 | 1,346 | 48.3 | 44.1 |
| P3 | 1,900 | 68.2 | 62.3 |
| Q1 | 0 | 0.0 | 0.0 |
| necrosis | 0 | 0.0 | 0.0 |
| Q3 | 1,974 | 70.9 | 64.7 |
| apoptosis | 810 | 29.1 | 26.6 |

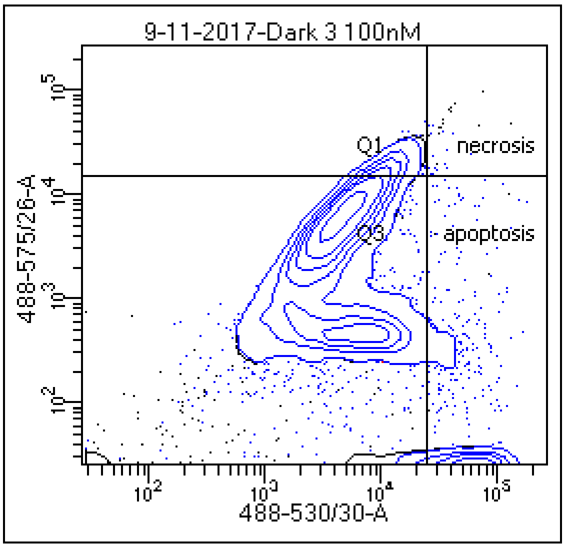


Tube: Dark 3 100nM

| Population | #Events | %Parent | %Total |
|---|---|---|---|
| All Events | 5,086 | #### | 100.0 |
| P1 | 4,758 | 93.6 | 93.6 |
| P2 | 2,906 | 61.1 | 57.1 |
| P3 | 4,076 | 85.7 | 80.1 |
| Q1 | 272 | 5.7 | 5.3 |
| necrosis | 18 | 0.4 | 0.4 |
| Q3 | 3,856 | 81.0 | 75.8 |
| apoptosis | 612 | 12.9 | 12.0 |

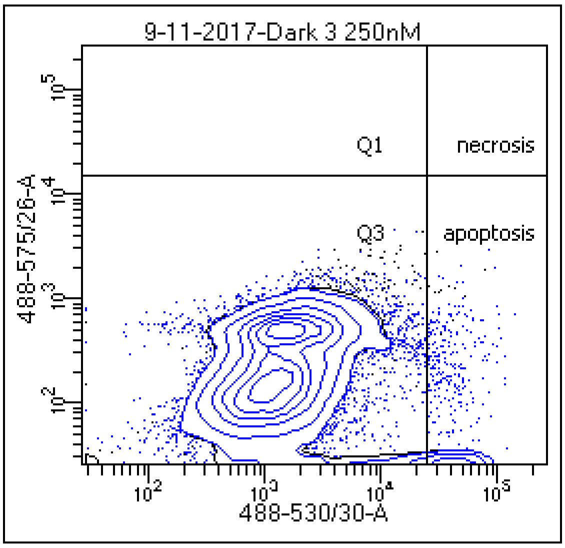


Tube: Dark 3 250nM

| Population | #Events | %Parent | %Total |
|---|---|---|---|
| All Events | 10,000 | #### | 100.0 |
| P1 | 9,437 | 94.4 | 94.4 |
| P2 | 1,784 | 18.9 | 17.8 |
| P3 | 4,354 | 46.1 | 43.5 |
| Q1 | 0 | 0.0 | 0.0 |
| necrosis | 0 | 0.0 | 0.0 |
| Q3 | 8,738 | 92.6 | 87.4 |
| apoptosis | 699 | 7.4 | 7.0 |

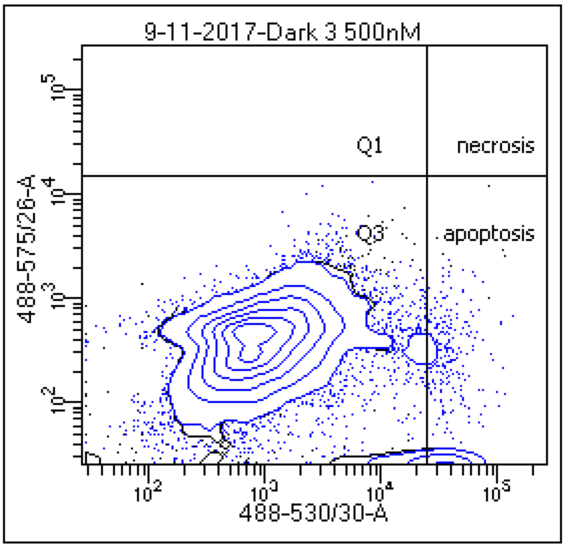


Tube: Dark 3 500nM

| Population | #Events | %Parent | %Total |
|---|---|---|---|
| All Events | 10,000 | #### | 100.0 |
| P1 | 9,499 | 95.0 | 95.0 |
| P2 | 1,403 | 14.8 | 14.0 |
| P3 | 6,881 | 72.4 | 68.8 |
| Q1 | 0 | 0.0 | 0.0 |
| necrosis | 0 | 0.0 | 0.0 |
| Q3 | 8,911 | 93.8 | 89.1 |
| apoptosis | 588 | 6.2 | 5.9 |

Tube: Dark 3 1000nM

| Population | #Events | %Parent | %Total |
|---|---|---|---|
| All Events | 10,000 | #### | 100.0 |
| P1 | 9,462 | 94.6 | 94.6 |
| P2 | 3,649 | 38.6 | 36.5 |
| P3 | 7,921 | 83.7 | 79.2 |
| Q1 | 0 | 0.0 | 0.0 |
| necrosis | 0 | 0.0 | 0.0 |
| Q3 | 8,342 | 88.2 | 83.4 |
| apoptosis | 1,120 | 11.8 | 11.2 |

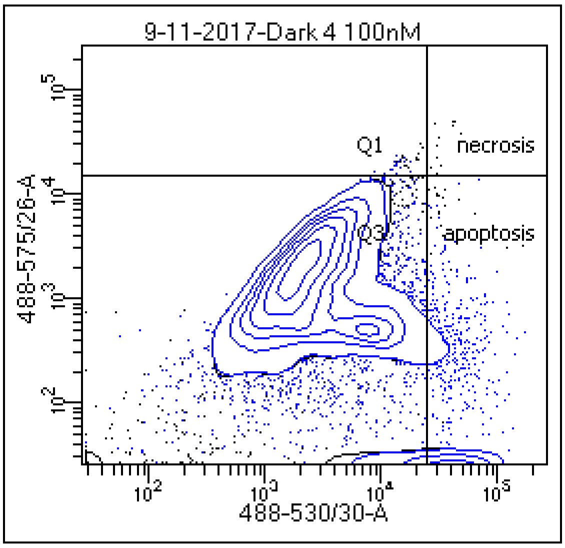

Tube: Dark 4 100nM

| Population | #Events | %Parent | %Total |
|---|---|---|---|
| All Events | 10,000 | #### | 100.0 |
| P1 | 9,380 | 93.8 | 93.8 |
| P2 | 3,567 | 38.0 | 35.7 |
| P3 | 8,120 | 86.6 | 81.2 |
| Q1 | 25 | 0.3 | 0.2 |
| necrosis | 0 | 0.0 | 0.0 |
| Q3 | 8,540 | 91.0 | 85.4 |
| apoptosis | 815 | 8.7 | 8.2 |

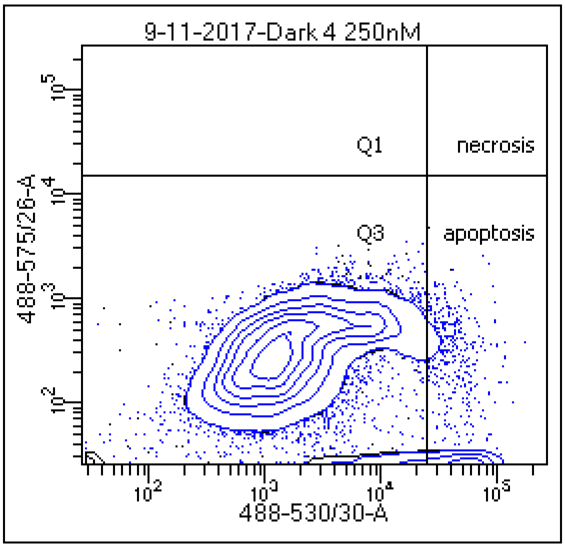


Tube: Dark 4 250nM

| Population | #Events | %Parent | %Total |
|---|---|---|---|
| All Events | 10,000 | #### | 100.0 |
| P1 | 9,473 | 94.7 | 94.7 |
| P2 | 2,642 | 27.9 | 26.4 |
| P3 | 5,684 | 60.0 | 56.8 |
| Q1 | 0 | 0.0 | 0.0 |
| necrosis | 0 | 0.0 | 0.0 |
| Q3 | 8,601 | 90.8 | 86.0 |
| apoptosis | 872 | 9.2 | 8.7 |

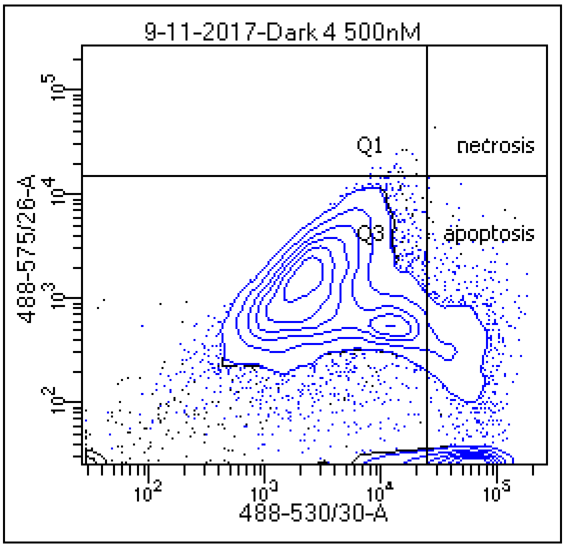


Tube: Dark 4 500nM

| Population | #Events | %Parent | %Total |
|---|---|---|---|
| All Events | 10,000 | #### | 100.0 |
| P1 | 9,324 | 93.2 | 93.2 |
| P2 | 4,755 | 51.0 | 47.6 |
| P3 | 7,609 | 81.6 | 76.1 |
| Q1 | 21 | 0.2 | 0.2 |
| necrosis | 1 | 0.0 | 0.0 |
| Q3 | 7,472 | 80.1 | 74.7 |
| apoptosis | 1,830 | 19.6 | 18.3 |

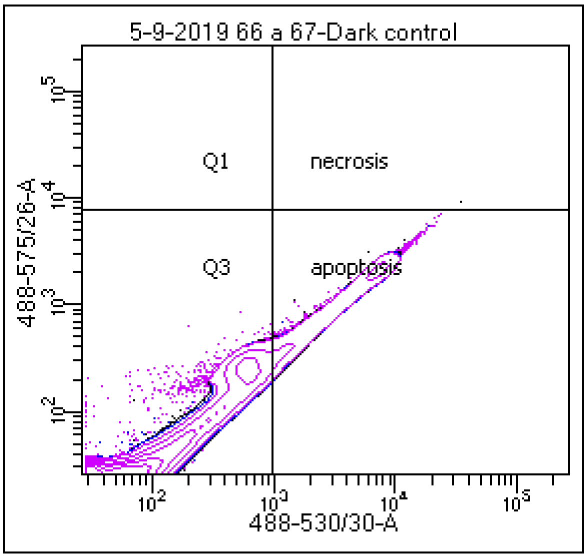


Tube: Dark control

| Population | #Events | %Parent | %Total |
|---|---|---|---|
| All Events | 10,000 | #### | 100.0 |
| P1 | 9,357 | 93.6 | 93.6 |
| P2 | 8,015 | 85.7 | 80.2 |
| Q1 | 0 | 0.0 | 0.0 |
| necrosis | 0 | 0.0 | 0.0 |
| Q3 | 6,293 | 78.5 | 62.9 |
| apoptosis | 1,722 | 21.5 | 17.2 |

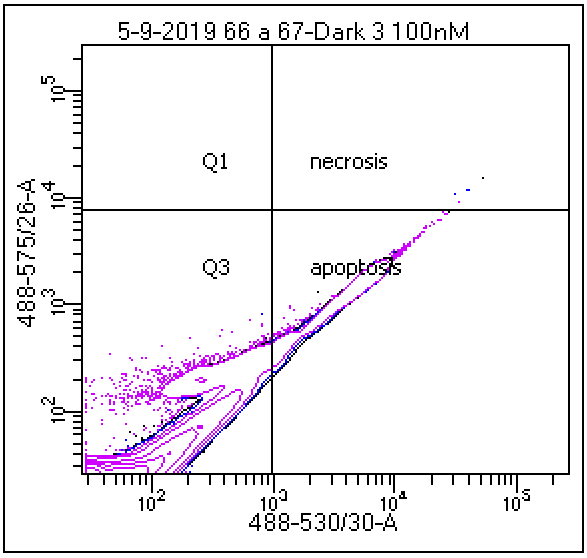


Tube: Dark 3 100nM

| Population | #Events | %Parent | %Total |
|---|---|---|---|
| All Events | 10,000 | #### | 100.0 |
| P1 | 9,242 | 92.4 | 92.4 |
| P2 | 7,751 | 83.9 | 77.5 |
| Q1 | 0 | 0.0 | 0.0 |
| necrosis | 1 | 0.0 | 0.0 |
| Q3 | 6,703 | 86.5 | 67.0 |
| apoptosis | 1,047 | 13.5 | 10.5 |

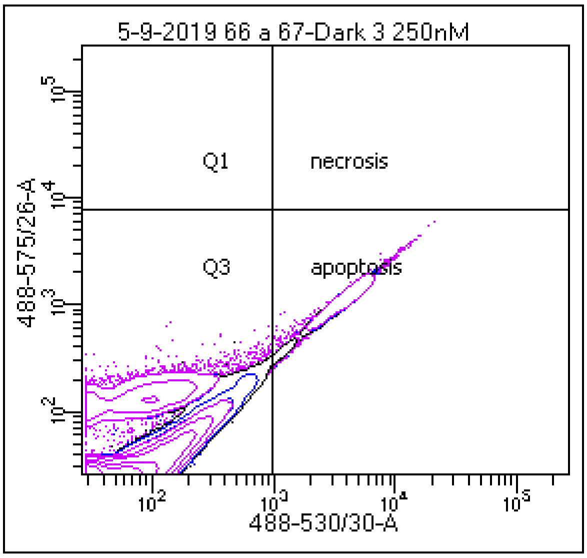


Tube: Dark 3 250nM

| Population | #Events | %Parent | %Total |
|---|---|---|---|
| All Events | 10,000 | #### | 100.0 |
| P1 | 9,243 | 92.4 | 92.4 |
| P2 | 7,759 | 83.9 | 77.6 |
| Q1 | 0 | 0.0 | 0.0 |
| necrosis | 0 | 0.0 | 0.0 |
| Q3 | 7,053 | 90.9 | 70.5 |
| apoptosis | 706 | 9.1 | 7.1 |

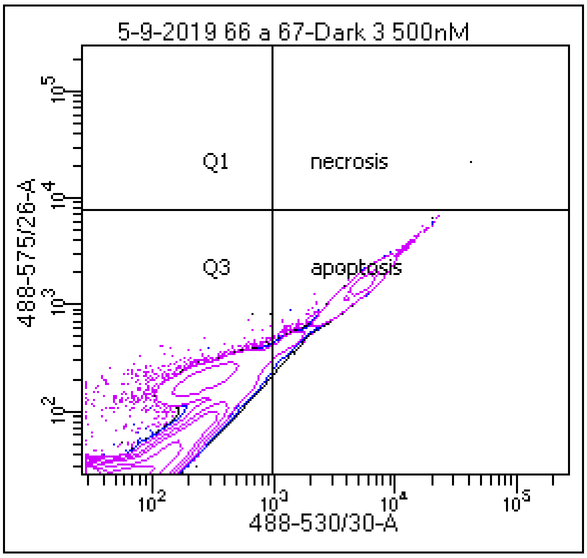


Tube: Dark 3 500nM

| Population | #Events | %Parent | %Total |
|---|---|---|---|
| All Events | 10,000 | #### | 100.0 |
| P1 | 9,320 | 93.2 | 93.2 |
| P2 | 7,958 | 85.4 | 79.6 |
| Q1 | 0 | 0.0 | 0.0 |
| necrosis | 0 | 0.0 | 0.0 |
| Q3 | 6,751 | 84.8 | 67.5 |
| apoptosis | 1,207 | 15.2 | 12.1 |

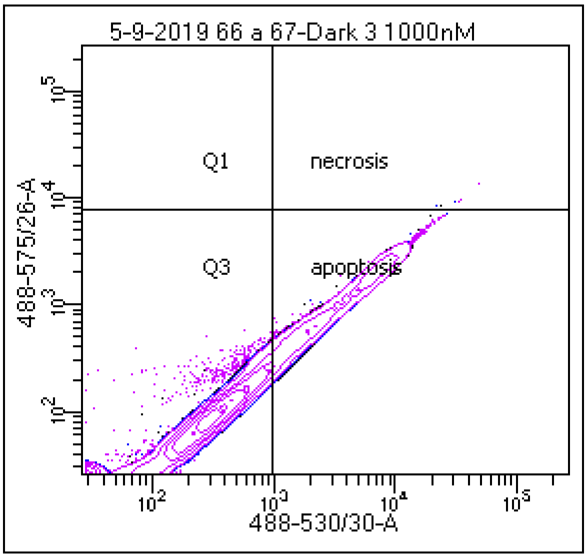


Tube: Dark 3 1000nM

| Population | #Events | %Parent | %Total |
|---|---|---|---|
| All Events | 10,000 | #### | 100.0 |
| P1 | 9,454 | 94.5 | 94.5 |
| P2 | 8,186 | 86.6 | 81.9 |
| Q1 | 0 | 0.0 | 0.0 |
| necrosis | 3 | 0.0 | 0.0 |
| Q3 | 5,389 | 65.8 | 53.9 |
| apoptosis | 2,794 | 34.1 | 27.9 |

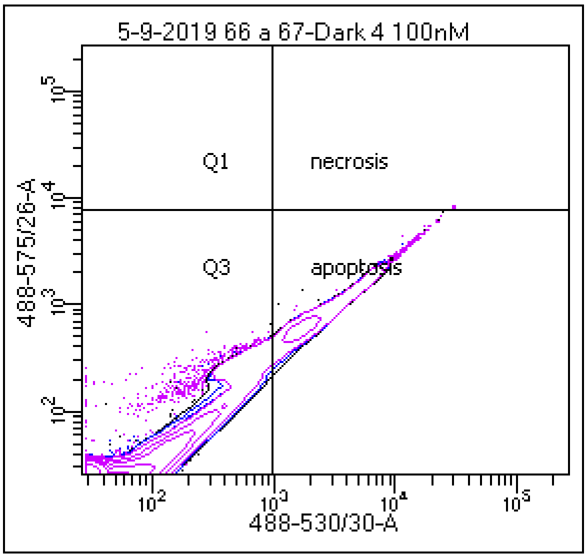


Tube: Dark 4 100nM

| Population | #Events | %Parent | %Total |
|---|---|---|---|
| All Events | 10,000 | #### | 100.0 |
| P1 | 9,152 | 91.5 | 91.5 |
| P2 | 7,743 | 84.6 | 77.4 |
| Q1 | 0 | 0.0 | 0.0 |
| necrosis | 4 | 0.1 | 0.0 |
| Q3 | 6,362 | 82.2 | 63.6 |
| apoptosis | 1,377 | 17.8 | 13.8 |

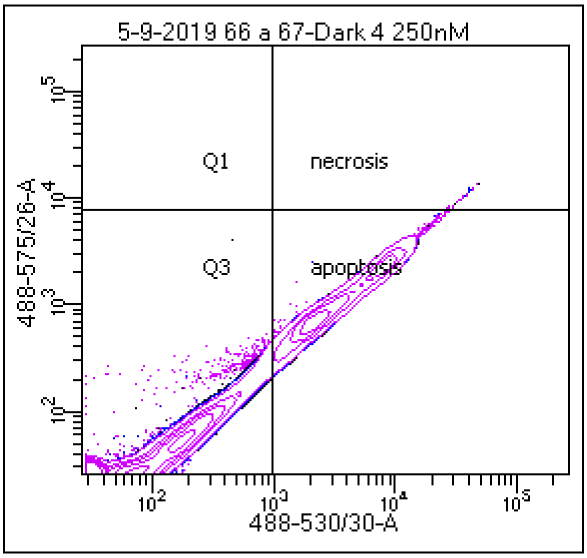


Tube: Dark 4 250nM

| Population | #Events | %Parent | %Total |
|---|---|---|---|
| All Events | 10,000 | #### | 100.0 |
| P1 | 9,723 | 97.2 | 97.2 |
| P2 | 8,840 | 90.9 | 88.4 |
| Q1 | 0 | 0.0 | 0.0 |
| necrosis | 32 | 0.4 | 0.3 |
| Q3 | 5,781 | 65.4 | 57.8 |
| apoptosis | 3,027 | 34.2 | 30.3 |

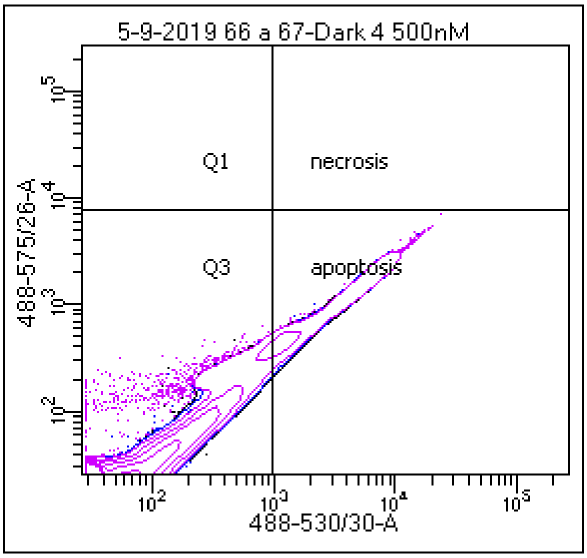


Tube: Dark 4 500nM

| Population | #Events | %Parent | %Total |
|---|---|---|---|
| All Events | 10,000 | #### | 100.0 |
| P1 | 9,313 | 93.1 | 93.1 |
| P2 | 7,936 | 85.2 | 79.4 |
| Q1 | 0 | 0.0 | 0.0 |
| necrosis | 1 | 0.0 | 0.0 |
| Q3 | 6,547 | 82.5 | 65.5 |
| apoptosis | 1,388 | 17.5 | 13.9 |

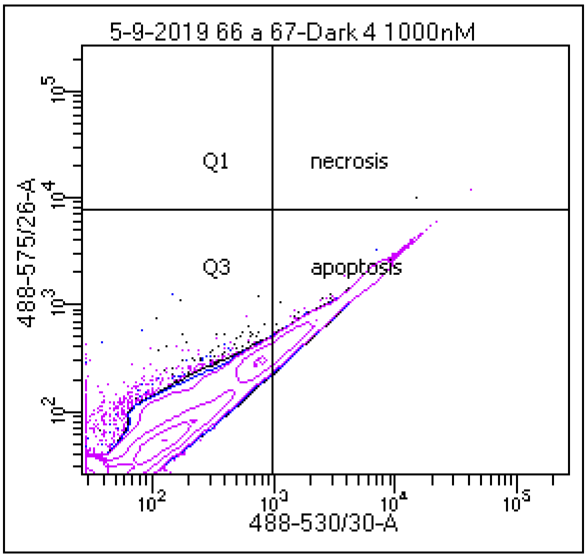


Tube: Dark 4 1000nM

| Population | #Events | %Parent | %Total |
|---|---|---|---|
| All Events | 10,000 | #### | 100.0 |
| P1 | 9,425 | 94.2 | 94.2 |
| P2 | 8,265 | 87.7 | 82.6 |
| Q1 | 0 | 0.0 | 0.0 |
| necrosis | 1 | 0.0 | 0.0 |
| Q3 | 7,044 | 85.2 | 70.4 |
| apoptosis | 1,220 | 14.8 | 12.2 |

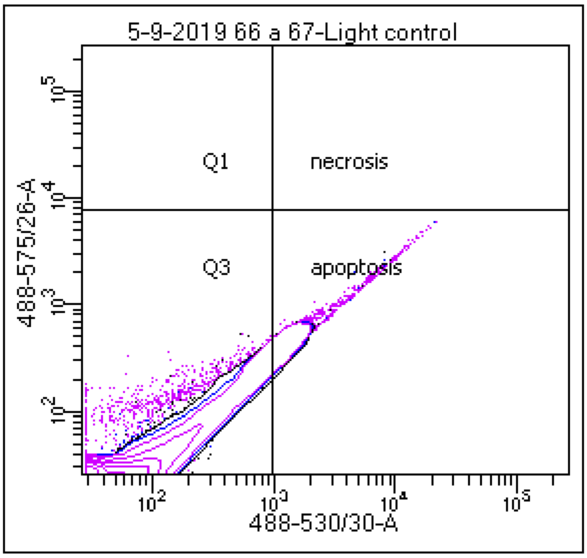


Tube: Light control

| Population | #Events | %Parent | %Total |
|---|---|---|---|
| All Events | 10,000 | #### | 100.0 |
| P1 | 8,678 | 86.8 | 86.8 |
| P2 | 6,921 | 79.8 | 69.2 |
| Q1 | 0 | 0.0 | 0.0 |
| necrosis | 0 | 0.0 | 0.0 |
| Q3 | 6,175 | 89.2 | 61.8 |
| apoptosis | 746 | 10.8 | 7.5 |

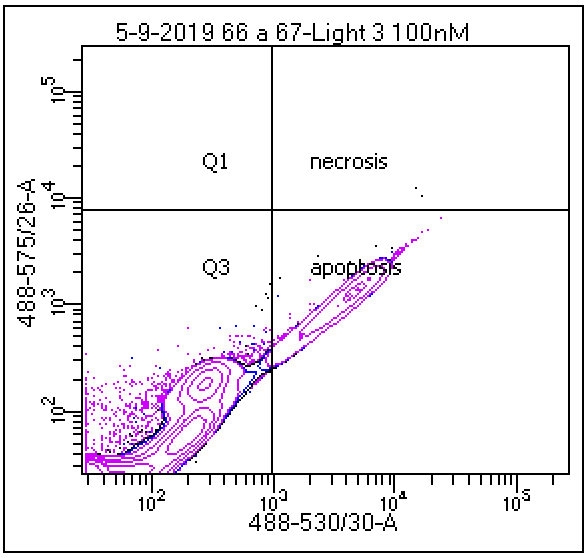


Tube: Light 3 100nM

| Population | #Events | %Parent | %Total |
|---|---|---|---|
| All Events | 10,000 | #### | 100.0 |
| P1 | 9,620 | 96.2 | 96.2 |
| P2 | 8,904 | 92.6 | 89.0 |
| Q1 | 0 | 0.0 | 0.0 |
| necrosis | 0 | 0.0 | 0.0 |
| Q3 | 7,337 | 82.4 | 73.4 |
| apoptosis | 1,567 | 17.6 | 15.7 |

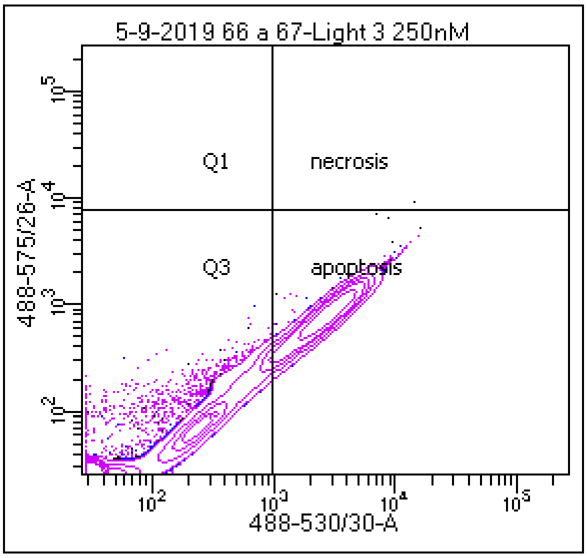


Tube: Light 3 250nM

| Population | #Events | %Parent | %Total |
|---|---|---|---|
| All Events | 10,000 | #### | 100.0 |
| P1 | 9,935 | 99.4 | 99.4 |
| P2 | 9,623 | 96.9 | 96.2 |
| Q1 | 0 | 0.0 | 0.0 |
| necrosis | 0 | 0.0 | 0.0 |
| Q3 | 6,203 | 64.5 | 62.0 |
| apoptosis | 3,420 | 35.5 | 34.2 |

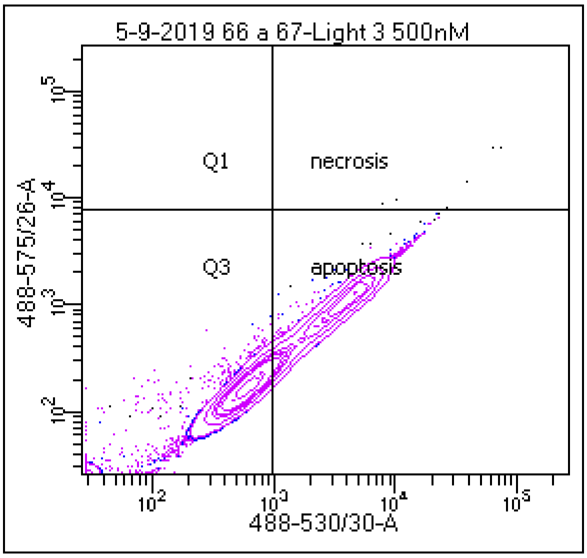


Tube: Light 3 500nM

| Population | #Events | %Parent | %Total |
|---|---|---|---|
| All Events | 10,000 | #### | 100.0 |
| P1 | 9,968 | 99.7 | 99.7 |
| P2 | 9,542 | 95.7 | 95.4 |
| Q1 | 0 | 0.0 | 0.0 |
| necrosis | 0 | 0.0 | 0.0 |
| Q3 | 4,919 | 51.6 | 49.2 |
| apoptosis | 4,623 | 48.4 | 46.2 |

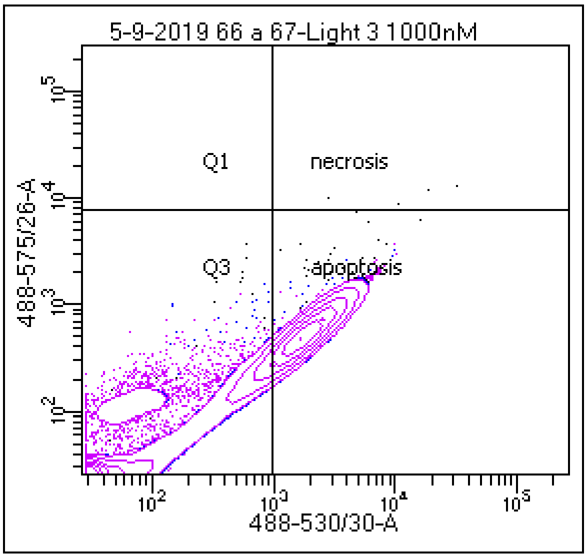


Tube: Light 3 1000nM

| Population | #Events | %Parent | %Total |
|---|---|---|---|
| All Events | 10,000 | #### | 100.0 |
| P1 | 9,939 | 99.4 | 99.4 |
| P2 | 9,420 | 94.8 | 94.2 |
| Q1 | 0 | 0.0 | 0.0 |
| necrosis | 0 | 0.0 | 0.0 |
| Q3 | 5,308 | 56.3 | 53.1 |
| apoptosis | 4,112 | 43.7 | 41.1 |

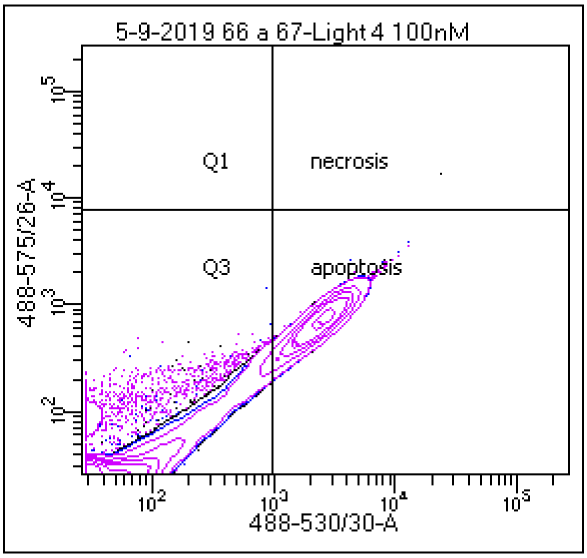


Tube: Light 4 100nM

| Population | #Events | %Parent | %Total |
|---|---|---|---|
| All Events | 10,000 | #### | 100.0 |
| P1 | 9,415 | 94.2 | 94.2 |
| P2 | 8,327 | 88.4 | 83.3 |
| Q1 | 0 | 0.0 | 0.0 |
| necrosis | 0 | 0.0 | 0.0 |
| Q3 | 5,860 | 70.4 | 58.6 |
| apoptosis | 2,467 | 29.6 | 24.7 |

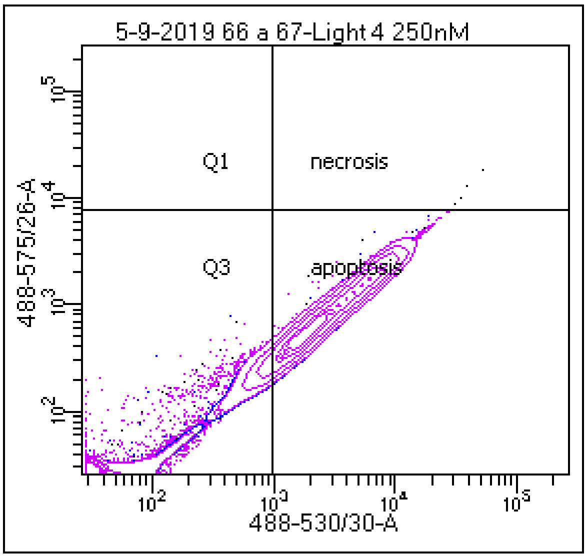


Tube: Light 4 250nM

| Population | #Events | %Parent | %Total |
|---|---|---|---|
| All Events | 10,000 | #### | 100.0 |
| P1 | 9,954 | 99.5 | 99.5 |
| P2 | 9,613 | 96.6 | 96.1 |
| Q1 | 0 | 0.0 | 0.0 |
| necrosis | 2 | 0.0 | 0.0 |
| Q3 | 4,339 | 45.1 | 43.4 |
| apoptosis | 5,272 | 54.8 | 52.7 |

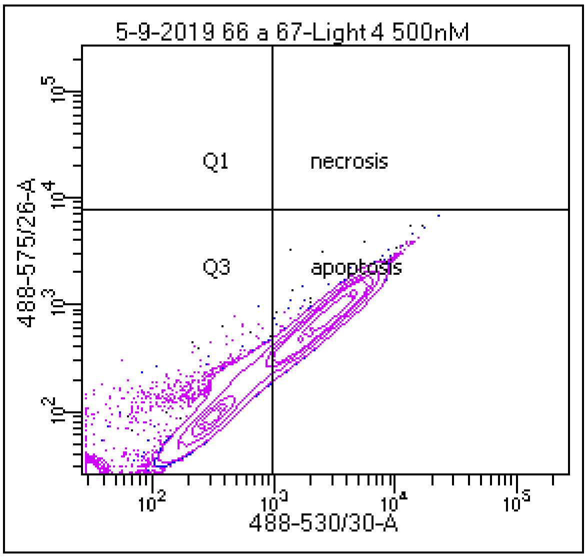


Tube: Light 4 500nM

| Population | #Events | %Parent | %Total |
|---|---|---|---|
| All Events | 10,000 | #### | 100.0 |
| P1 | 9,961 | 99.6 | 99.6 |
| P2 | 9,492 | 95.3 | 94.9 |
| Q1 | 0 | 0.0 | 0.0 |
| necrosis | 0 | 0.0 | 0.0 |
| Q3 | 5,527 | 58.2 | 55.3 |
| apoptosis | 3,965 | 41.8 | 39.6 |

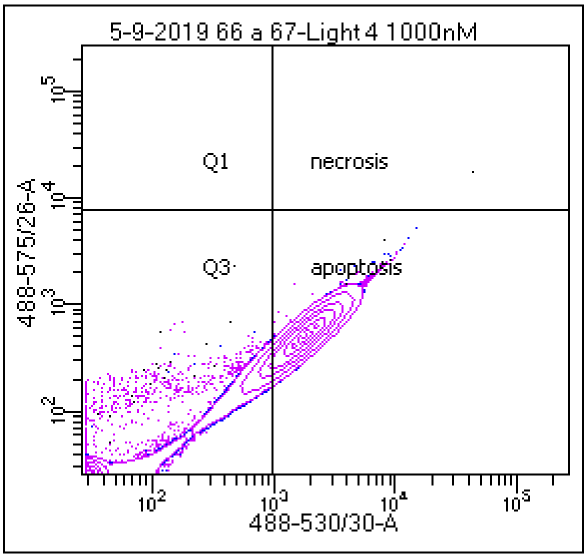


Tube: Light 4 1000nM

| Population | #Events | %Parent | %Total |
| --- | --- | --- | --- |
| All Events | 10,000 | #### | 100.0 |
| P1 | 9,964 | 99.6 | 99.6 |
| P2 | 9,441 | 94.8 | 94.4 |
| Q1 | 0 | 0.0 | 0.0 |
| necrosis | 0 | 0.0 | 0.0 |
| Q3 | 4,296 | 45.5 | 43.0 |
| apoptosis | 5,145 | 54.5 | 51.4 |

**References**

1. Bříza, T.; Králová, J.; Rimpelová, S.; Havlík, M.; Kaplánek, R.; Kejík, Z.; Reddy, B.; Záruba, K.; Ruml, T.; Mikula, I.; Martásek, P.; Král, V. Dimethinium heteroaromatic salts as building blocks for dual-fluorescence intracellular probes. *ChemPhotoChem* **2017**, *1* (10), 442-450.
